# Assessing the fitness of Epstein-Barr virus following its reactivation

**DOI:** 10.1128/jvi.00626-25

**Published:** 2025-05-30

**Authors:** Yen-Fu Adam Chen, Bianca Mocanu, Ezgi Akidil, Dagmar Pich, Josef Mautner, Bill Sugden, Wolfgang Hammerschmidt

**Affiliations:** 1 EBV Vaccine Development, Helmholtz Zentrum München, German Research Center for Environmental Health, Munich, Germany; 2 Partner site Munich, German Centre for Infection Research (DZIF) 459706, Munich, Germany; 3 McArdle Laboratory for Cancer Research, University of Wisconsin—Madison 219455 , Madison, Wisconsin, USA; Lerner Research Institute, Cleveland Clinic, Cleveland, Ohio, USA

**Keywords:** Epstein-Barr virus, EBV, herpesvirus, mutagenesis, fusion assay, physical particles, infection, virus titer, viral fitness

## Abstract

Epstein-Barr virus (EBV) is usually maintained latently on passaging in cell culture, as are most herpesviruses *in vivo*. Its fitness, or its ability to infect and replicate in naïve cells, cannot be ascertained by serially passaging it in host cells because no identified cell line initially supports a productive infection by it. Yet its fitness is critical to EBV’s remarkable success as a human pathogen. We have, therefore, developed multiple approaches to assess EBV’s fitness upon being reactivated from its familiar state of latency. We established and tested expression plasmids for 77 viral genes and a set of shRNAs targeting 25 viral genes to measure how increasing and decreasing their levels affected the fitness of the released stocks of virus. Four of their properties were then analyzed: (i) their concentrations of physical particles, (ii) their binding to the CD21 receptor on a B-cell line, (iii) their entry into human primary B cells, and (iv) their infectious titers. These analyses identified multiple EBV genes whose altered levels of expression altered the biological activities of the released virus. These measurements revealed, though, an unexpected insight into the robustness of EBV produced from latently infected cells. EBV is amazingly resilient to any increased expression of its genes. The levels expressed in cells, as they support an induced productive infection, therefore are close to optimal for the fitness of the released virus.

**IMPORTANCE:**

Populations of viruses accumulate mutations while being propagated. While most mutations are neutral or disadvantageous, some confer on the variant a selective advantage, increasing its infectivity. These variants can be identified by serial passaging virus stocks, allowing those with increased fitness to predominate. This approach does not work for Epstein-Barr virus (EBV), for which no identified cell line initially supports its productive infection. How mutations accumulate in EBV as it is propagated latently to affect its fitness was unknown. We have devised an approach to assess EBV’s fitness upon being reactivated. Our findings suggest that EBV during its many latent generations has maintained a strikingly robust productive fitness.

## INTRODUCTION

Viral fitness is complex; “an experimentally useful approximation to fitness is the relative ability to produce stable infectious progeny in a given environment” (1). Experimentally, viral fitness is usually assessed as “replicative fitness,”" in which the levels of viral progeny are measured following rounds of productive infection in cell culture. Such measurements do not work for Epstein-Barr virus (EBV). This γ-herpesvirus does not infect cells productively; rather, it establishes a latent infection in them, which can only yield virus production as the daughter cells proliferate and acquire an altered state of viral chromatin (2). This latent to productive switch raises a fascinating problem in understanding EBV’s fitness. This and all herpesviruses are usually maintained *in vivo* latently so that their genome synthesis is carried out by cellular machinery relatively free of errors. As these viruses enter their productive phases, they will have accumulated mutations introduced by cellular synthetic machinery but not the frequent mutations generated by error-prone, viral synthetic machinery. Therefore, the selective pressures on viruses, such as herpes simplex virus 1 (HSV-1), to optimize the different steps during repeated rounds of infection in cell culture (3) are not exerted on EBV during its latency. However, selective pressures on latently infected cells will limit their genotypic variation, potentially imposing a genetic bottleneck on them akin to plaquing virus stocks. For example, variants of EBV that increase the survival or growth rate of infected cells will outgrow those that do not, but these traits need not make the resident EBV more fit to infect cells upon its maturation and release. We expected that this genetic bottleneck would lead to the viruses maintained in latently infected cells being potentially less fit in their infectivity and transforming ability.

We have developed a new approach allowing examination of the fitness of this γ-herpesvirus as it enters its productive cycle from latency. The levels of expression of individual genes were altered, the latently infected cells induced to enter their productive phase, and the biological activities of the viral progeny subsequently assessed. Increasing and decreasing the levels of expression of viral genes during an induced escape from latency allow an appraisal of any functional defects in the released virus. To gauge the fitness of the produced virus, we measured the number of particles released, the competence of the released virus to enter primary B cells, a natural host cell for EBV infection, and the number of infectious progeny released.

We chose a cell line, 2089 HEK293, that maintains EBV under selection, has been propagated for hundreds of generations, and has gone through multiple bottlenecks of being cloned (4–6). These cells release progeny virus upon transfection with a plasmid encoding BZLF1, but no apparent selection has been placed on them for their levels of expression of viral genes. The only known selective pressures on them have been to proliferate in cell culture and to survive in the presence of the antibiotic, hygromycin B, to which the resident EBV encodes resistance.

The 2089 HEK293 cell line harbors a cloned variant of the B95-8 strain of EBV (7), which might limit the interpretation of the data. We therefore extended our experiments to HH514 cells, a subclone of the parental P3HR1 Burkitt lymphoma cell line (8, 9). This cell line was demonstrated to release infectious EBV upon ectopic expression of BZLF1 (10), similar to 2089 HEK293 cells in sufficient quantity, making it compatible with our experimental approach here.

**Fig 1:**
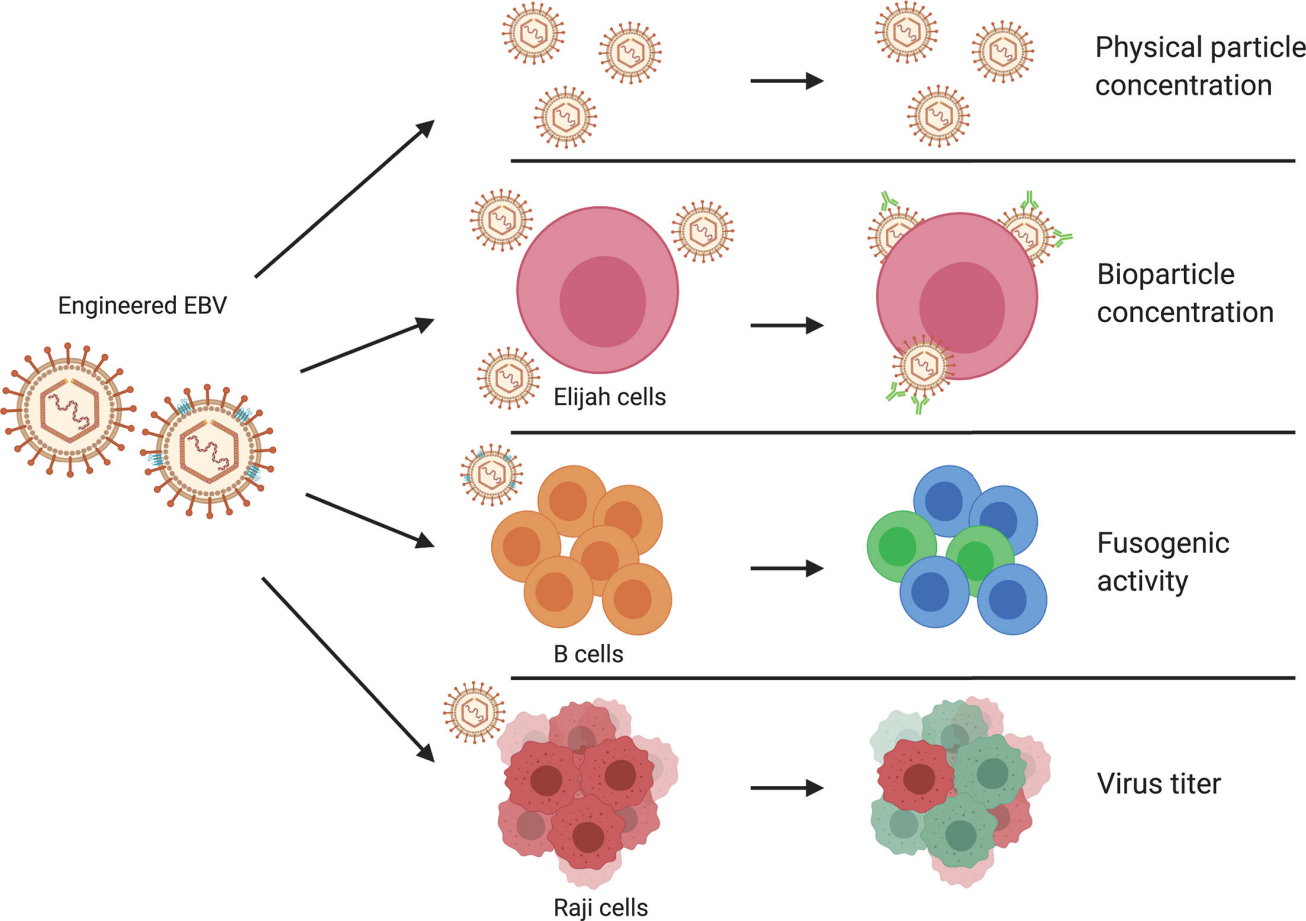
Experimental strategy of analyzing the composition of engineered EBV stocks and their virus-cell interactions. EBV stocks from the EBV producer cell line 2089 were collected 3 days after transient co-transfection of a BZLF1 expression plasmid together with individual expression plasmids from a panel of 77 different EBV genes. Alternatively, the EBV producer cells were co-transfected with the two plasmids plus a CD63:β-lactamase (BlaM) reporter plasmid to equip virus particles with an enzyme to study viral fusion with target cells. Different individual virus stocks were analyzed for their physical particle concentration using the nanoparticle tracking analysis instrument, ZetaView PMX110. Elijah cells were used to assess bioparticle concentration in the virus stocks via cell surface binding and subsequent analysis of bound particles by flow cytometry. Virus particles engineered to contain the CD63:β-lactamase reporter protein were analyzed for their fusogenic activity with human primary B cells as targets by flow cytometry. Raji cells were infected with virus stocks to measure the concentration of infectious virus particles conferring green fluorescence protein gene in the cells analyzed by flow cytometry. Similarly engineered virus stocks were also obtained from HH514 cells, a Burkitt lymphoma cell line infected with EBV. These HH514 EBV stocks were tested for their fusogenic activity with Daudi cells employing the split nanoluciferase system. The diagram was created with the help of BioRender.com (https://biorender.com/).

Earlier findings led to the expectation that altering the levels of viral genes would identify genes whose expression limited a robust productive infection. For example, the increased expression of the BALF4 gene increased the number of infectious progeny released from cells induced to support EBV’s lytic cycle by sixfold (11). To study this form of fitness comprehensively, we have increased the levels of 77 individual viral genes, decreased the levels of a subset of 25 viral genes, induced EBV to support virus production in 2089 HEK293 cells, and then assessed the released virus systematically. The resulting virus stocks were characterized for four properties: (i) their physical particle concentration was measured with a nanoparticle tracking analysis (NTA) instrument, (ii) the ability of the virus particles to bind solely to the CD21 receptor on a B-cell line was quantified, (iii) the ability of virus particles to fuse with or enter human primary B cells was directly analyzed in a newly developed functional assay, and (iv) the infectious titers of the released virus were assayed (Fig. 1).

These experiments identified multiple genes whose altered levels of expression led to detectable changes in the biological activities of the released virus. The sum of these measurements revealed, though, an unexpected insight into the robustness of EBV produced from latently infected cells. EBV is amazingly resilient to increased expression of its genes; neither the quantity nor the quality of the released virus was dramatically affected by most such changes.

## RESULTS

### Establishment of an expression plasmid library with 77 individual viral genes and generation of EBV stocks

Individual open reading frames and known genes of EBV were cloned into the expression vector plasmid pcDNA3 to establish a comprehensive EBV library with all known and hypothetical protein-encoding genes (Table S1). Single viral proteins could now be ectopically introduced in 2089 EBV producer cells concomitant with inducing EBV’s lytic phase as described (6). The EBV library used in this study is adapted from a previously published library designed for screening antiviral T-cells (12). In the original library, all viral genes were tagged with epitopes, and the expression of these viral proteins was confirmed in HEK293 cells (12). For our experiments, we removed all epitope tags to preserve the natural function of the viral genes. As a result, we could not detect the expression of all viral proteins, but we were able to confirm the expression of 10 viral proteins with available monoclonal antibodies. This approach gives us confidence that all 77 unmodified viral genes are expressed upon transient transfection in 2089 EBV producer cells.

The standard protocol to produce virus stocks is based on the 2089 EBV producer cell line, which are HEK293 cells with a recombinant EBV B95-8 strain genome encoding a green fluorescence protein (GFP; [4]). Virus production relies on transient transfection of the BZLF1 expression plasmid p509 (13). To test individual members of the viral expression plasmid library, 2089 EBV producer cells were co-transfected with an expression plasmid encoding the BZLF1 gene (p509) together with a plasmid encoding one of the 77 individual viral genes. An “empty” expression vector plasmid was co-transfected with BZLF1 as control as well as reference for subsequent normalization of data. Virus stocks co-transfected with BZLF1 and BALF4 were used as positive controls (11). After 3 days, the virus supernatants were harvested, and the resulting virus stocks were tested. All experiments were done in triplicate.

### Quantitative analysis of extracellular vesicles in cell culture media from non-induced and induced 2089 EBV producer cells

**Fig 2:**
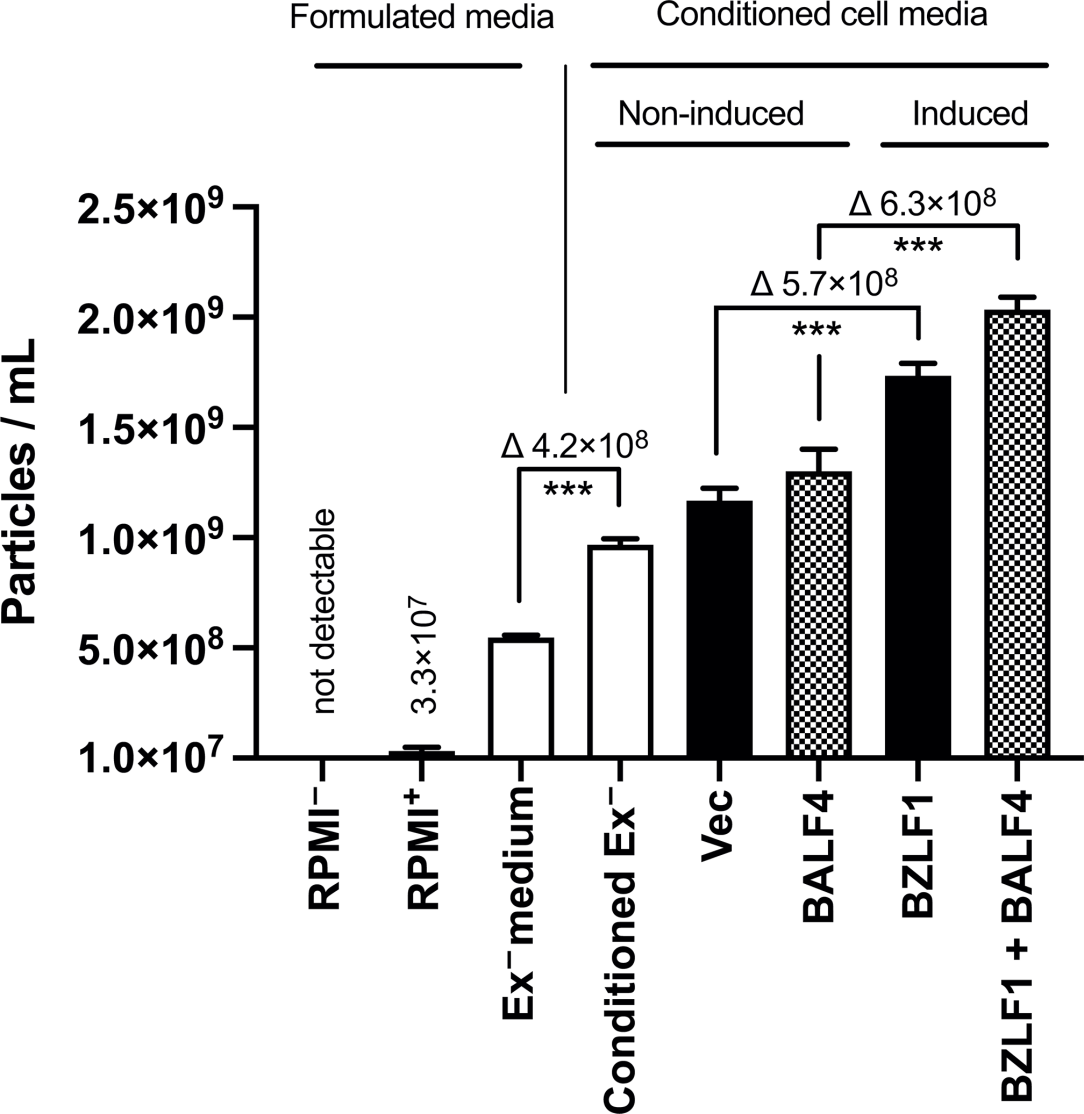
Concentration of physical particles in different formulated cell culture media and in conditioned cell culture media from non-induced and induced 2089 EBV producer cells. Concentrations of physical particles were determined with the aid of nanoparticle tracking analysis using the ZetaView PMX110 instrument. Formulated media are different cell culture medium preparations based on standard RPMI1640, which are as follows: RPMI^–^, plain, non-supplemented commercial RPMI1640 medium (Gibco); RPMI^+^, commercial RPMI1640 medium supplemented with penicillin-streptomycin stock, sodium pyruvate, sodium selenite, and α-thioglycerol; Ex^–^ medium, RPMI^+^ with 10% fetal bovine serum and the supplements listed above after ultracentrifugation (100,000 *g*, 4°C for more than 16 h as described in Materials and Methods); Conditioned Ex^–^, supernatant from non-induced 2089 EBV producer cell line cultured in Ex^–^ medium for 3 days; Vec, 2089 EBV producer cells transfected with pCMV control plasmid DNA (0.5 µg; p6816) using 3 µL PEI MAX cultivated in Ex^–^ medium for 3 days; BALF4, same as “Vec,” but the cells were transfected with 0.5 µg of the BALF4 expression plasmid p6515; BZLF1, same as “Vec,” but the cells were transfected with 0.5 µg of the BZLF1 expression plasmid p509 to induce EBV production; BZLF1 + BALF4, same as “Vec,” but the cells were co-transfected with 0.25 µg each of the expression plasmids p509 and p6515 coding for BZLF1 and BALF4, respectively. Mean and standard deviation of three replicates are shown. ***P ≤ 0.001.

Standard cell culture medium contains fetal bovine serum (FBS), which introduces extracellular vesicles (EVs) and related particles ranging from 50 to more than 200 nm. We analyzed the concentration of EVs in plain RPMI1640, formulated cell culture medium based on RPMI1640, and in conditioned cell culture supernatants using a ZetaView PMX110 instrument capable of NTA. The results are shown in Fig. 2. Fully supplemented, but partially, EV-depleted RPMI1640 cell culture medium contains approximately 5 × 10^8^ physical particles per milliliter, which are of bovine origin. This formulated cell culture medium was termed “Ex^–^ medium” in which non-induced 2089 EBV producer cells were cultivated for 3 days. This “Conditioned Ex^–^ medium” contained considerably more EVs. Transient transfection of two plasmid control DNAs (indicated “Vec” and “BALF4” in Fig. 2) followed by cultivating the non-induced cells for 3 days increased the EV concentration further. Finally, EBV’s lytic phase was induced by transfecting expression plasmids encoding BZLF1 only or in combination with BALF4, which caused a significant increase of physical particles of 5.7 × 10^8^ and 6.3 × 10^8^ EVs per milliliter, respectively, compared to the corresponding non-induced controls after 3 days (Fig. 2).

This analysis shows that conventional cell culture medium contains substantial numbers of EV even after depleting bovine EVs. Spent, conditioned medium contains more EVs, which all cells spontaneously release. After lytic induction of 2089 EBV producer cells, EV concentrations increased further, suggesting that induced, virus-releasing cells shed more physical particles (in the order of 5 × 10^8^ per milliliter) than non-induced cells.

### Effects of individual EBV genes on physical particle concentration

**Fig 3:**
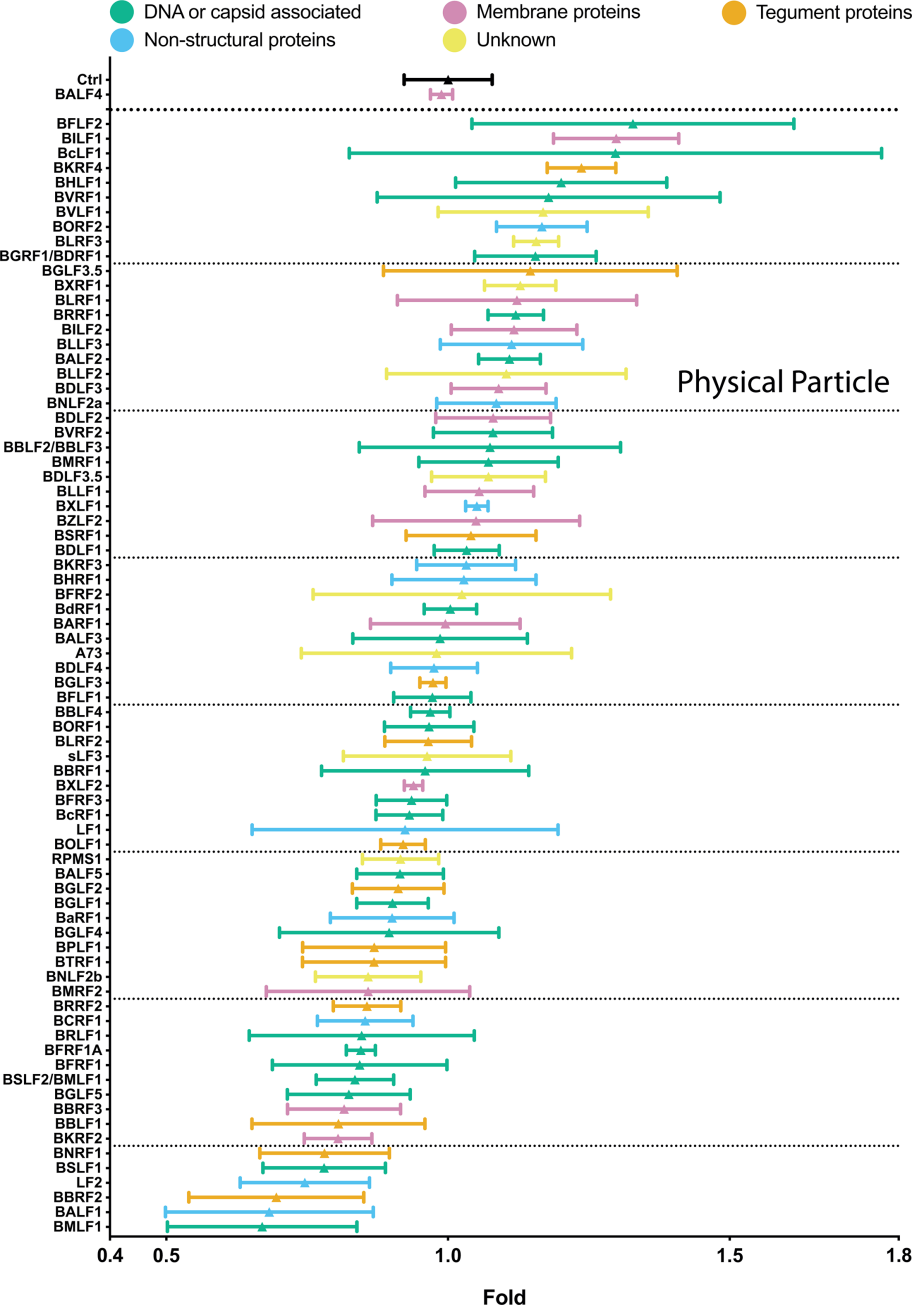
Comparison of physical particle concentrations in virus samples generated by co-transfection of BZLF1 and single expression plasmids from a panel of 77 EBV genes including two controls. EBV stocks were analyzed for their physical particle concentration by nanoparticle tracking analysis. NTA was performed with the ZetaView PMX110 instrument, and the images were analyzed with the ZetaView 8.04.02 software. Standard calibration beads were used to confirm the range of linearity. The *y*-axis lists the transfected individual EBV genes co-transfected with BZLF1. An empty pCMV vector plasmid plus the BZLF1 plasmid was co-transfected as reference (Ctrl). The positive control encompasses supernatants obtained after co-transfection of both BZLF1 and BALF4 expression plasmids. The number of physical particles in the range of 100–200 nm contained in the supernatants of cells was analyzed and normalized to the reference sample (Ctrl). The results are arranged in descending order and are classified according to five functional groups and color coded as indicated. Mean and standard deviation of three biological replicates are shown. The horizontal lines indicate groups of 10 viral genes for better visualization. The viral gene designated sLF3 represents a version of LF3 with a reduced number of internal repeats.

To test all 77 individual members of the viral expression plasmid library, 2089 EBV producer cells (4) were transiently co-transfected with plasmid DNAs encoding the BZLF1 gene and 1 of the 77 individual viral genes. BALF4 was used as a positive control. Three days after transfection, the virus supernatants were assayed for their concentrations of physical particles by NTA. Fig. 3 shows the results of 77 viral genes plus controls in descending order.

The increase in the mean particle concentration in the different virus stocks varied between 0.7- and 1.3-fold. Some virus stocks showed inconsistent numbers of physical particles from one experiment to the next, documented by their wider standard deviation (e.g., BcLF1, BVRF1, BGLF3.5, LF1, among others) for unknown reasons. Statistical analyses are provided in Table S5.

We considered if there is a link between physical particle numbers and molecular functions of the genes tested, and if their expression timing could correlate with these measurements. We depicted the data in a color-coded fashion in Fig. 3; Fig. S1 to search for similarities in these measurements among genes with related functions. Neither the number of particles in a given supernatant nor the timing of expression of the transfected gene associated with that supernatant correlated with the function of the gene.

### Measuring viral bioparticle concentrations

**Fig 4:**
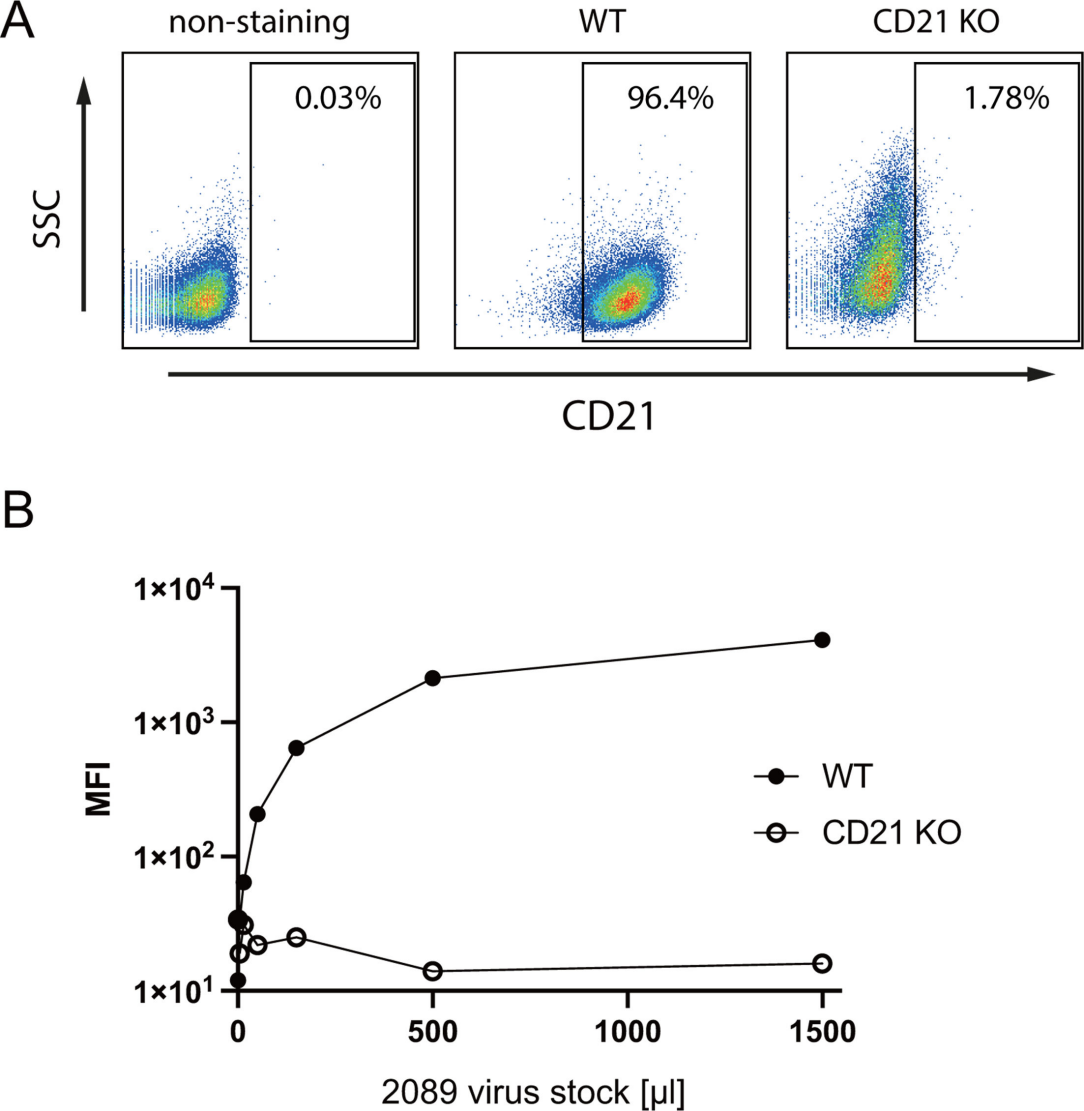
CD21 is essential for virus-cell adhesion. The two alleles of the surface receptor CD21 were deleted in Elijah cell chromatin using pre-formed CRISPR-Cas9 ribonucleoprotein complexes with two individual CD21-targeting guide RNAs and a recombinant Cas9 nuclease. The cells were further sorted for CD21-negative cells. (**A**) CD21 expression levels and knockout efficiency of Elijah cells were evaluated by flow cytometry using an APC-coupled CD21-specific antibody. Left panel, wild-type (WT) Elijah cells without antibody staining; middle, wild-type Elijah cells stained with the fluorochrome-coupled CD21 antibody; right, CD21 expression level in sorted CD21-negative Elijah cells after CRISPR-Cas9-mediated knockout (CD21 KO). (**B**) Binding activity of 2089 EBV stocks was analyzed with WT and CD21 KO Elijah cells. Furthermore, 2 × 10^5^ Elijah cells were incubated with 0, 5, 15, 50, 150, 500, and 1,500 µL virus stocks at 4°C for 3 h. The cell-surface-bound virus particles were detected with an Alxea647-coupled anti-gp350 antibody, and mean fluorescence intensities (MFI) were recorded by flow cytometry and plotted as a function of virus dose.

Viral infectivity is the successful endpoint of virion production, which initiates with virus binding to target cells as the first step. We measured this key function using cellular binding assays with Elijah cells as target, a human B-cell line derived from a case of Burkitt lymphoma (14). The binding assay was introduced in 2010 (15) and is a proxy for virus attachment to human B cells, which includes EBV’s cognate CD21 receptor, the complement receptor 2 (CR2) (16, 17) and is mediated by the viral gp350 protein encoded by EBV’s open reading frame BLLF1. It has remained uncertain whether the binding of EBV particles to the surface of Elijah cells solely depends on the interaction of gp350 with CD21 or whether additional viral glycoproteins contribute to surface binding as suggested earlier (18). Therefore, we deleted *CD21* using CRISPR-Cas9 technology (19) in Elijah cells (Fig. 4A) and found that EBV binding was no longer detectable even with exceedingly high virus doses (Fig. 4B). This finding shows that the binding of viral particles to Elijah cells is solely dependent on CD21, a receptor of EBV required for viral adhesion to B cells (16, 20–22). Our finding also indicates that the Elijah cell binding assay exclusively records gp350-bearing particles, which include intact virions but also non-infectious particles such as defective viruses, virus-like particles, and gp350-decorated EVs, which are released from lytically induced 2089 EBV producer cells. As the Elijah cell binding assay cannot differentiate between various gp350-containing vesicle species, we collectively call them “bioparticles” throughout this study.

**Fig 5:**
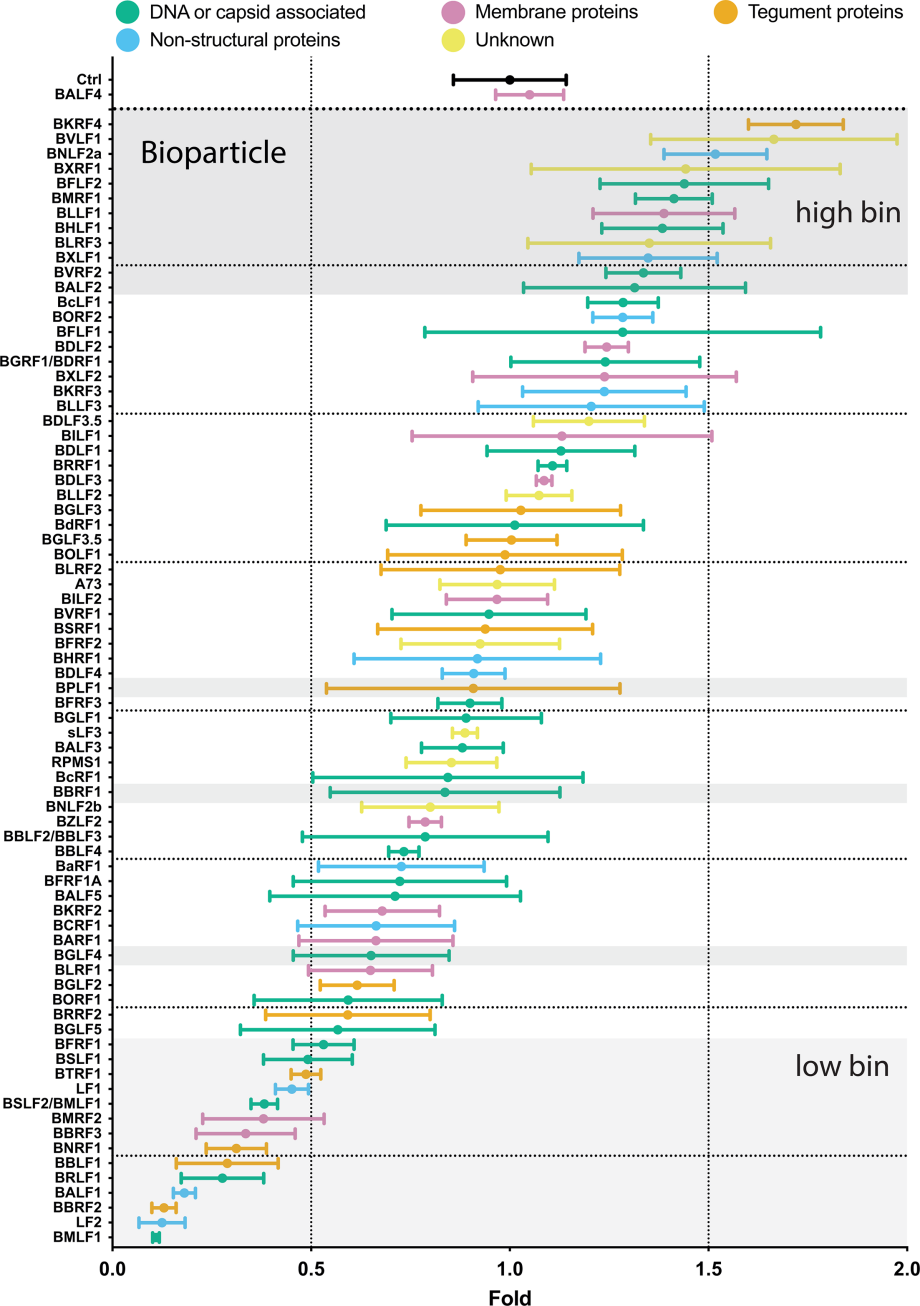
Comparison of bioparticle concentrations in virus samples generated in 2089 EBV producer cells by co-transfection of BZLF1 and individual expression plasmids from a panel of 77 EBV genes. Virus production and generation of samples were identical as described in the legend of Fig. 3. The amount of gp350-positive particles bound to Elijah cells was quantified using a gp350-specific, fluorochrome-coupled monoclonal antibody and flow cytometry. The ratios of mean fluorescence intensity values of individual samples vs the MFI value of the reference sample (Ctrl) were calculated and are provided on the *x*-axis. An empty pCMV vector plasmid co-transfected with the BZLF1 plasmid p509 served as reference (Ctrl). The *y*-axis lists the transfected individual EBV genes. Ratios are arranged in descending order. An expression plasmid encoding BALF4 served as a positive control. Shaded areas at the top and bottom highlight groups of viral genes termed “high bin” and “low bin,” respectively. Three singly highlighted genes (BPLF1, BBRF1, and BGLF4) were randomly picked as further candidates. EBV genes are classified according to five functional groups as indicated. Mean and standard deviation of three biological replicates are shown. The two vertical lines indicate 0.5- and 1.5-fold ratios, and horizontal lines indicate groups of 10 viral genes for better visualization. The viral gene designated sLF3 represents a version of LF3 with a reduced number of internal repeats.

The previously tested EBV stocks and controls shown in Fig. 3 were analyzed for bioparticle binding to the surface of Elijah cells. Practically, Elijah cells and individual virus stocks were incubated for a limited time at low temperature to allow binding to Elijah cells. Binding was detected with the gp350-specific, fluorochrome-coupled monoclonal 6G4 antibody. After washing, the cells were analyzed by flow cytometry for their raw values of mean fluorescence intensity (MFI). Virus stocks generated with the 2089 EBV producer cells after co-transfection of an empty vector plasmid together with BZLF1 (p509) served as the reference control (Ctrl) and standard for calculating the ratios of MFI values as shown in Fig. 5; Fig. S2. Virus stocks generated by co-transfection of the BALF4-encoding expression plasmid p6515 together with p509 served as an additional control because the number of viral particles obtained after ectopic expression of BALF4 was expected to be unaltered (11).

The virus stocks produced by co-transfecting BALF4 and BZLF1 showed no difference in this assay (Fig. 5). Other virus stocks showed a narrow range of variability of about a factor of 1.5–0.5 similar to that for physical particles observed in Fig. 3. Only a small group of 13 virus stocks (BSLF1 - > BMLF1) showed a reduction below a factor of 0.5. As in Fig. 3, the color codes illustrate the five functional groups of viral proteins in Fig. 5. Again, no discrete functional characteristic was obvious. When the results were color coded according to gene expression timing regarding early, late genes, or genes with unknown timing, no special contribution of the three different classes could be found (Fig. S2). This result indicates that viral genes expressed throughout the entire lytic phase of virus production contribute variously to the synthesis of viral bioparticles. For example, BKRF4, BVLF1, and BNLF2a, which represent a tegument protein, a protein with unknown function, and a non-structural protein, respectively, are at the high end of synthesizing bioparticles and all belong to different groups for their timing of expression (Fig. S2).

### Ectopic expression of certain viral genes increases or decreases virus titers, but BALF4 is unique

**Fig 6:**
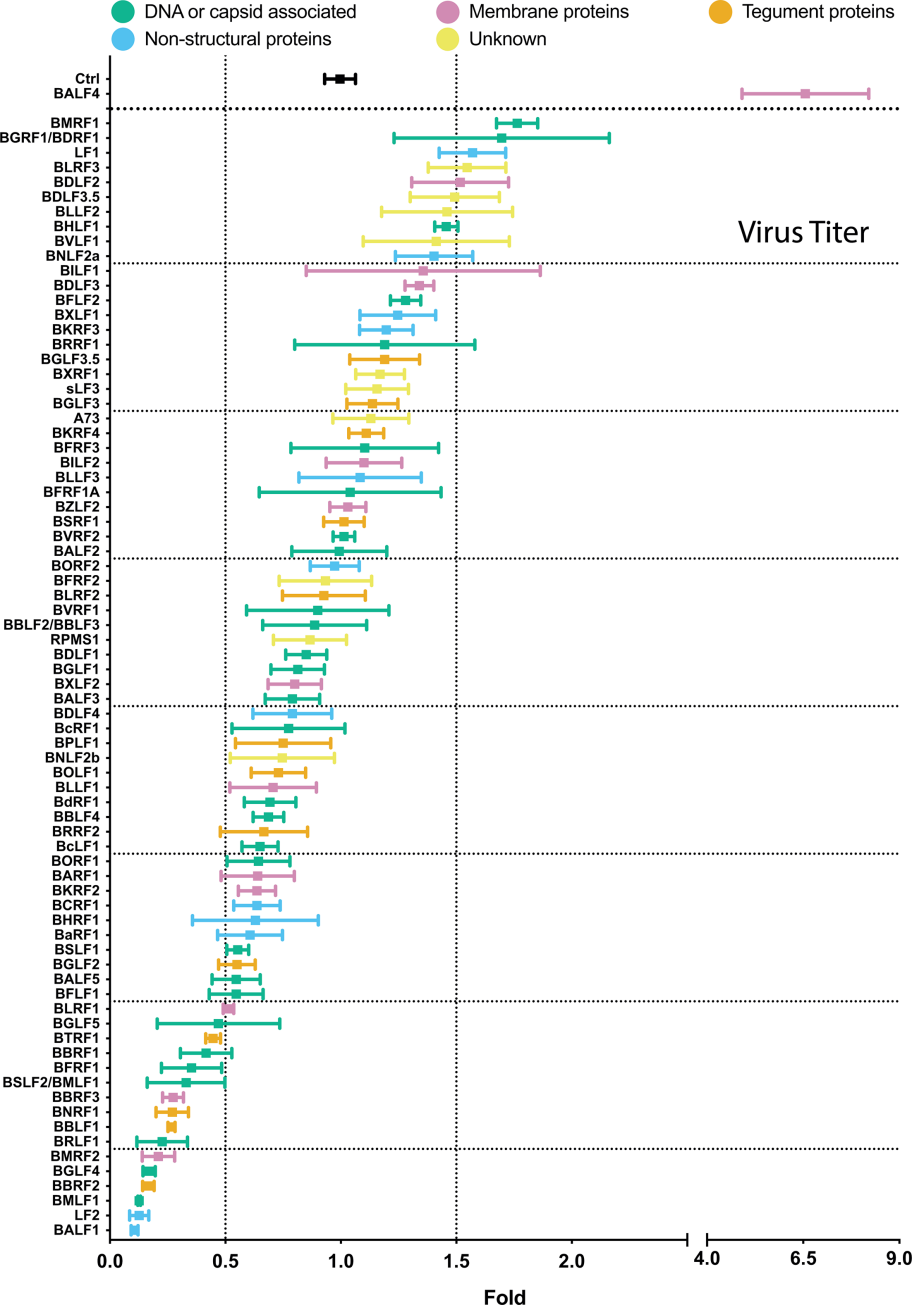
Comparison of virus titer of stocks generated by co-transfecting 2089 EBV producer cells with BZLF1 together with single expression plasmids from a panel of 77 EBV genes. 2089 EBV producer cells were co-transfected with BZLF1 and single expression plasmids encoding the denoted viral genes. Virus-containing supernatants were harvested 3 days later and used to infect Raji cells. After 3 days, the infected Raji cells were investigated by flow cytometry analyzing the expression of green fluorescence protein. The *y*-axis lists the individual EBV genes transfected in combination with BZLF1. The BZLF1 (p509) expression plasmid co-transfected with p6816, an empty pCMV vector plasmid served as reference and control (Ctrl). The titers of infectious EBV stocks according to the percentage of GFP-positive Raji cells were normalized to the reference sample (Ctrl), which was set to 1. The results are listed in descending order. EBV genes are color coded according to five functional groups as indicated. Mean and standard deviation of three biological replicates are shown. Vertical lines indicate 0.5- and 1.5-fold ratios. The horizontal lines indicate groups of 10 viral genes for better visualization. The viral gene designated sLF3 represents a version of LF3 with a reduced number of internal repeats.

To analyze the infectivity of the virus stocks, we used Raji cells, a human Burkitt lymphoma cell line (23), which provides a functional assay. The fraction of EBV-infected Raji cells was identified by green fluorescence protein expression and quantified by flow cytometry. The results from three independent infection experiments were normalized to the respective controls, which were set to 1. The ratios of the 77 tested combinations plus controls were arranged in descending order and color coded as before (Fig. 6; Fig. S3). The viral BALF4 gene co-transfected with BZLF1 served as a positive control. The results in Fig. 6 revealed that ectopic expression of BMRF1, BGRF1/BDRF1, LF1, BLRF3, BDLF2, or BDLF3.5 yielded virus titers increased by a factor of 1.5 or more, indicating that only six genes besides BALF4 support higher virus titers. Two of these genes encode DNA- or capsid-associated proteins, one is a membrane protein, one is a non-structural protein, and two genes have unknown functions. No tegument protein-encoding gene is among this list. Expression timing did not correlate with titers, either (Fig. S3). The majority of viral genes tested, about 60 in total, showed little variation in virus titers. In contrast, the last 15 expression plasmids (BGLF5, BTRF1, BBRF1, BFRF1, BSLF2/BMLF1, BBRF3, BNRF1, BBLF1, BRLF1, BMRF2, BGLF4, BBRF2, BMLF1, LF2, BALF1) co-transfected with BZLF1 led to virus titers reduced by a factor of 2 or more (Fig. 6). Again, there is no clear functional attribute, nor does expression timing correlate with these decreases.

Transient expression of individual EBV genes might affect cell survival of 2089 producer cells and could thus cause a reduction of released bioparticles or infectious virus. We tested this possibility (Fig. S4) but did not find evidence of a toxic effect in transiently transfected cells using 11 expression plasmids of genes that belong to the “low bin” group in Fig. 5.

Taken together, ectopic expression of individual viral genes resulted in small differences in the titers of the virus stocks. The positive control BALF4 increased the infectivity of the virus stocks by a factor of 6.5, which exceeds the effects of other viral genes (Fig. 6). Remarkably, the B95-8 laboratory strain lacks the viral LF1 gene, suggesting that it contributes to previously unknown supportive functions during virus synthesis or enhances viral infectivity. A larger group of genes exhibits repressive functions and shows a clear inhibition on virus synthesis or virus infectivity.

### Statistical analysis supports a correlation of physical particle and bioparticle concentrations and virus titers

The findings shown in Fig. 3 to 6 demonstrate the characteristics of three quantitative parameters (physical particle, bioparticle concentrations, and virus titers) assayed in 77 individual EBV stocks. The mean values of the three measurements of the virus stocks were assembled and revealed, using the Spearman rank correlation, a high overall correlation (Fig. S5A).

The statistical analysis indicates that individual viral genes can have a demonstrable impact on all three basic parameters investigated. No correlations regarding expression timing or functional classes emerge, demonstrating that HEK293 cells support virus synthesis efficiently, with the levels and timing of viral gene expression being optimal in most cases (Fig. S5B and C).

### Selection, design, and validation of shRNA candidates targeting 25 viral gene**s**

To concentrate on potentially interesting viral genes, we chose candidates from both ends of the ranked candidates identified in the experiments shown in Fig. 5 and determined if members of the “high bin” group are essential for virus production as measured in infection experiments in Fig. 6. Conversely, we asked if members of the “low bin” group of viral genes might inhibit virus synthesis and, hence, repress virus titers because, for example, some genes might act as regulators controlling and thus limiting virus production.

As shown in Fig. 5, the “high bin” group encompasses 10 candidates (BKRF4, BVLF1, BNLF2a, BXRF1, BFLF2, BMRF1, BHLF1, BXLF1, BVRF2, and BALF2), and the “low bin” group lists 11 viral genes (BFRF1, BSLF1, BTRF1, BMRF2, BBRF3, BNRF1, BBLF1, BRLF1, BALF1, BBRF2, and BMLF1). Three randomly selected genes, BPLF1, BBRF1, and BGLF4, were chosen from the large middle group. Certain genes were excluded from the “top bin” and “low bin” groups for various reasons. LF1 and LF2 were excluded since the B95-8 EBV strain in the 2089 EBV producer cell line does not encode them. Their ectopic expression in the 2089 producer cells was informative, but their lack precludes a knockdown strategy, as explained below. BLLF1 (gp350) was excluded because its ectopic expression directly affected the quantitative analysis of bioparticles. Also, BLRF3 was excluded from the “high bin” group as it is part of EBNA3 and likely not directly involved in EBV’s lytic phase.

To test the 25 selected viral genes for their possible essential or regulatory roles during lytic infection, we used an shRNA knockdown strategy to repress individual viral gene products to study their contribution during virus and bioparticle production. The design of the shRNAs and their functional evaluation are shown in Fig. S6 and are described in the Materials and Methods section.

### Virus titers in supernatants from 2089 EBV producer cell lines stably transduced with sets of shRNAs directed against selected EBV transcripts

**Fig 7:**
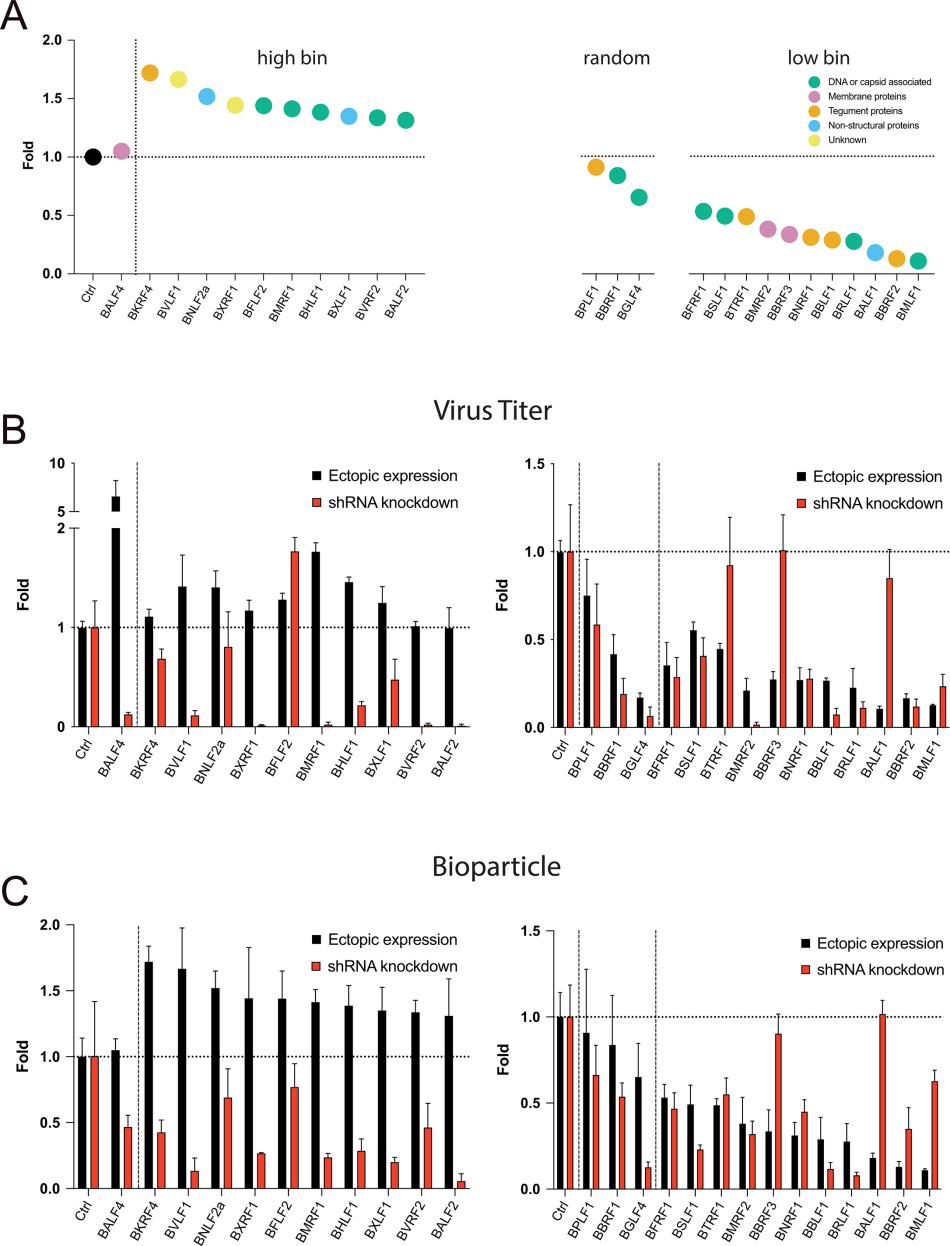
Viral titers and bioparticles in supernatants generated after co-transfection of BZLF1 and single viral genes into 2089 EBV producer cell lines stably transduced with sets of 3 shRNAs directed against 25 individual viral transcripts. (**A**) Recapitulation of selected data shown in Fig. 5. Three groups of viral genes are shown that were picked randomly (“random”), or increased (“high bin,” left panel) or decreased (“low bin,” right panel) the yield of bioparticles when co-transfected together with BZLF1 into the EBV producer cell line 2089. The EBV genes are arranged in descending order, and the color codes depict the different functions of viral proteins as in Fig. 5. (**B**) Parental 2089 EBV producer cells were stably transduced with sets of 3 shRNAs each targeting 25 viral transcripts. Controls encompass the EBV producer cell line 2089 stably transduced with the empty pCDH shRNA expression vector (Ctrl). The viral genes termed “high bin” and “low bin” groups are shown in the left and right panels, respectively. The individual cell lines were transiently transfected with the BZLF1 expression plasmid. The virus supernatants were harvested 3 days after transfection and used to infect Raji cells. After 3 days, the fraction of GFP-positive Raji cells was determined by flow cytometry, and virus titers (GFP-positive Raji cell unit titers) were calculated. Supernatants harvested from the control cell lines are shown on the left side of the graph with the “high bin” group of genes separated by a vertical dotted line from the test samples. The “low bin” group is shown on the right, separated from the control, and includes the three “random” genes separated by a dotted vertical line. Virus titers of supernatants obtained from the 25 individual shRNA-expressing EBV producer cell lines (red columns; shRNA knockdown) analyzed in panel B are compared with virus titers found in supernatants from the parental EBV producer cell line 2089 upon transient expression of viral genes (black columns; ectopic expression). Mean and standard deviation of three biological replicates are shown. (**C**) Virus stocks analyzed in panel B were tested for their bioparticle concentration in the Elijah cell binding assay. Bioparticle concentrations found in supernatants from parental 2089 EBV producer cells upon ectopic expression of single viral genes (as analyzed in Fig. 5; black columns; ectopic expression) are compared with results obtained from 2089 EBV producer cells stably transduced with sets of shRNAs (red columns; shRNA knockdown). Mean and standard deviation of three biological replicates are shown.

Twenty-four EBV producer cell lines stably transduced with sets of three shRNA vectors each were established under co-selection with puromycin and hygromycin B to ensure shRNA expression and maintenance of the EBV 2089 genome, respectively. We selected sets of three shRNAs per transcript to increase the knockdown effect on the individually targeted mRNAs. Controls included 2089 EBV producer cells transduced with the empty shRNA vector backbone (p6924), only, as well as cells encoding triple sets of shRNAs directed against GFP or BALF4 transcripts as tested in Fig. S6. The three graphs in Fig. 7A depict the selected genes, their functional attributes, and recapitulate bioparticle ratios obtained after the genes’ ectopic expression shown in Fig. 5. Virus stocks from the different 2089 EBV producer cell lines stably transduced with shRNA vectors were generated by transient BZLF1 transfection and quantified by infecting Raji cells as in Fig. 6. The results were arranged according to the “high bin” and “low bin” groups in Fig. 7B (red columns) and shown in comparison with ratios of virus titers when the viral genes were ectopically expressed in the parental 2089 EBV producer cell line (black columns).

In the left panel of Fig. 7B, ectopic expression of BALF4 yielded high virus titers, whereas its knockdown repressed virus titers to about 10%. These results validated the shRNA knockdown strategy and confirmed BALF4’s role as an essential gene (24) that boosts viral infectivity upon ectopic expression (11).

As expected, certain shRNA sets decreased virus titers dramatically, while others did not cause such a pronounced effect (Fig. 7B). The results indicated that certain viral genes are essential (BXRF1, BMRF1, BVRF2, BALF2, BMRF2) or, conversely, are dispensable (BFLF2, BTRF1, BBRF3 and BALF1) for the production of infectious virus stocks. Alternatively, it remains possible that the knockdown of the apparently dispensable genes was insufficient despite the data presented in Fig. S6A and B. Other shRNAs had a weak repressive phenotype (BKRF4, BNLF2a, BHLF1, BXLF1, BFRF1, BSLF1, BBLF1, and BRLF1) or barely affected production of infectious virus (BPLF1). Three shRNAs (BTRF1, BBRF3, and BALF1) in the “low bin” group (Fig. 5) did not increase virus production beyond wild-type level, although the ectopic expression of these genes was detrimental for the release of infectious EBV (right panel in Fig. 7B). These results suggested that the three genes also do not qualify as regulators of EBV’s lytic phase. Remarkably, the shRNA-mediated knockdown of BFLF2 seemed to induce virus titers detectably (Fig. 7B, left panel).

### Ectopic expression and shRNA knockdown of viral genes and transcripts—comparing virus titers and bioparticle concentration of 25 viral targets

Virus stocks from infection experiments with Raji cells (Fig. 7B) were also analyzed for their bioparticle concentrations using the Elijah cell binding assay. The results are summarized in Fig. 7C. Results from ectopic expression of single viral genes and the matching shRNA knockdown experiments are shown in black and red, respectively. The shRNA-mediated knockdown of BALF4 showed a decrease by half of bioparticle concentration, but a substantial reduction in the BVLF1 and BALF2 shRNA-expressing EBV producer cell lines was observed, which is in agreement with their low virus titers in Fig. 7B. In the right panel of Fig. 7C, the majority of supernatants from shRNA-expressing EBV producer cell lines did not have markedly different bioparticle concentrations (BBRF1, BFRF1, BTRF1, BMRF2, and BNRF1), whereas some had reduced numbers of bioparticles (BGLF4, BSLF1, BBLF1, and BRLF1). The shRNA-mediated knockdown of BBRF3 and BALF1 transcripts had no measurable effect compared to the reference (Fig. 7C, right panel). The 2089 producer cell line transduced with the set of three shRNAs directed against the GFP transcript did not affect bioparticle concentration significantly, validating our general approach.

Together, the shRNA strategy complements the results from ectopically expressed single viral genes with respect to bioparticle concentrations and infectious virus titers in Fig. 5 and 6, respectively. This shRNA strategy did not identify a viral master regulator.

### Analysis of the fusogenic activity of 25 virus stocks from the “high bin” and “low bin” groups

We developed a novel assay to measure the fusogenic activity of engineered extracellular vesicles (25), which we adapted recently to analyze the fusogenicity of EB virions using primary human B cells, EBV’s primary target cells, and plasmacytoid dendritic cells (26). The β-lactamase (BlaM) assay has been developed in the HIV field to detect fusion of HIV particles with T cells (27, 28). In this context, the enzyme is introduced into recipient cells by virus-mediated transfer to cut the β-lactam ring of the CCF4 substrate with which the cells are loaded. Cleavage of the CCF4 substrate alters its emission wavelength from 520 to 447 nm, which can be detected and differentiated by flow cytometry. The protocol has been constantly improved (29), and the method is applicable to detect transduction of BlaM when incorporated into either viruses or entities such as EVs (25, 26). Here, we fused the codon-optimized sequence of BlaM to the C-terminus of the tetraspanin CD63 transmembrane protein to locate BlaM in the tegument of EBV particles. The strategy leverages the fact that CD63, a protein originally from the host cell, is incorporated in the envelope of EBV virions.

**Fig 8:**
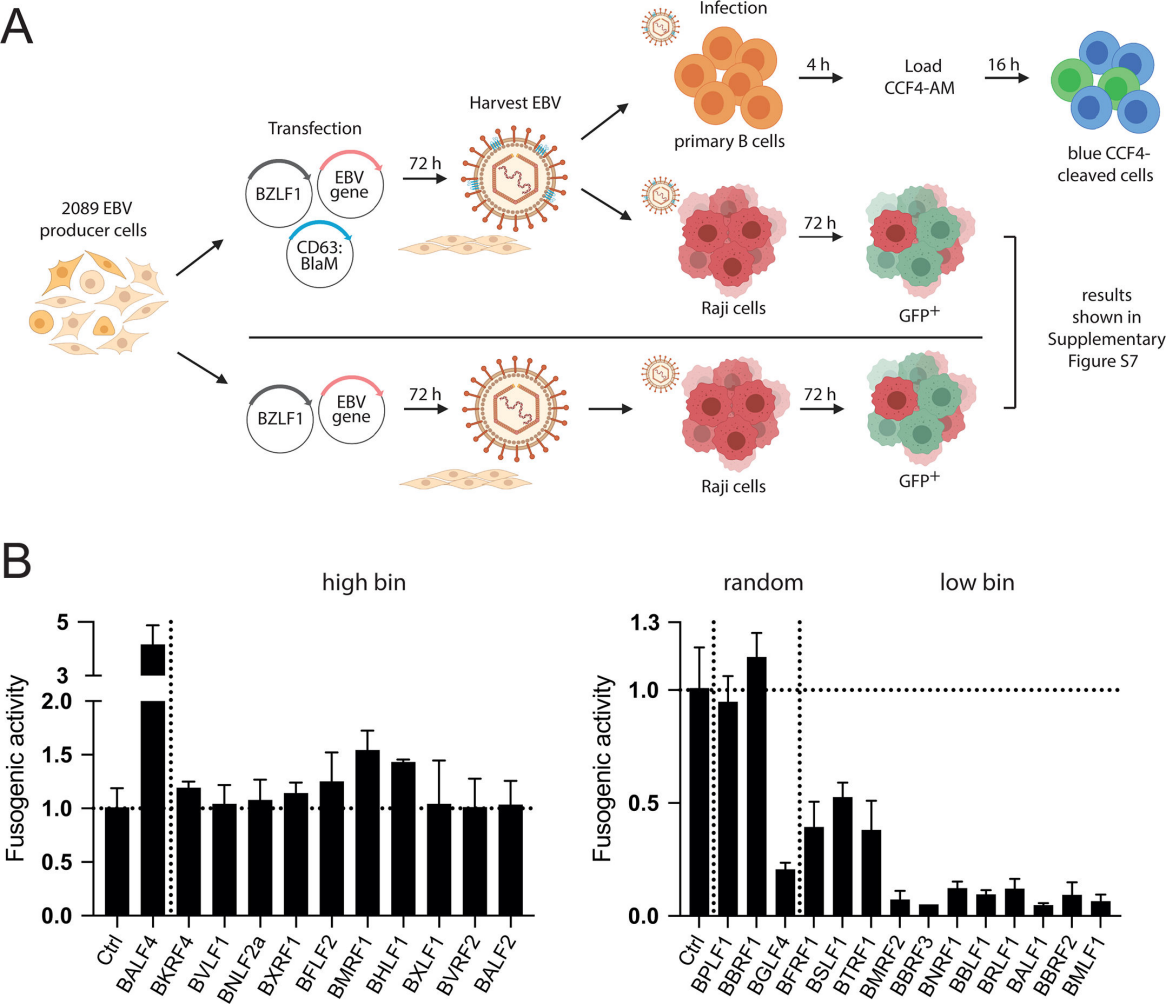
Analysis of engineered virus stocks using the BlaM fusion assay and primary human B cells as targets. (**A**) The flow chart depicts the experimental steps of the fusion assay (top pathway) and its comparison with the Raji cell-based test for infectivity (below, the results are shown in Fig. S7). (**B**) Results of the β-lactamase fusion assay with supernatants of the 2089 EBV producer cell line transiently transfected with 25 individual expression plasmids encoding viral genes of the “high bin,” “low bin” groups and three random genes. The cells were co-transfected with three plasmids as shown in panel A (plasmids encode BZLF1 [p509], CD63:BlaM [p7200], and 1 of the 25 selected EBV genes or controls). The resulting supernatants were tested on primary human B cells as targets. The readouts are based on the fraction of “blue” B cells with cleaved CCF4 substrate by flow cytometry and normalized to the control. Shown are the results from the “high bin” and “low bin” groups and three randomly picked viral genes separated by a vertical dotted line. The illustration was created using the BioRender.com source (https://biorender.com/).

To study virus fusion separately and independent of viral infectivity (which encompasses intracellular transport and *de novo* expression of viral proteins or the GFP reporter), the assay shown schematically in Fig. 8A was established. 2089 EBV producer cells were co-transfected with an expression plasmid-encoding CD63:BlaM (p7200) together with BZLF1 (p509) and single expression plasmids that encode the 21 individual viral genes of the “high bin” and “low bin” groups plus controls as defined in Fig. 5 and 7A. Human primary B cells isolated from adenoid tissue were incubated with CD63:BlaM-containing virus stocks for 4 h. Subsequently, the cells were loaded with CCF4 substrate and incubated at room temperature for 16 h. The fraction of cells with emission light shift was determined by flow cytometry. Virus stocks generated by co-transfection of BZLF1, an empty expression vector plasmid, and CD63:BlaM served to normalize the data depicted in Fig. 8B. The CD63:BlaM-equipped virus stocks were also used to determine their titers after Raji cell infection (Fig. S7). A virus stock generated by transient transfection of the 2089 EBV producer cells with only BZLF1 (p509) served as an additional control.

The left panel in Fig. 8B shows the fusogenic activities of 10 EBV stocks of viral genes (plus BALF4) that belong to the “high bin” group as defined in Fig. 5 and 7A. The BALF4 virus stock showed a fourfold increase in this assay, and another virus stock generated with BMRF1 showed a slightly improved fusogenic activity. Most other virus stocks did not show changes in their fusogenic activities. Within the members of the “low bin” group of genes (Fig. 8B, right panel), all virus stocks showed medium (BFRF1, BSLF1, and BTRF1) or much reduced fusogenic activities (BMRF2 to BMLF1), probably following a similarly reduced bioparticle concentration (Fig. 5) in this group of genes. Interestingly, while the fusogenicity of the BGLF4 virus stock was clearly compromised, the two virus stocks belonging to the “random” group, BPLF1 and BBRF1, had comparable fusion activity at the level of the control stock (Fig. 8B, right panel). The BBRF1 virus stock is an exception because its infectivity is reduced by more than 50% (Fig. 6), while its fusogenic activity is wild type.

Taken together, the assay demonstrates that BALF4 is the dominant driver of viral fusion with primary target B cells as established previously (11). In addition, the assay demonstrates that virus stocks generated by ectopic expression of certain viral genes, such as BBRF1, are not compromised with respect to virus fusion (Fig. 8B, right panel) but impaired in their infectivity (Fig. 7B). This observation illustrates that measuring virus fusion independent of virus infectivity is a valuable parameter in the herpesvirus field. BBRF1 is a prime example because it encodes the capsid portal protein (Table S1) indispensable for loading EBV DNA into viral capsids. Upon BBRF1 knockdown, the virus stocks probably contain fewer infectious virions (they lack the viral DNA genome), but the virus stocks nevertheless show the identical fusogenic activity compared with wild-type virus, indicating that fusion and *de novo* viral gene expression in EBV’s target cells are non-linked, independent steps in the multifaceted process of viral infection.

### Analysis of the fusogenic activity of 25 EBV stocks derived from lytically induced HH514 Burkitt lymphoma cells

All experiments conducted so far made use of the 2089 EBV producing cell line, which has been optimized to deliver very high titers of infectious EBV (6). The cellular identity of the parental HEK293 cells is uncertain, although the cell line originated from primary human embryonic kidney cells (30). Given this uncertainty, we searched for alternatives and tested several latently EBV-infected cell lines established from human tumor biopsies. Among them, we found the subline HH514 of the well-known P3HR1 Burkitt lymphoma cell line (8, 9), which tolerated transient transfection of plasmid DNA and released detectable amounts of progeny upon transient expression of BZLF1, confirming previous work (10). HH514 cells release infectious EBV, but virus titers are much lower compared with virus stocks from 2089 EBV producing HEK293 cells, which is an obstacle to analyze multiple parameters of high titer virus stocks as shown as far in this work.

To overcome this limitation, we employed a recently developed protein reporter system. It allows for the quantitative measurement of fusion events involving EBV and EBV-derived virus-like particles with exceptional sensitivity (31, 32). The protein reporter is based on Promega’s split nanoluciferase (33, 34), which comes in two parts: a truncated and thus inactive luciferase called LgBiT and a small, only 11-amino-acid-long 1.3 kDal part called HiBiT. Both parts can assemble spontaneously into a highly active luciferase entity. For this, we established Daudi cells, which express a fusion protein of CD63 and LgBiT as described (32). Daudi cells serve as highly sensitive target cells of infectious EBV (35). HH514 virus stocks were produced by transient co-transfection of expression plasmids encoding BZLF1, CD63:HiBiT, and 25 single EBV genes (or a negative control DNA) selected from the 3 groups (“high bin,” random, and “low bin”) as in Fig. 7 and 8.

**Fig 9:**
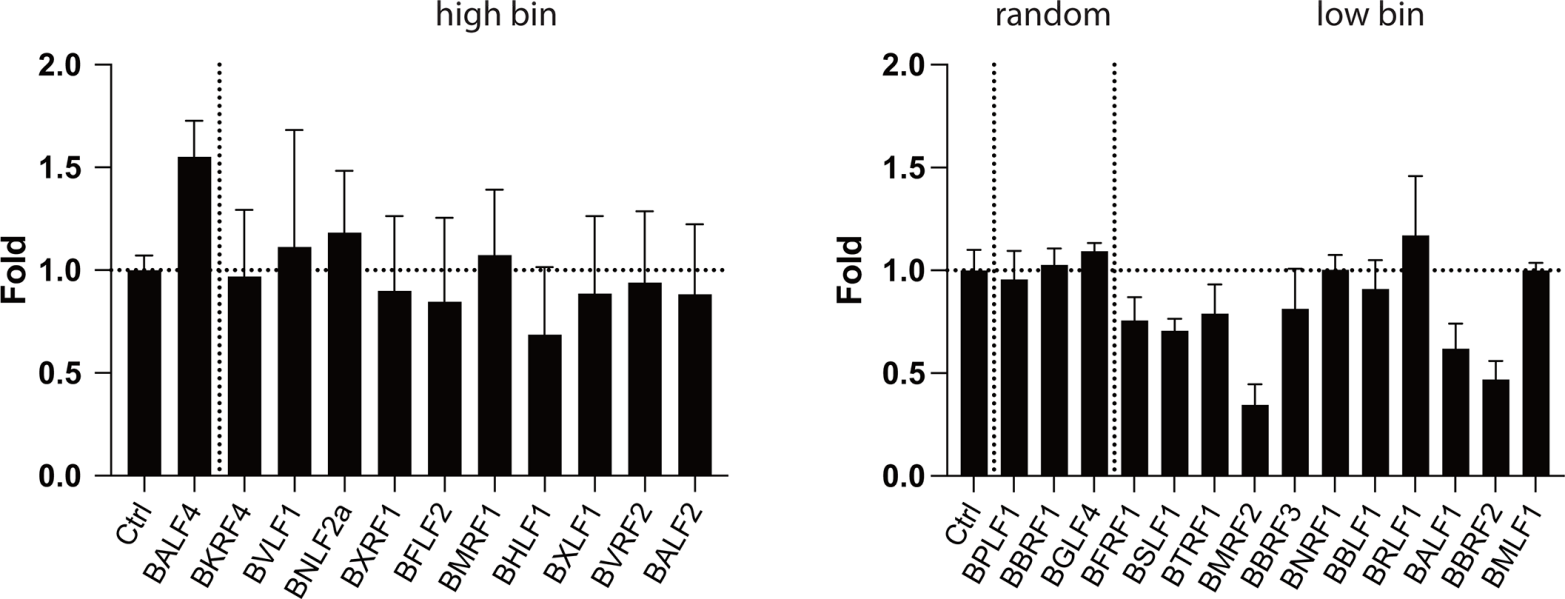
Engineered HH514 EBV stocks tested for their fusogenicity with Daudi cells. HH514 virus stocks were obtained from HH514 cells transiently transfected with 25 selected expression plasmids encoding individual viral genes. The cells were co-transfected with 3 plasmids encoding BZLF1 (p509), CD63:HiBiT (p7544), and 1 of the 25 selected genes or an empty control plasmid (pCMV). The harvested virus supernatants were added to CD63:LgBiT Daudi cells as recipients. In the figure, EBV genes were aligned on the *x*-axis and categorized in different groups, accordingly. The horizontal dotted line marks the baseline of the control. Vertical dotted lines separate different groups. The assay was performed by luminescence measurement and normalized.

Results from infection experiments with 25 individual HH514 stocks are shown in Fig. 9. The results can be directly compared with similar experiments using 25 individual 2089 EBV stocks with human primary B as target cells in Fig. 8B. Interestingly, HH514 cells appeared even more optimally adapted in producing EBV stocks compared to 2089 EBV producer cells. BALF4 enhanced the fusogenic activity of HH514 virus by a factor of 1.5 only, whereas the expression plasmids of the “low bin” group had no or a moderate dampening effect on this parameter, with the exception of BMRF2, BALF1, and BBRF2. A comparison of 2089 and HH514 virus stocks in this experimental setting indicated that HH514 stocks contain substantially less virus (as measured by the stocks’ fusogenic activities). The results also suggested that the effect of BALF4 differed when comparing HH514 and 2089 EBV stocks (Fig. S8), but an ectopic expression of BALF4 still provided an advantage in Daudi cells similar to primary human B cells (Fig. 8B).

## DISCUSSION

We have examined how both increasing and decreasing the levels of expression of viral genes modify the quality and quantity of EBV released from cells following the induction of its lytic cycle. Viruses pass through genetic bottlenecks when they are purified by plaquing. Infection by a herpesvirus that leads to latency is also potentially a genetic bottleneck because the genetic variation of the virus stock is reduced both by the limited number of cells initially infected and by the selection for the growth and survival of the infected cells. We expected that this genetic bottleneck would lead to the viruses maintained in latently infected cells being potentially less fit in their infectivity. Increasing and decreasing the levels of expression of viral genes during an induced escape from latency allow an assessment of any functional defects in the released virus.

In this study, a clone of HEK293 cells latently infected with a characterized derivative of the B95-8 strain of EBV was transfected with a vector expressing BZLF1 together with plasmids each encoding 77 individual EBV genes. Supernatants from these experiments were characterized for four properties of the released virus: (i) their physical particle concentration was measured with a nanoparticle tracking analysis instrument, (ii) the ability of the virus particles to bind solely to the CD21 receptor on a B-cell line was quantified, (iii) the ability of virus particles to fuse with or enter human primary B cells was directly analyzed in a newly developed functional assay, and (iv) the infectious titers of the released virus were assayed.

Multiple viral genes, when ectopically expressed, affected different biological functions. For example, BALF4, BDLF2, and BILF1 are all membrane proteins that, when increased in their expression, led to increased titers of infectious EBV. BALF4 encodes gB, a glycoprotein with homologs in all herpesviruses. BILF1 encodes a constitutively active G protein-coupled receptor presumably involved in signaling, whereas BDLF2, which is 1 out of at least 11 EBV glycoproteins, is a type II transmembrane protein with unknown functions (36). By contrast, the ectopic expression of certain viral genes, such as BALF1, decreased virus titers to as little as 10% of control values. BALF1 is involved both in regulating apoptosis and autophagy (37, 38). Obviously, EBV on escaping from latency expresses this protein at a level consistent with its optimal production of infectious progeny.

We used in parallel 75 shRNAs to target the transcripts of 25 viral genes selected to encompass the range of viral functions. The inhibition of a single gene, BFLF2, led to a modestly increased virus titer, as did its ectopic expression. BFLF2 interacts with BFRF1 to form a complex at the nuclear rim (39) so that its expression needs to be compatible with the level of its partner, BFRF1. The inhibition of several genes led to substantive changes in the number of infectious particles released. For example, decreasing the levels of expression of individual genes essential for the amplification of EBV DNA during its productive phase, including BMRF1, the processivity factor, and BALF2, the single-stranded DNA-binding protein, decreased the numbers of infectious particles released to less than 10% of control values.

Most data generated in this work stem from studying a single-cell clone, 2089 EBV producer cells (4). We learnt that this HEK293-based cell line releases substantially more infectious, immortalizing EBV in our hands than the standard EBV producer cell, the B95-8 strain (7) or the Akata strain of EBV (40). In an attempt to expand our approach to EBV strains other than B95-8 and to producer cells other than HEK293 cells, we tried very hard to establish conditions to transfect different established, latently infected EBV cell lines with expression plasmid DNA encoding BZLF1. To be useful, the cells must survive transfection and release sufficient (and reproducible) amounts of progeny to be compatible with readouts used throughout this work. Unfortunately, we failed in all but a single case. The majority of cell lines tested did not show any gp350 expression on the surface of BZLF1-transfected cells, which survived transfection (e.g., GG68 cells [8]). Other cell lines which release low levels of infectious EBV consistently (such as a panel of lymphoblastoid cell clones established by infecting primary B cells with different EBV strains including M81) did not respond with an increase of gp350 surface expression upon ectopic expression of BZLF1. This was an unexpected finding, which could be attributed to the protein level of BZLF1 needed to break latency of EBV (41).

The HH514 cell line, a subclone of the parental P3HR1 Burkitt lymphoma cell line (8, 9), was the only cell line that released sufficient amounts of EBV upon transient expression of BZLF1, which is consistent with our previous findings (10). Even in the case of HH514 cells, we had to adapt our standard BlaM fusion assay to a much more sensitive split nanoluciferase approach (31, 32), which uses Daudi cells (35, 42) as EBV’s B-cell recipient. In this fusion assay, we found that HH514 cells seem to be even more robust compared with 2089 EBV producer cells in generating progeny (compare Fig. 8B and 9). BALF4 expression led to an enhanced fusogenic activity of HH514 virus by a factor of 1.5, only, whereas expression plasmids of the “low bin” group had no or only a moderate dampening effect with the exception of BMRF2, BALF1, and BBRF2. Readouts such as physical particles, bioparticles, or virus titers were not practical as HH514 virus lacks a suitable GFP marker protein and HH514 stocks did not contain sufficient amounts of virus.

**Fig 10:**
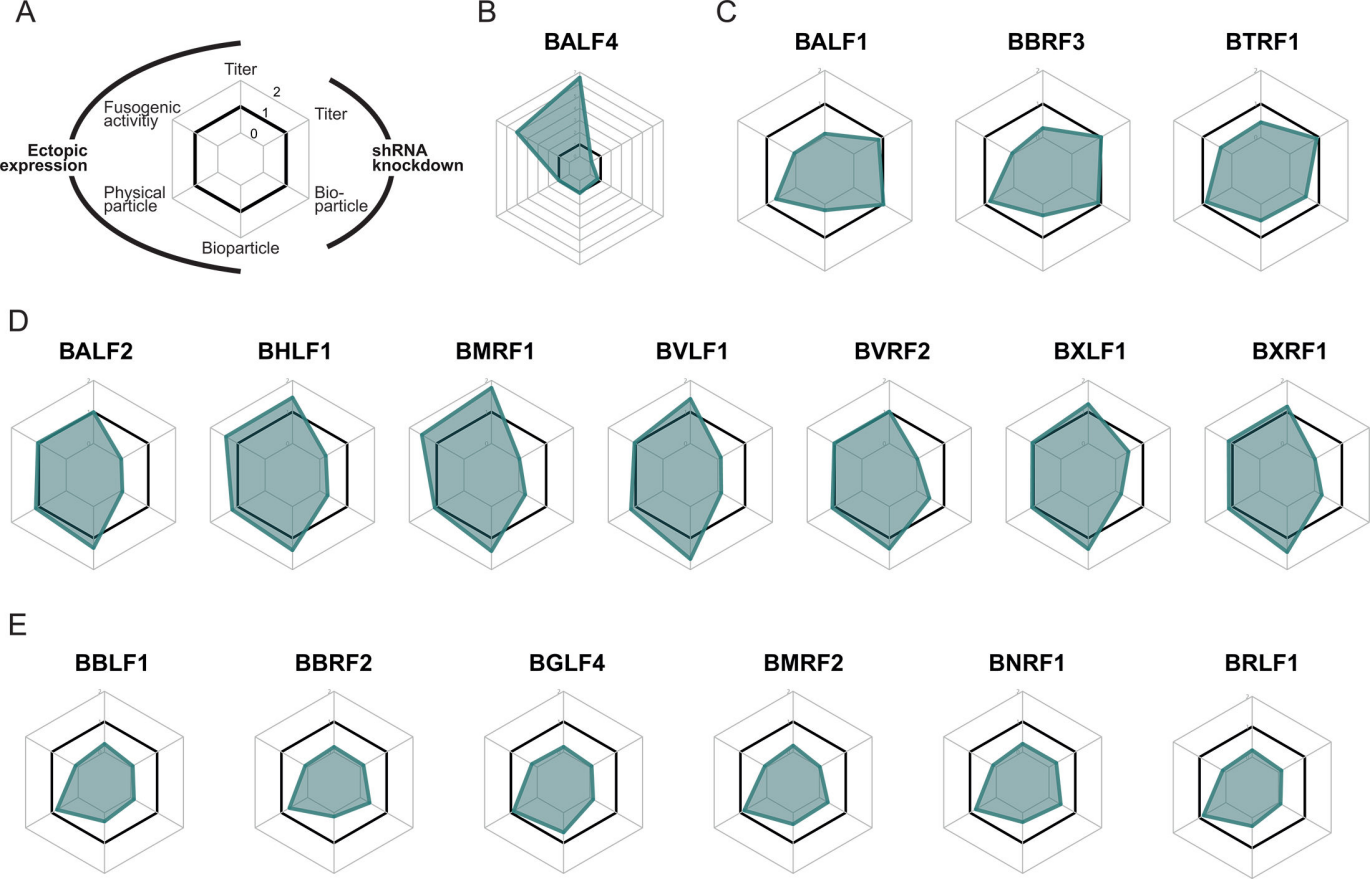
Spider charts visualizing the results of six parameters derived from the analysis of 17 selected viral genes. (**A**) Schematic view of the spider charts. Four of the six “legs” of the diagram represent the calculated ratios of readouts encompassing “physical particle,” “bioparticle,” virus “titer,” and “fusogenic activity” upon ectopic expression of selected viral genes. The remaining two legs show the consequences of shRNA-mediated knockdown of a given gene affecting virus “titer” and “bioparticle”. Concentric hexameric rings indicate ratios of 1 (no change of the parameter), 0 (complete functional loss), and 2 (twofold increase of the parameter). (**B**) The six ratios obtained with BALF4. Ectopic expression of BALF4 in 2089 EBV producer cells increases the virus titer and the fusogenic activity about seven- and fourfold, respectively. The shRNA-mediated knockdown of BALF4 leads to an almost complete loss of the parameter virus “titer,”’ indicating that BALF4 is indispensable for virus synthesis. (**C**) Three viral genes, which, when expressed ectopically, show reduced ratios of “bioparticles,” virus “titer,” and “fusogenic activity.” Their shRNA-mediated knockdown does not affect “bioparticles” and virus “titer” ratios. (**D**) The selected seven viral genes have little effect on four parameters when expressed ectopically, but their shRNA-mediated knockdown severely compromised the parameter ratios “bioparticle” and virus “titer.” (**E**) The parameter ratios of “bioparticle,” virus “titer,” and “fusogenic activity” of six viral genes are compromised upon their ectopic expression**,** and their shRNA-mediated knockdown profoundly affects the parameters “bioparticle” and virus “titer” as well. Fig. S9 provides additional spider charts of other viral genes analyzed.

We searched for a means to display our complex and potentially dependent measurements and have used spider charts to show the data from 2089 producer cells with multiple variables in the form of two-dimensional images. We have found three functional classes as shown in panels C, D, and E of Fig. 10. BALF1, BBRF3, and BTRF1 appear to be non-essential but, when ectopically expressed, compromise bioparticle release, virus titers, and the fusogenic activity of virus stocks. In contrast, genes such as BHLF1, BMRF1, and BVLF1, when inhibited, greatly reduce virus yields but improve virus titers and increase bioparticle concentrations only marginally upon ectopic expression. Finally, six genes, such as BBLF1 and BBRF2, when inhibited, greatly reduce virus titers, but their forced expression is detrimental, indicating that they must be particularly tightly regulated to support optimal virus production. Our data corroborate the recently documented important role of BBLF1 in virus production (43) (Fig. 10E), but our results diverge with respect to two other viral genes, BKRF4 and BFLF2 (44, 45). Both viral genes have been reported to play important roles in virus production, but our knockdown data did not confirm these findings (Fig. S9A and C).

One unexpected and fascinating outcome of all these experimental perturbations of gene expression is that their natural levels on entry into the lytic phase are close to optimal for multiple facets of virus production. Except for BALF4, no increased expression increases fusion with host cells by more than 50%, none increases particle formation by twofold, and none increases viral titers by twofold, either. Consistent with the levels of viral genes being optimal for virus production, the inhibition of multiple viral genes led to significant decreases in this production. EBV thus maintains the fitness of infectivity of its released virus while being propagated latently for many generations.

What properties of EBV’s latent infection contribute to preserving the robustness of the virus released on its rare escape from a latent infection? We speculate that at least two are important. First, mutations in viral genes will occur approximately 1% as frequently when DNA synthesis is mediated by the host cell as when it is by the virus. In cells, the rate of mutation within one gene is on the order of 3 × 10^−7^per generation, while for HSV-1 it is about 3 × 10^−5^ per round of replication (46, 47). EBV maintains its genomes as multiple, extra-chromosomal plasmids in each cell. Mutations arising in one copy in one cell are unlikely to occur in another copy in that cell. When that cell enters the lytic phase, its wild-type alleles will usually compensate for the individual, mutant form. Second, epigenetic mechanisms will modify viral gene expression. EBV DNA is progressively methylated as its host cell proliferates (48, 49). This modification has two opposing effects: it can inhibit expression of some viral genes such as the viral miRNAs, but it is essential for the binding of BZLF1 necessary for EBV’s productive cycle (50, 51). EBV sheds epigenetic marks during its lytic phase so that only unmodified DNA is in the virus particles that infect naïve cells. This wild-type genetic complement ensures the fitness of the progeny virus to express its genes optimally to mediate a new round of transformation of the infected host cell.

## MATERIALS AND METHODS

### Cell culture and cell lines

RPMI1640 medium supplemented with 10% FBS, 100 U/mL penicillin-streptomycin, 1 mM sodium pyruvate, 100 pM sodium selenite, and 0.04% α-thioglycerol was used to culture all cells. 2089 carrying HEK293 EBV producer cells were cultivated in supplemented RPMI1640 medium containing 100 µg/mL hygromycin B. EBV producer cells transduced with shRNA-encoding lentiviral vectors were cultivated in supplemented RPMI cell culture medium and were kept in 100 µg/mL hygromycin B and 3 µg/mL puromycin after initial selection with 10 µg/mL puromycin for 7 days. HH514 cells were cultivated in RPMI1640 medium supplemented with 10% FBS but without antibiotics. CD63:LgBiT Daudi cells were cultivated in supplemented RPMI1640 medium containing 200 ng/mL puromycin. All cells were incubated in a 5% CO_2_ and water-saturated atmosphere at 37°C.

### Transient transfection of 2089 EBV producer cells

2089 EBV producer cells (6.5 × 10^5^) (4) were seeded in 6-well cluster plates. After overnight incubation, the cell medium was exchanged with 2 mL EV-depleted (Ex^–^) medium (Fig. 2). Furthermore, 0.5 µg BZLF1 and 0.5 µg expression plasmid DNAs were mixed in a vial with 100 µL plain RPMI1640. In another vial, 100 µL plain RPMI1640 was mixed with 6 µL PEI MAX (6 µL PEI MAX per 1 µg plasmid DNA). The content of both vials was combined, rigorously mixed, and the mixture was incubated at room temperature for 20 min and was added to the EBV producer cells in a single well of a 6-well cluster plate.

### Transient transfection of Burkitt lymphoma HH514 cells

HH514 cells (5 × 10^6^) were harvested and washed with PBS. The cells were resuspended in 250 µL 10% FBS RPMI culture medium and transferred to 0.4 cm cuvettes (Bio-Rad). Furthermore, 5 µg BZLF1, 5 µg CD63:HiBiT, and 5 µg EBV expression plasmid DNAs were added to the cells in suspension. A Bio-Rad Gene Pulser II Electroporation System was used to electroporate HH514 cells with parameters set to 220 V and 975 µF. The electroporated cells were cultivated in 2 mL RPMI culture medium. Three days after electroporation, the supernatants were harvested and stored at 4°C prior to analysis.

### Assembly of ribonucleoprotein complexes

Synthetic sgRNAs (Synthego) were dissolved in nuclease-free Tris-EDTA buffer (1× TE buffer) (Synthego) at a concentration of 100 µM. Also, 6.5 µL of a 62 µM Cas9 V3 protein preparation (1081059; Integrated DNA Technologies) was added to 10 µL of the 100 µM gRNA solution. The mixture was diluted with sterile-filtered (0.22 µm) PBS to a final volume of 50 µL and incubated at room temperature for 10 min to allow formation of RNPs at a final concentration of 8 µM Cas9. The two gRNAs specific for the *CR2* target gene were designed using the Synthego website. Table S4 provides the two gRNA sequences and their target nucleotide coordinates in exon 2 and exon 3 of the *CR2* locus on chromosome 1.

### Nucleofection of RNP complexes into Elijah cells

Elijah cells (2 × 10^6^) were washed in PBS and resuspended in 20 µL P3 Primary Cell Nucleofector Solution buffer prepared with supplement 1 buffer (Lonza) according to the manufacturer’s instructions (P3 Primary Cell 4D-Nucleofector X Kit S). For each of the two assembled gRNA-Cas9 complexes, 2.5 µL was mixed with the cell suspension, which was transferred to a pre-cooled (4°C) well in a 16-well Nucleocuvette Strip (Lonza). Cells were nucleofected using the EH-100 program of Lonza’s protocol. Furthermore, 100 µL pre-warmed (w/o supplements) RPMI1640 medium was added to the cells, which were incubated for 15 min at 37°C. The cells were transferred to a single well of a 24-well cluster plate, and complete pre-warmed cell culture medium containing 20% FBS was added to a final volume of 220 µL to allow cell recovery. The cells were incubated at 37°C, 5% CO_2_.

### Flow cytometry and cell sorting

Elijah cells (1 × 10^5^) were washed with and resuspended in 50 µL of FACS staining buffer (PBS, 1% FBS, 2 mM EDTA) and stained with 1 µL of an APC-conjugated CD21-specific antibody (clone: HB5; 130-101-739, Miltenyi Biotec). The cells were incubated at 4°C in the dark for 20 min and washed with and resuspended in 200 µL FACS staining buffer. Flow cytometry data were collected on a FACSCanto instrument (Becton Dickinson). For sorting, Elijah cells were stained with the CD21-APC antibody as described above. Cells were washed with and resuspended in the FACS staining buffer and filtered through a 100-µm mesh cell strainer to obtain single-cell suspensions. Sorting was performed with a 100-µm-wide nozzle, a velocity of about 8,000 events/second, and a sorting mask of “four-way purity” using a BD FACS Aria IIIu instrument. The gating criteria included (i) living cells, (ii) single cells, and (iii) CD21-negative cells. Data analysis was performed using the FlowJo software (version 10.4).

### shRNA expression vector construction and sequence design of shRNAs

The DNA sequences of selected EBV genes were obtained from the database of National Center for Biotechnology Information (NCBI). The FASTA format sequences were used for feeding the splashRNA tool (http://splashrna.mskcc.org) to predict potent shRNA sequences. According to the splashRNA score, the top three antisense guide sequences were chosen, ordered as synthetic oligonucleotides, and inserted into the miR-3G frame of the basic shRNA construct (p6924 in our plasmid database). For each chosen EBV gene, a knockdown pool of three shRNA constructs was designed and realized. The antisense guide sequences of individual EBV genes are marked in the entries shown in Table S2, which have compatible ends to be cloned into AvrII and EcoRI restriction sites in the basic shRNA construct p6924. The shRNA constructs contain the puromycin resistance gene as a selection marker.

### Virus titer measurement (infectivity)

The virus stocks were generated by transient DNA transfection of the 2089 EBV producer cells, and the supernatants, harvested 3 days after transfection, were subsequently tested for infectious virus. Virus stocks were added to 1 × 10^5^ Raji cells in a volume of 2 mL cell culture medium and incubated for 3 days. The infected cells which express green fluorescence protein were determined, and the fraction of GFP-positive cells, termed green Raji unit, was quantified by flow cytometry (BD FACSCanto).

### Bioparticle quantification (Elijah cell binding assay)

Elijah cells (2 × 10^5^ in 1 mL) were incubated with the 20 µL harvested virus stocks. The mix was agitated on a mixing roller at 4°C for 3 h. The cells were pelleted at 500 g at 4°C for 10 min and washed with 1 mL ice-cold staining buffer (1% FBS and 2 mM EDTA in PBS). The anti-gp350 antibody (6G4) (41) coupled to Alexa647 in 50 µL staining buffer (1:250 dilution) was added, and the Elijah cells were incubated at 4°C for 20 min. 1 mL staining buffer was added, and the cells were washed and resuspended in 300 µL staining buffer and analyzed by flow cytometry (BD FACSCanto). The Elijah cells were analyzed for their fluorescence in the appropriate channel to obtain mean fluorescence intensity data. MFI is an indirect measure of bound virus and correlates with the number of viral particles attached to the surface of Elijah cells.

### Physical particle measurement (nanoparticle tracking analysis)

The physical particle concentration was measured using the ZetaView PMX110 instrument (Particle Metrix), which can perform nanoparticle tracking analysis. Harvested virus stocks were diluted with PBS to adjust the concentration of particles to about 10^7^–10^8^ particles per milliliter. Standard calibration beads (102.7 ± 1.3 nm) (Polysciences) were used to confirm the range of linearity of the instrument. Also, 1 mL diluted supernatant samples were injected for analysis. All samples in the chamber were recorded at 11 positions in 3 cycles. Pre-acquisition parameters were set to 75 sensitivity, 70 shutter speed, a frame rate of 30 frames per second, and 15 trace length. The post-acquisition parameters were set to a minimum brightness of 20, a minimum size of 5 pixels, and a maximum size of 1,000 pixels. Particle concentration and particle size were measured and documented, and the images were analyzed using the ZetaView 8.04.02 software.

### Luciferase reporter construction

Three corresponding target/sense sequences were inserted into the dual-luciferase reporter plasmid psiCHECK2 (p5264). The sequences were inserted downstream of the Renilla luciferase coding sequence using the restriction enzyme sites XhoI and NotI. Firefly luciferase is used as an internal control. The shRNA target sequences are shown in Table S3.

### Luciferase assay

293T cells (2 × 10^5^) were seeded in a 24-well cluster plate. After overnight incubation, cells were co-transfected with 100 ng psiCHECK2 luciferase reporter plasmid and 300 ng individual corresponding pCDH shRNA expression plasmid DNA. An empty pCDH shRNA expression plasmid (p6924) was transfected as control. Cells were lysed after 24 h, substrate was added and the luciferase activity was measured using the Orion luminometer (Berthold). To detect the knockdown efficiency of stably shRNA-transduced 2089 EBV producer cells, 1 × 10^5^ producer cells were seeded in a 24-well cluster plate. After overnight incubation, cells were transfected with 100 ng corresponding psiCHECK2 luciferase reporter plasmid. An empty psiCHECK2 luciferase (p5264) reporter plasmid was transfected as control. After 24 h, cells were lysed, substrate was added and the luciferase activity was measured using the Orion luminometer.

### β-Lactamase (BlaM) fusion assay

EBV particles equipped with CD63:BlaM were generated by co-transfecting 6.5 × 10^5^ 2089 producer cells with expression plasmids encoding CD63:BlaM (p7200, 0.25 µg) and BZLF1 (p509, 0.25 µg), together with 0.5 µg plasmid DNA encoding single EBV genes to generate virus supernatants for further testing. The open reading frame of human CD63 is C-terminally fused to a codon-optimized β-lactamase via a G_4_S linker and cloned into the expression plasmid pcDNA3.1 (+) (p5267). To evaluate the fusogenic activities of different virus stocks, 2 × 10^5^ human primary B cells were incubated with 5 µL virus supernatants for 4 h. Cells were spun down and re-suspended in 100 µL of CCF4-AM staining solution in a 96-well plate. The staining solution consisted of 2 µL CCF4-AM (membrane-permeant ester forms of the negatively charged fluorescent β-lactamase substrate), 8 µL Solution B (K1095, Thermo Fisher Scientific), and 10 µL of 250 mM Probenecid (P8761, Sigma) in 1 mL CO_2_-independent medium (18045-054, Thermo Fisher Scientific). After 16 h incubation in the dark at room temperature, the BlaM-positive recipient cells were analyzed by flow cytometry (BD LSRFortessa). The CCF4 FRET substrate is excited with a 409-nm wavelength laser (violet). The non-cleaved substrate emits light with a 520 nm wavelength (green), while the cleaved substrate emits light with a wavelength of 447 nm (blue).

### Virus fusion with the split nanoluciferase assay

CD63:LgBiT constitutively expressing Daudi cells, a Burkitt lymphoma cell line (42), were generated via retroviral transduction using an appropriately designed vector followed by selection with puromycin as described (32). 4 × 10^4^ CD63:LgBiT expressing Daudi cells in 50 µL cell culture medium were added to single wells in a 96-well Lumitrac200 (655076, Greiner Bio-One) plate. Furthermore, 150 µL of the HH514-derived virus stock was added to the recipient cells, which were incubated at 37°C for 24 h. The cells were pelleted by centrifugation, and the supernatants were removed by pipetting. Also, 25 µL of the substrate mix (20 µL OptiMEM, Gibco, Thermo Fisher Scientific, 5 µL nano-Glo 1:20 diluted in LCS buffer, N2012, Promega) was added to the cells, and their luminescence was measured immediately using a CLARIOstar Plus reader (BMG Labtech).

### Isolation and preparation of human primary B cells from adenoids

Human primary B cells were purified from adenoidal tissues, which were chopped with blades and washed with PBS. The mashed tissues were filtered with a 100-µm sterile strainer (352360, Falcon), and the cells were transferred to a sterile 50 mL tube. The volume was increased with PBS to 30 mL, and 1 mL defibrinated sheep blood (SR0051D, Thermo Fisher Scientific) was added and mixed (to sediment T cells in the next step). The cell suspension was slowly layered on top of 15 mL Pancoll human (density: 1.077 g/mL) (P04-60500, PAN-Biotech) in a 50 mL tube. Samples were centrifuged at 1,900 rpm at room temperature for 30 min without brake. The interphase (white band) was collected and transferred to a new tube. The cells were washed three times with PBS and centrifuged at different speeds of 1,500, 1,400, and 1,200 rpm for 10 min.

### Physical particle concentration of formulated and conditioned cell culture media

Concentrations of physical particles in formulated and conditioned cell culture media were measured using a nanoparticle tracking analysis instrument. Different formulated media and samples of cell culture media were analyzed. “RPMI^–^” was plain RPMI1640 medium purchased from the manufacturer; “RPMI^+^” was plain RPMI1640 medium with all supplements for cell culture, excluding fetal bovine serum; “Ex^–^ medium” was prepared by sedimenting 35 mL of “RPMI^+^” medium supplemented with 20% FBS by ultracentrifugation in a SW32 or SW28 swing-out rotor (Beckman) at 4°C for at least 16 h. Furthermore, 30 mL of the supernatant was removed and diluted with an equal amount of “RPMI^+^” medium. The medium was filtered with a 0.22 micron filter to yield 10% FBS “Ex^–^ medium.” To quantify particle numbers in conditioned cell medium, 2 mL of “Ex^–^ medium” was incubated with non-induced or induced producer cells in 6-well cluster plates as shown in Fig. 2 for 3 days later when the medium was collected, filtered using a 1.2-micron syringe filter, and analyzed.

### LIVE/DEAD cells staining

2089 EBV producer cells (6.5 × 10^5^) were seeded in 6-well cluster plates. The next day, the cells were transfected with 0.5 µg expression plasmid DNA. Three days after transfection, the cells were trypsinized, washed with PBS, and were resuspended in 1 mL PBS. Furthermore, 1 µL of the reconstituted fluorescent dye (LIVE/DEAD Fixable Far Red Dead Cell Stain Kit, ThermoFisher) was added and mixed well. The mixture was incubated at 4°C for 30 min. The cells were further washed and quantified by flow cytometry (BD FACSCanto).

### Statistical analysis

Statistical analyses of individual grouped data encompassing the results of physical particles (Fig. 3; Fig. S1), bioparticles (Fig. 5; Fig. S2), and virus titers (Fig. 6; Fig. S3) were performed using R (version 4.0.5). The first tests for equality of variances between the groups were done with an *F*-test. Based on the outcome of this variance test, either Student’s *t*-test (for equal variances) or Welch’s *t*-test (for unequal variances) was applied for pairwise comparisons between the reference group and all other experimental groups. The significance threshold was set at α = 0.05, with *P*-values below this threshold considered statistically significant. Results were compiled into summary tables containing test statistics (*t*-values, degrees of freedom), *P*-values, and significance determinations for each comparison. Table S5 contains the three compiled summary tables with all data.

### Software tools

MacVector version 18.0.0 was used for *in silico* DNA cloning. FlowJo 10.4.2 was used for analysis and visualization of flow cytometry data. The ZetaView 8.04.02 software was used for nanoparticle tracking analysis. Prism 9 for macOS version 9.0.1 (128) and R version 4.0.5 with ggplot2 version 3.3.3 and fmsb version 0.7.0 packages were used for statistical analysis and visualization. Microsoft Word for Mac version 16.45 and Microsoft Excel for Mac version 16.45 were used for documentation and analysis. Adobe Illustrator 25.1 was used for composing figures. The diagrams were created with BioRender.com (https://biorender.com/).

## Data Availability

All relevant raw data will be freely available to any scientist wishing to use them for non-commercial purposes. Materials described in the article will be available to any scientist after signing a legally binding Material Transfer Agreement between his/her/their institution and Helmholtz Munich.

## SUPPLEMENTAL MATERIAL

Supplemental figures jvi.00626-25-s0001.pdf Figures S1 to S9.

Supplemental legends jvi.00626-25-s0002.docx Legends for Fig. S1 to S9.

Table S1 jvi.00626-25-s0003.docx Overview of identity, related function, and expression timing of 78 EBV genes.

Table S2 jvi.00626-25-s0004.docx Overview of synthetic oligonucleotides cloned into the basic lentiviral shRNA expression vector plasmid p6924 to be stably introduced into 2089 EBV producer cells.

Table S3 jvi.00626-25-s0005.docx Synthetic pairs of oligonucleotides used for the construction of luciferase reporter plasmids.

Table S4 jvi.00626-25-s0006.xlsx gRNA sequences.

Table S5 jvi.00626-25-s0007.xlsx Statistical analysis.

ASM does not own the copyrights to Supplemental Material that may be linked to, or accessed through, an article. The authors have granted ASM a non-exclusive, world-wide license to publish the Supplemental Material files. Please contact the corresponding author directly for reuse.
