# Supplementary material for: Assessing the fitness of Epstein-Barr virus following its reactivation
Source: J Virol. 2025 May 30;99(7):e00626-25. doi: 10.1128/jvi.00626-25 (PMC12282151; doi:10.1128/jvi.00626-25)
Supplement: Supplemental figures — Figures S1 to S9. [file jvi.00626-25-s0001.pdf]

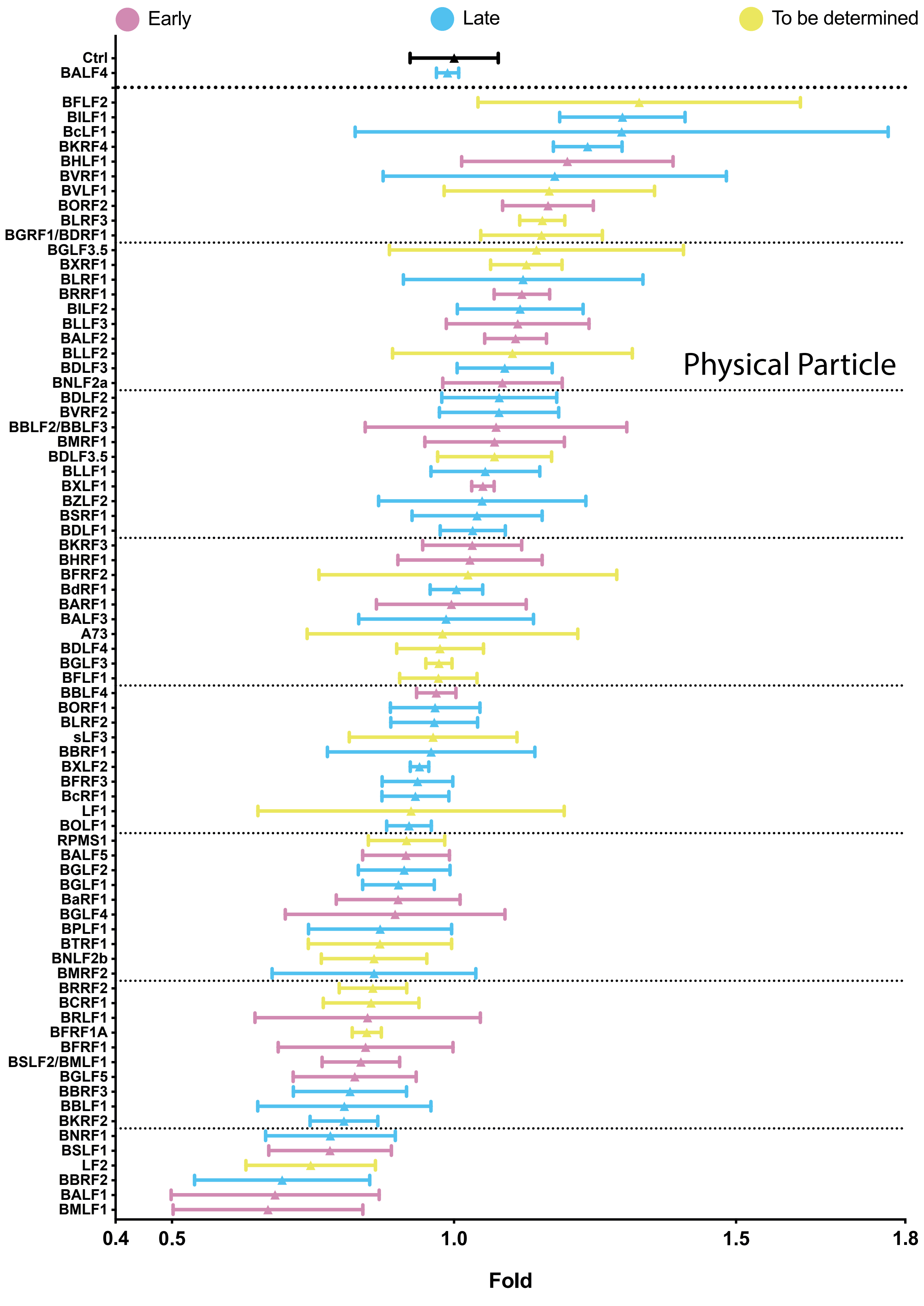

Supplementary Figure S1

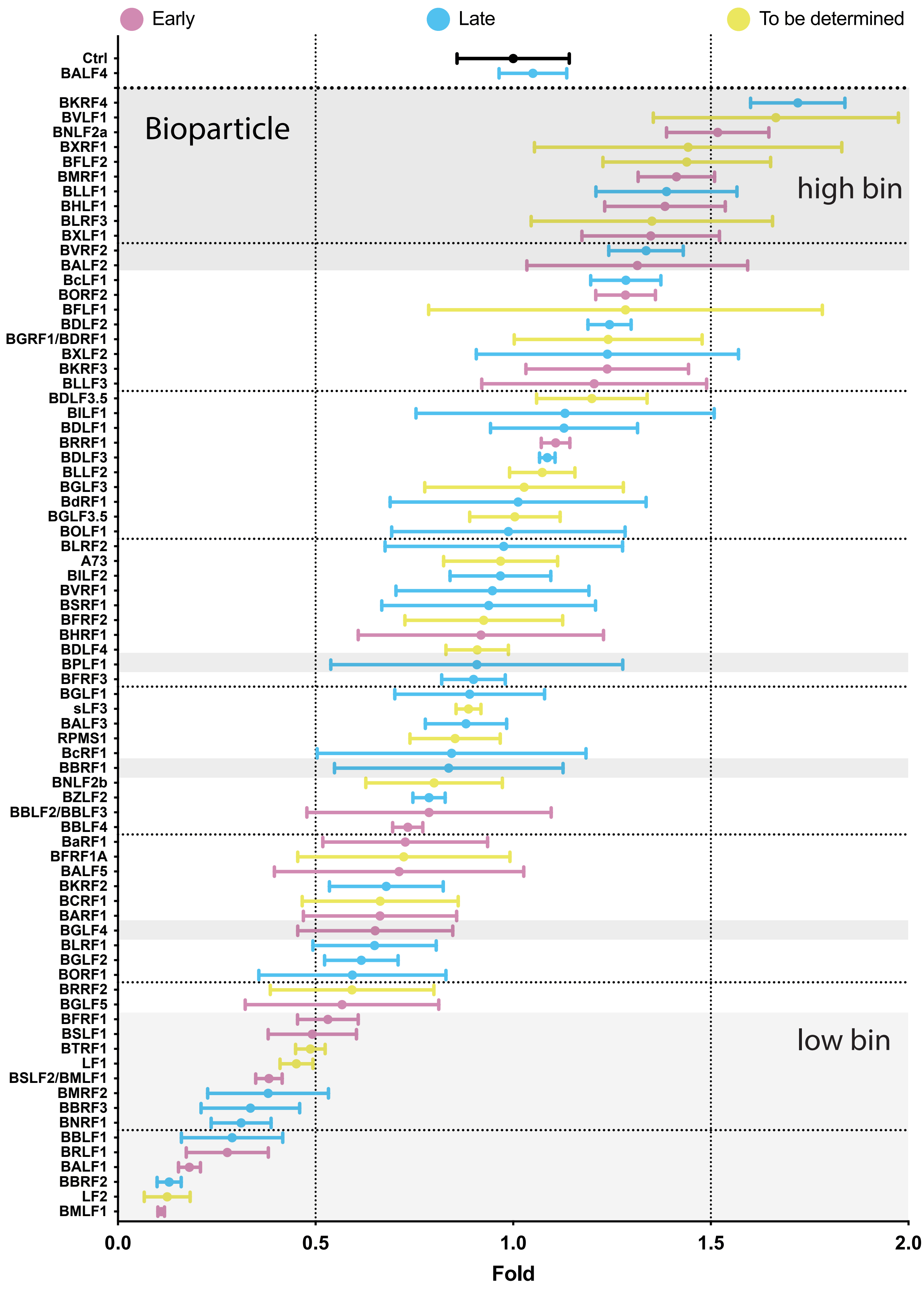

Supplementary Figure S2

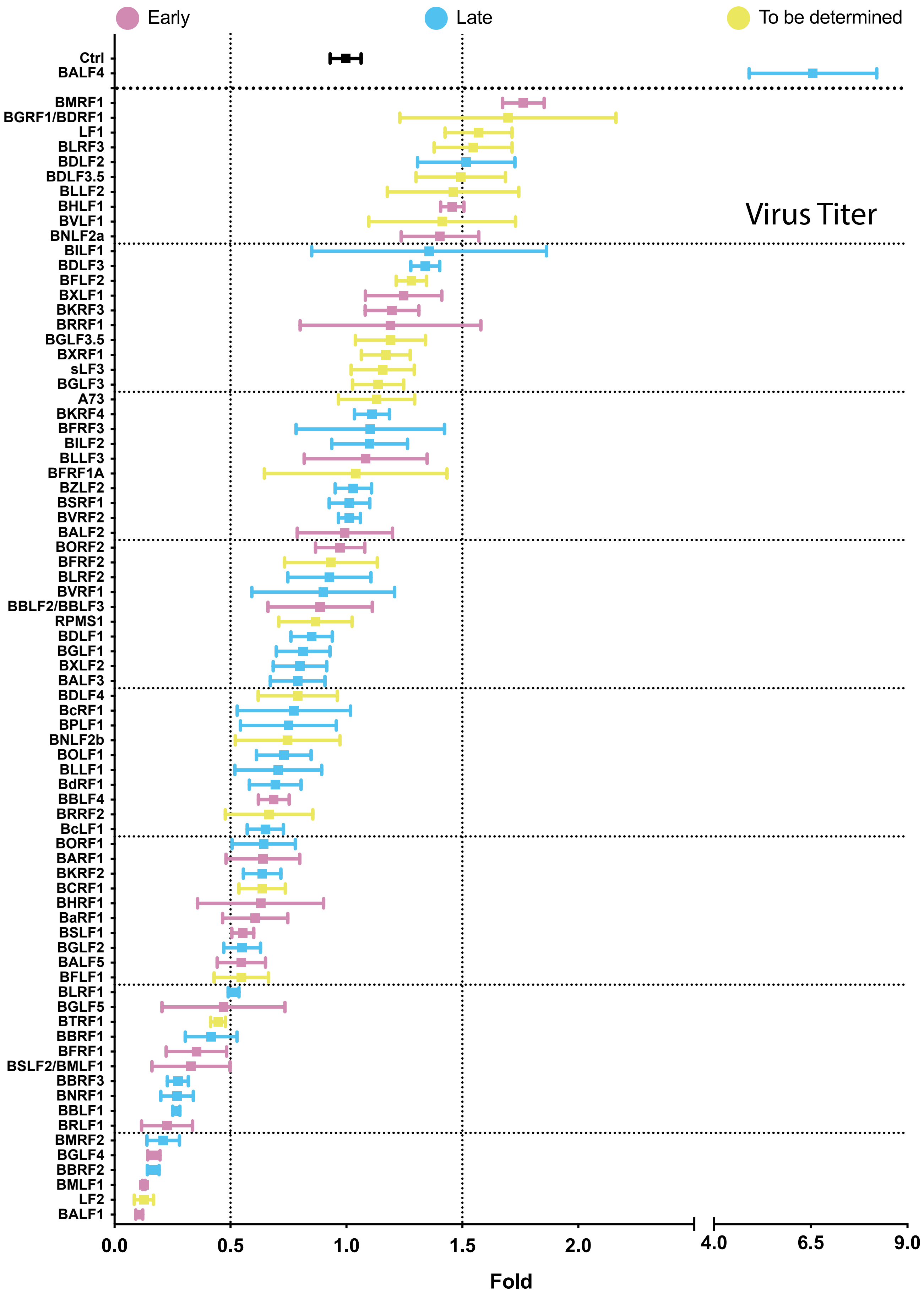

Supplementary Figure S3

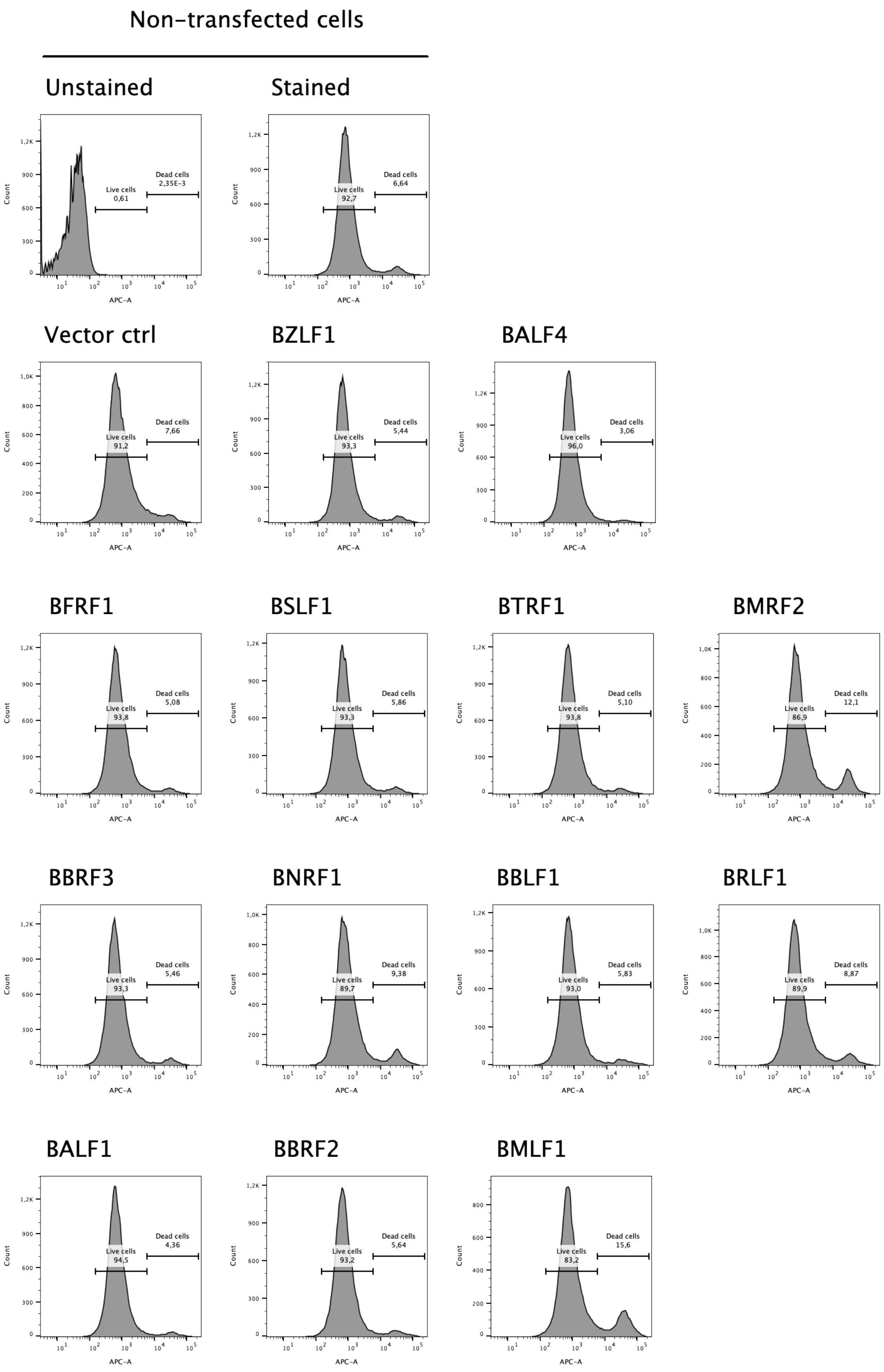

Supplementary Figure S4

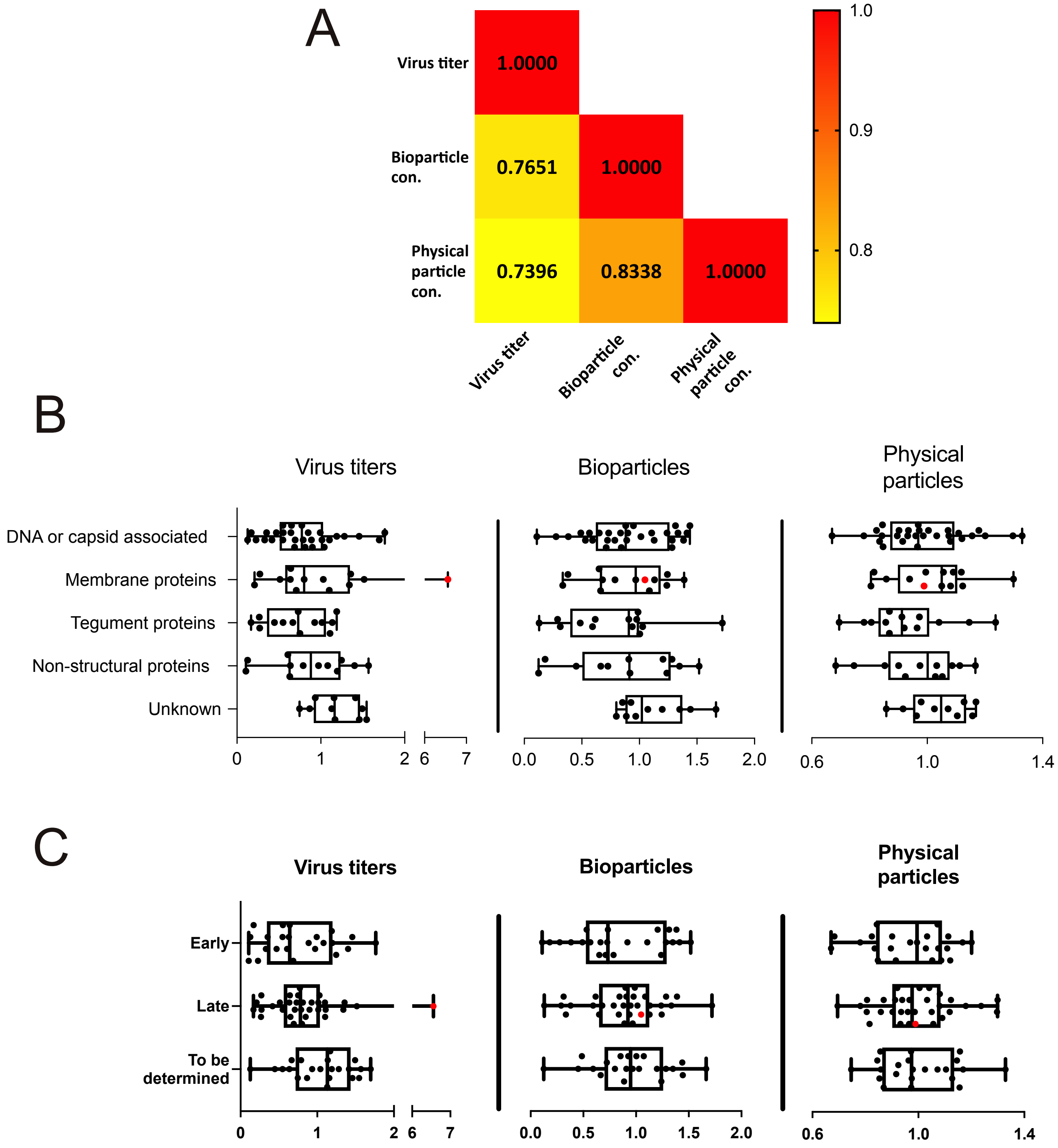

Supplementary Figure S5

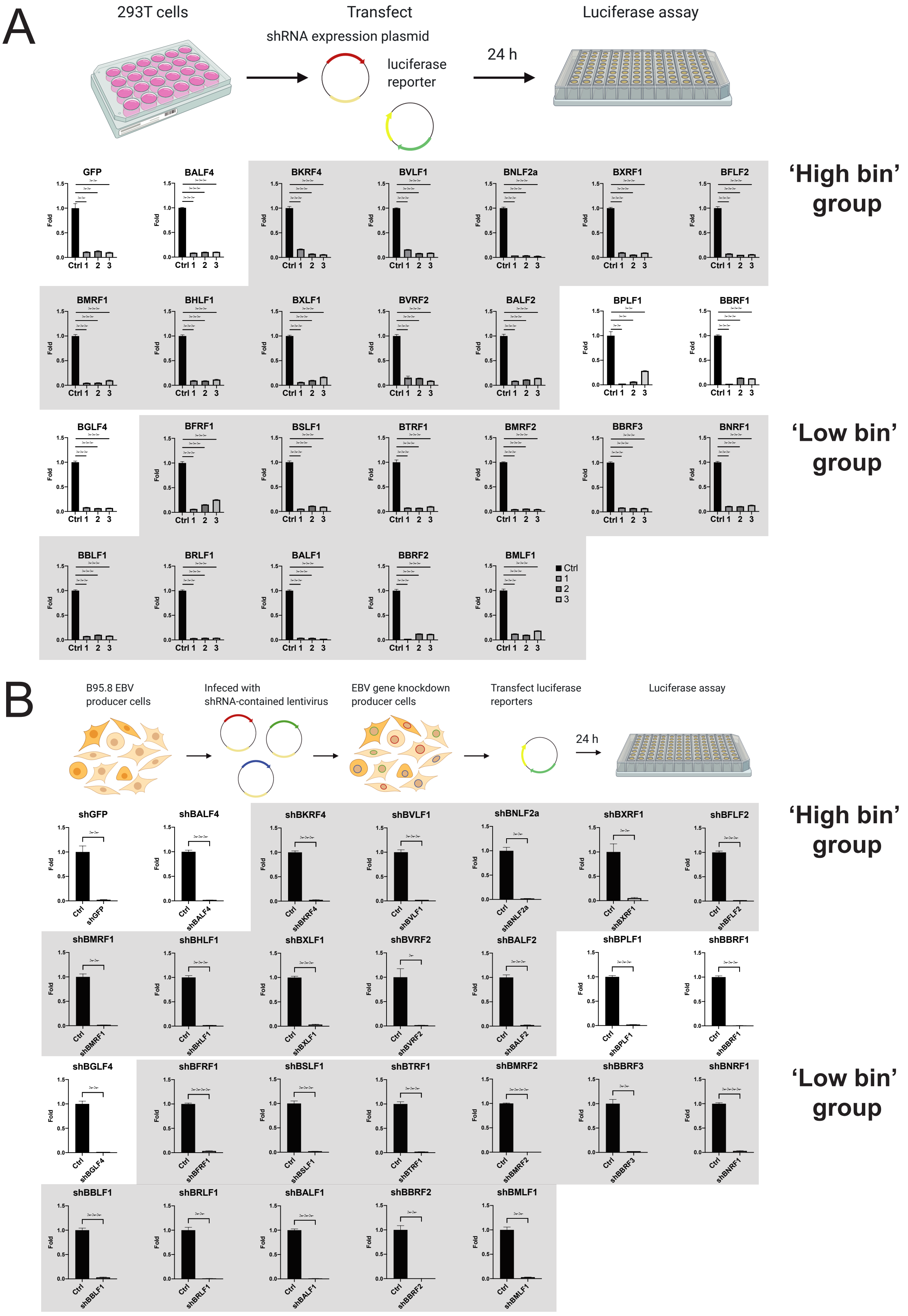

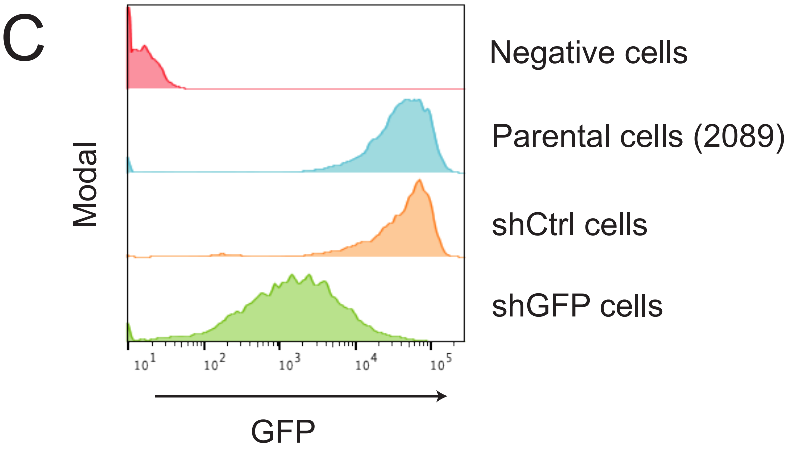

Supplementary Figure S6

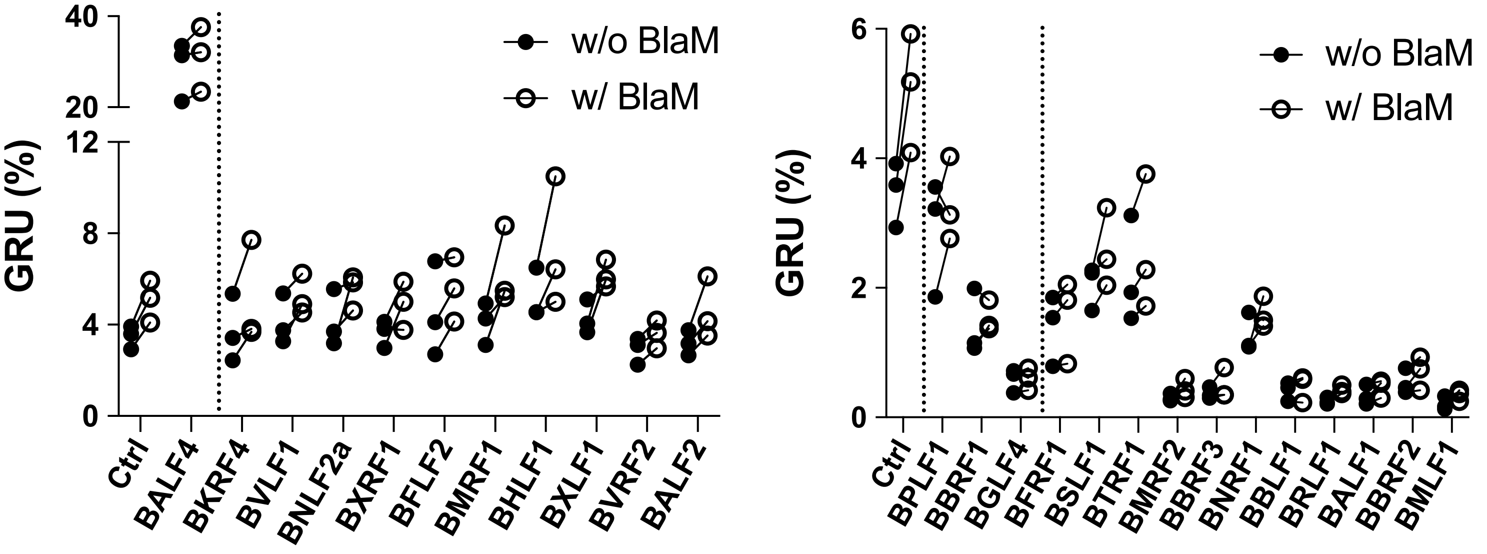

Supplementary Figure S7

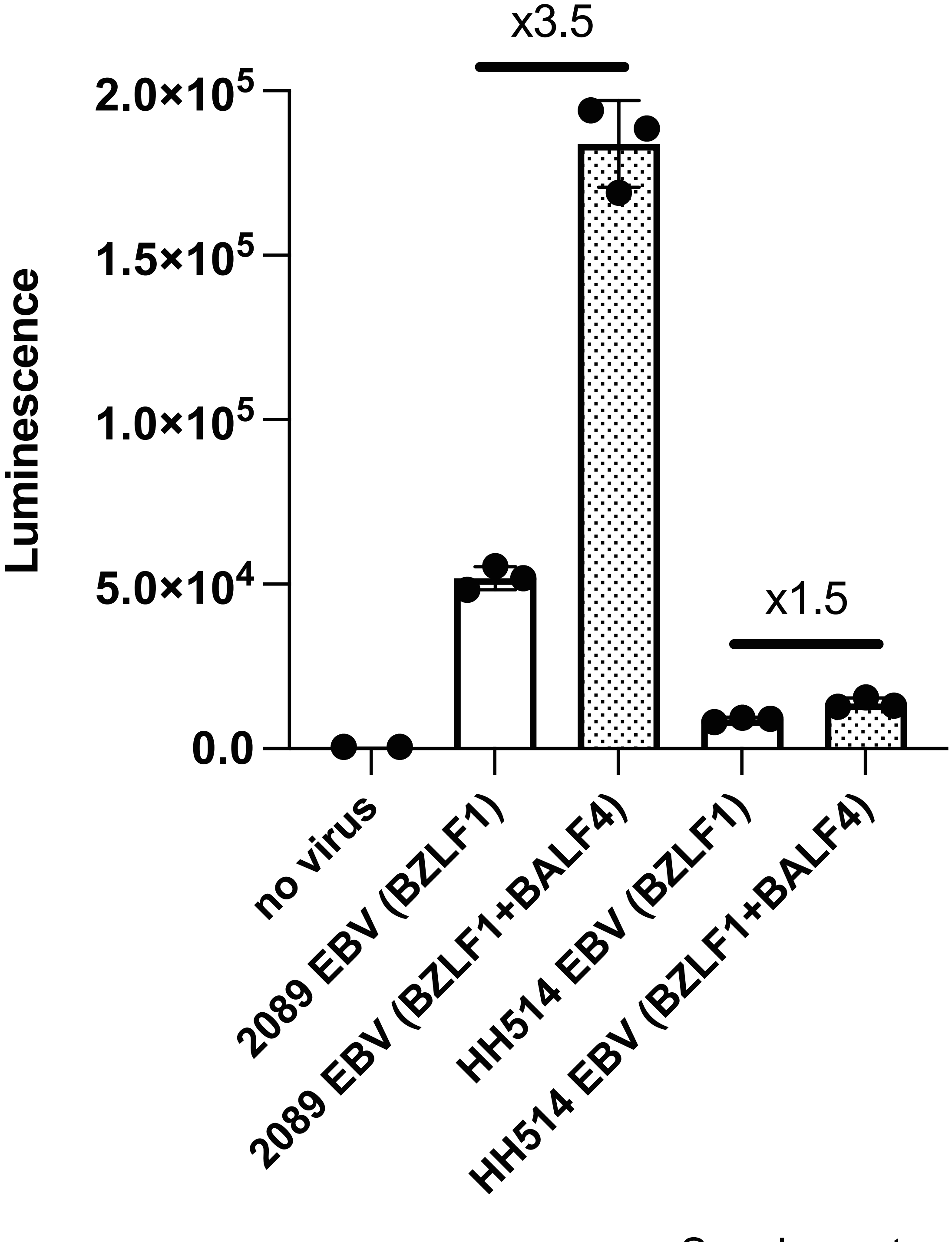

Supplementary Figure S8

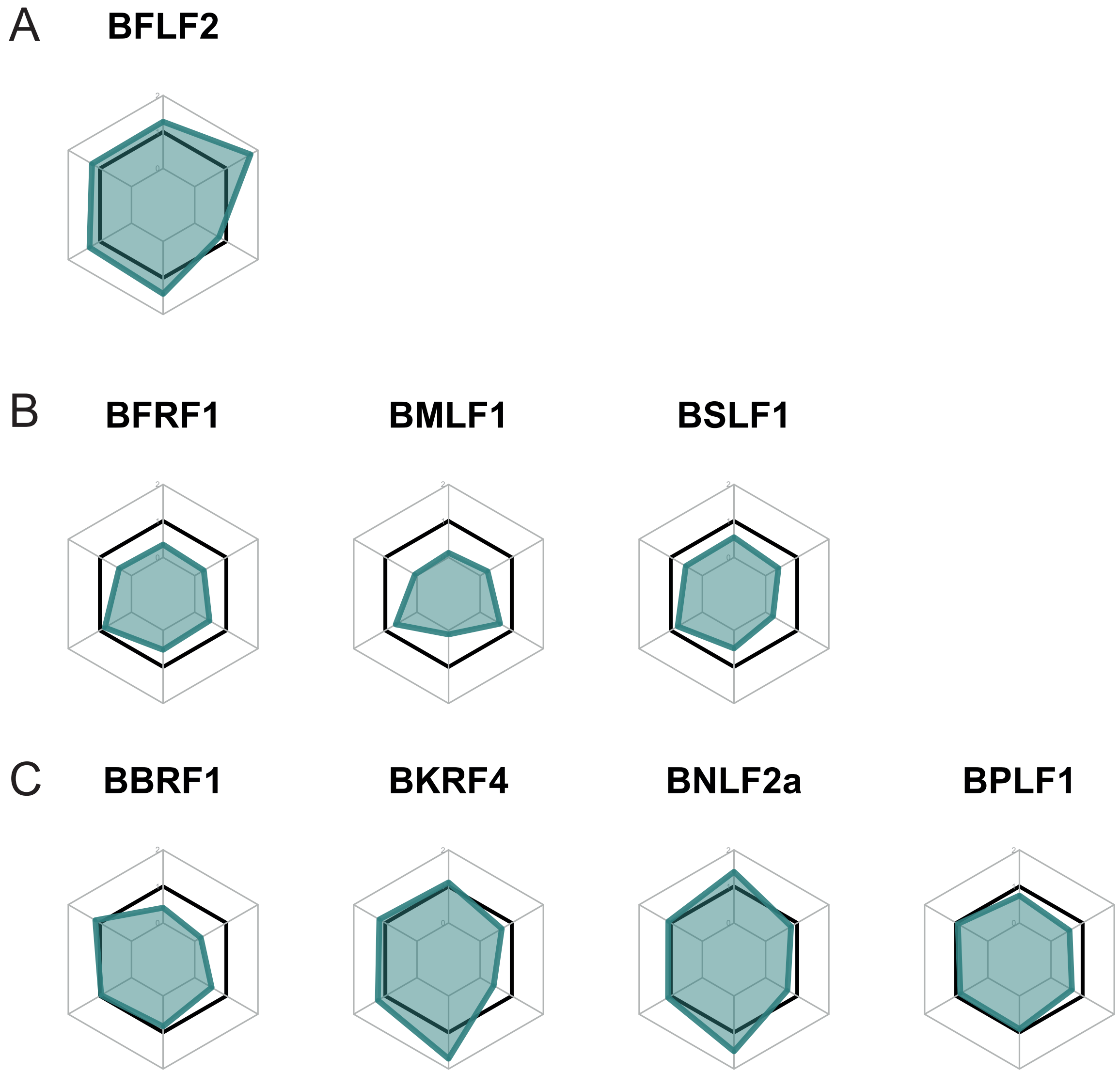

Supplementary Figure S9
