# Supplementary material for: Assessing the fitness of Epstein-Barr virus following its reactivation
Source: J Virol. 2025 May 30;99(7):e00626-25. doi: 10.1128/jvi.00626-25 (PMC12282151; doi:10.1128/jvi.00626-25)
Supplement: Supplemental legends — Legends for Fig. S1 to S9. [file jvi.00626-25-s0002.docx]

**Supplementary Figure legends**

**Supplementary Figure S1. Comparison of physical particle concentrations in virus samples generated by co-transfection of BZLF1 and single expression plasmids from a panel of 77 EBV genes including two controls.**

EB virus stocks were analyzed for their physical particle concentration by nanoparticle tracking analysis (NTA) as shown in Figure 3. EBV genes are marked according to their early or late expression characteristics as indicated. Mean and standard deviation of three biological replicates are shown. The horizontal lines indicate groups of 10 viral genes for better visualization.

**Supplementary Figure S2. Comparison of bioparticle concentrations in virus samples generated by BZLF1 transfection together with expression plasmids from a panel of 77 EBV genes and controls.**

Data are identical to those shown in Figure 5, but EBV genes are color-coded according to their early or late expression characteristics as indicated. The vertical lines provide 0.5- and 1.5-fold ratios. The horizontal lines indicate groups of 10 viral genes for better visualization.

**Supplementary Figure S3. Comparison of virus titers generated by co-transfection of BZLF1 together with expression plasmids from a panel of 77 EBV genes plus two controls.**

Data are identical to those shown in Figure 6 but EBV genes are marked according to their early or late expression characteristics. Two vertical lines indicate 0.5- and 1.5-fold ratios. The horizontal lines indicate groups of 10 viral genes for better visualization.

**Supplementary Figure S4.** **Live and dead cells staining of 2089 HEK293 virus producer cells.** 2089 EBV producer cells were transiently transfected with BZLF1, BALF4 and 11 selected ‘low bin’ EBV gene expression plasmids. The cells were stained with the Far-Red Dead Cell Stain Kit and fluorescence was quantified by flow cytometry. The x-axis shows the APC channel and the y-axis demonstrates cell counts. Non-transfected 2089 EBV producer cells without dye are indicated as ´Unstained´ and the cells with dye are indicated as ‘Stained’. Brackets define the two different populations of living and dead cells. The small population of cells with high fluorescence intensity represents the fraction of dead cells.

**Supplementary Figure S5. Statistical analysis of virus titer, bioparticle concentration, physical particle concentration, functional group category and expression time classification.**

**(A)** Heatmap of Spearman's rank correlation coefficients between virus titer, bioparticle concentration and physical particle concentration measurements of all 77 individual virus supernatants is presented. Numbers in the grids indicate calculated Spearman correlation coefficients. For each measurement, a mean value from 3 replicates was considered prior to ranking and coefficient calculation. **(B)** Boxplots of virus titers (left), bioparticle concentrations (center) and physical particle concentrations (right) of all 77 individual virus supernatants, split by the functions of the viral gene co-expressed together with BZLF1 (DNA or capsid-associated proteins, membrane proteins, tegument proteins, non-structural proteins and unknown function). X-axes indicate the fold changes compared to the reference (Ctrl in Figures 3, 5, and 6). Each individual data point is the mean value of 3 individual measurements. The single red dot indicates the mean value of the virus supernatant obtained by co-transfection of BALF4 with BZLF1. **(C)** Boxplots of virus titers (left), bioparticle concentrations (center) and physical particle concentrations (right) of all 77 individual virus supernatants, split by the expression timing of the gene co-expressed together with BZLF1 (early, late, to be determined). X-axes indicate the fold changes compared to the reference (Ctrl in Figures 3, 5, and 6). The single red dot indicates the mean value of the virus supernatant of BALF4 co-expressed with BZLF1. An unpaired Wilcoxon rank sum test showed no significant differences, comparing the ‘early’ and ‘late’ groups. The median of the different populations is shown.

**Supplementary Figure S6. Functional evaluation of 75 shRNA constructs directed against 25 selected viral transcripts including controls.**

shRNA antisense guides for 25 EBV gene were generated (http://splashrna.mskcc.org) and three shRNA sequences per EBV target gene were individually cloned into the miR-3G frame of the basic shRNA vector (pCDH; p6924), which also encodes resistance against puromycin. A set of corresponding EBV target genes cloned into the psiCHECK2 dual luciferase reporter allows functional testing of the chosen shRNAs. **(A)** The flow chart depicts the experimental setup of the reporter assay shown in this panel. Transient reporter assays were conducted in 24-well cluster plates seeded with 2x10^5^ 293T cells, which were co-transfected with 25 individual reporter plasmids together with 25 single matching lentiviral shRNA expression plasmids. As reference and control, an empty pCDH lentiviral plasmid (p6924) was co-transfected with the indicated luciferase reporter (Ctrl). As additional controls, luciferase reporter plasmid with GFP and BALF4 as target genes were used in combination with three individual lentiviral vectors encoding GFP- and BALF4-specific shRNAs. After 24 h, luciferase activities were recorded and normalized to the reference. ‘High bin’ and ‘low bin’ samples are indicated. **(B)** The flow chart shows the experimental setup of introducing sets of three shRNA encoding lentiviruses into the 2089 EBV producer cell line and selection with puromycin. The individual cell lines stably transduced with three shRNA vectors each were analyzed by transient transfection with matching dual luciferase reporter plasmids encoding viral targets as in panel A. Mean and standard deviation of three biological replicates are shown. Asterisks indicate statistical significance as determined by using the unpaired two-tailed t test with Welch’s correction (**P≤ 0.01; ***P≤ 0.001). **(C)** The effect of three shRNAs stably introduced into the 2089 EBV producer cell line and directed against GFP was analyzed by flow cytometry. GFP-negative cells were used as control. GFP expression of parental 2089 EBV producer cells (2089) and a derivative stably transduced with an empty pCDH lentiviral plasmid (p6924) as an shRNA control (shCtrl) are shown. The x-axis denotes intensity of GFP fluorescence.

**Supplementary Figure S7. Comparison of the infectivity of engineered EBV stocks generated from 2089 EBV producer cells after dual transfection of BZLF1 and single expression plasmids or after triple transfection with BZLF1, single expression plasmids and CD63:BlaM.**

Supernatants analyzed in panel B of Figure 8 were tested for their infectivity using Raji cells and GFP expression as indicator of viral infection as schematically shown in panel A of Figure 8. For comparison, the 2089 EBV producer cell line was transiently transfected with 25 individual expression plasmids encoding viral genes of the ‘high bin’, ‘low bin’ groups and three ‘random’ genes together with BZLF1 p509, only, omitting the CD63:ß-lactamase (CD63:BlaM) expression plasmid p7200. The pairwise comparison of supernatants generated with and without CD63:BlaM is shown.

**Supplementary Figure S8. Comparison of the fusogenicity of virus stocks from 2089 EBV producer cells and HH514 cells.** 2089 EBV producer cells were transiently transfected with two or three plasmids encoding BZLF1 (p509), CD63:HiBiT (p7544) or BZLF1, CD63:HiBiT and BALF4 (p6515) as indicated. HH514 cells were electroporated with the identical plasmid combinations. CD63:LgBiT Daudi cells as recipients were incubated with the virus stocks for 24 h prior to luminescence measurement. As a background control, CD63:LgBiT Daudi cells were incubated with RPMI culture medium, only. Mean and standard deviation of three biological replicates are shown.

**Supplementary Figure S9. Additional spider charts visualizing the results of six parameters derived from the analysis of selected viral genes.**

Shown are additional charts with parameter ratios obtained from 8 viral genes supplementing data shown in main Figure 10.
