# Supplementary material for: Assessing the fitness of Epstein-Barr virus following its reactivation
Source: J Virol. 2025 May 30;99(7):e00626-25. doi: 10.1128/jvi.00626-25 (PMC12282151; doi:10.1128/jvi.00626-25)
Supplement: Table S2 — Overview of synthetic oligonucleotides cloned into the basic lentiviral shRNA expression vector plasmid p6924 to be stably introduced into 2089 EBV producer cells. [file jvi.00626-25-s0004.docx]

**Table S2. Overview of synthetic oligonucleotides cloned into the basic lentiviral shRNA expression vector plasmid p6924 to be stably introduced into 2089 EBV producer cells**

| Target gene (plasmid #) | Oligonucleotide |
| --- | --- |
| BALF4 (p6515) | shRNA 6954 for  CTAGGTTTATGTTTGGATGAACTGACATACGCGTATCCGTCTAATGTTGTCTTTAAACACCATGTAGTGAAATATATATTAAAC﻿﻿ATGGTGTTTAAAGACAACATTTTACGGTAACGCGG |
|  | shRNA 6954 rev  AATTCCGCGTTACCGTA﻿﻿﻿AAATGTTGTCTTTAAACACCATGTTTAATATATATTTCACTAC﻿﻿ATGGTGTTTAAAGACAACATTAGACGGATACGCGTATGTCAGTTCATCCAAACATAAAC |
|  | shRNA 6955 for  CTAGGTTTATGTTTGGATGAACTGACATACGCGTATCCGTC﻿TTTAGTGAGATGAAGGTCTGCAGTAGTGAAATATATATTAAAC﻿﻿﻿TGCAGACCTTCATCTCACTAATTACGGTAACGCGG |
|  | shRNA 6955 rev  AATTCCGCGTTACCGTA﻿﻿﻿﻿ATTAGTGAGATGAAGGTCTGCAGTTTAATATATATTTCACTAC﻿﻿﻿TGCAGACCTTCATCTCACTAAAGACGGATACGCGTATGTCAGTTCATCCAAACATAAAC |
|  | shRNA 6956 for  CTAGGTTTATGTTTGGATGAACTGACATACGCGTATCCGTC﻿TTATAAAATATGTAATGGCTTCGTAGTGAAATATATATTAAAC﻿﻿﻿GAAGCCATTACATATTTTATATTACGGTAACGCGG |
|  | shRNA 6956 rev  AATTCCGCGTTACCGTA﻿﻿﻿﻿ATATAAAATATGTAATGGCTTCGTTTAATATATATTTCACTAC﻿﻿﻿GAAGCCATTACATATTTTATAAGACGGATACGCGTATGTCAGTTCATCCAAACATAAAC |
| BKRF4 (p6562) | shRNA 7296 top  CTAGGTTTATGTTTGGATGAACTGACATACGCGTATCCGTC﻿TGTGTTTATTGTATGTATTGGGGTAGTGAAATATATATTAAAC﻿﻿﻿CCCAATACATACAATAAACACTTACGGTAACGCGG |
|  | shRNA 7296 bot  AATTCCGCGTTACCGTA﻿﻿﻿﻿AGTGTTTATTGTATGTATTGGGGTTTAATATATATTTCACTAC﻿﻿﻿CCCAATACATACAATAAACACAGACGGATACGCGTATGTCAGTTCATCCAAACATAAAC |
|  | shRNA 7297 top  CTAGGTTTATGTTTGGATGAACTGACATACGCGTATCCGTC﻿TTTATTGTATGTATTGGGACTTGTAGTGAAATATATATTAAAC﻿﻿﻿AAGTCCCAATACATACAATAATTACGGTAACGCGG |
|  | shRNA 7297 bot  AATTCCGCGTTACCGTA﻿﻿﻿﻿ATTATTGTATGTATTGGGACTTGTTTAATATATATTTCACTAC﻿﻿﻿AAGTCCCAATACATACAATAAAGACGGATACGCGTATGTCAGTTCATCCAAACATAAAC |
|  | shRNA 7298 top  CTAGGTTTATGTTTGGATGAACTGACATACGCGTATCCGTC﻿TATTGTATGTATTGGGACTTGAGTAGTGAAATATATATTAAAC﻿﻿﻿TCAAGTCCCAATACATACAATTTACGGTAACGCGG |
|  | shRNA 7298 bot  AATTCCGCGTTACCGTA﻿﻿﻿﻿AATTGTATGTATTGGGACTTGAGTTTAATATATATTTCACTAC﻿﻿﻿TCAAGTCCCAATACATACAATAGACGGATACGCGTATGTCAGTTCATCCAAACATAAAC |
| BVLF1 (p6846) | shRNA 7299 top  CTAGGTTTATGTTTGGATGAACTGACATACGCGTATCCGTC﻿﻿TAAATAAACTCATCGCACGGGGGTAGTGAAATATATATTAAAC﻿﻿﻿CCCCGTGCGATGAGTTTATTTTTACGGTAACGCGG |
|  | shRNA 7299 bot  AATTCCGCGTTACCGTA﻿﻿﻿﻿AAAATAAACTCATCGCACGGGGGTTTAATATATATTTCACTACCCCCGTGCGATGAGTTTATTTAGACGGATACGCGTATGTCAGTTCATCCAAACATAAAC |
|  | shRNA 7300 top  CTAGGTTTATGTTTGGATGAACTGACATACGCGTATCCGTC﻿TTCTTTAGCATCTTCAGGAGGAGTAGTGAAATATATATTAAAC﻿﻿﻿TCCTCCTGAAGATGCTAAAGATTACGGTAACGCGG |
|  | shRNA 7300 bot  AATTCCGCGTTACCGTA﻿﻿﻿﻿ATCTTTAGCATCTTCAGGAGGAGTTTAATATATATTTCACTACTCCTCCTGAAGATGCTAAAGAAGACGGATACGCGTATGTCAGTTCATCCAAACATAAAC |
|  | shRNA 7301 top  CTAGGTTTATGTTTGGATGAACTGACATACGCGTATCCGTC﻿﻿TAAAAGTTAACACTTAGGGTCAGTAGTGAAATATATATTAAAC﻿﻿﻿TGACCCTAAGTGTTAACTTTTTTACGGTAACGCGG |
|  | shRNA 7301 bot  AATTCCGCGTTACCGTA﻿﻿﻿﻿AAAAAGTTAACACTTAGGGTCAGTTTAATATATATTTCACTACTGACCCTAAGTGTTAACTTTTAGACGGATACGCGTATGTCAGTTCATCCAAACATAAAC |
| BNLF2a (p6839) | shRNA 7302 top  CTAGGTTTATGTTTGGATGAACTGACATACGCGTATCCGTC﻿﻿﻿TTTATTTATTGCATCACAAGTCGTAGTGAAATATATATTAAAC﻿﻿﻿﻿GACTTGTGATGCAATAAATAATTACGGTAACGCGG |
|  | shRNA 7302 bot  AATTCCGCGTTACCGTA﻿﻿﻿﻿﻿ATTATTTATTGCATCACAAGTCGTTTAATATATATTTCACTAC﻿GACTTGTGATGCAATAAATAAAGACGGATACGCGTATGTCAGTTCATCCAAACATAAAC |
|  | shRNA 7303 top  CTAGGTTTATGTTTGGATGAACTGACATACGCGTATCCGTC﻿﻿TTATTTATTGCATCACAAGTCAGTAGTGAAATATATATTAAAC﻿﻿﻿﻿TGACTTGTGATGCAATAAATATTACGGTAACGCGG |
|  | shRNA 7303 bot  AATTCCGCGTTACCGTA﻿﻿﻿﻿﻿ATATTTATTGCATCACAAGTCAGTTTAATATATATTTCACTAC﻿TGACTTGTGATGCAATAAATAAGACGGATACGCGTATGTCAGTTCATCCAAACATAAAC |
|  | shRNA 7304 top  CTAGGTTTATGTTTGGATGAACTGACATACGCGTATCCGTC﻿﻿﻿TTTATTGCATCACAAGTCACATGTAGTGAAATATATATTAAAC﻿﻿﻿﻿ATGTGACTTGTGATGCAATAATTACGGTAACGCGG |
|  | shRNA 7304 bot  AATTCCGCGTTACCGTA﻿﻿﻿﻿﻿ATTATTGCATCACAAGTCACATGTTTAATATATATTTCACTAC﻿ATGTGACTTGTGATGCAATAAAGACGGATACGCGTATGTCAGTTCATCCAAACATAAAC |
| BXRF1 (p6848) | shRNA 7305 top  CTAGGTTTATGTTTGGATGAACTGACATACGCGTATCCGTC﻿﻿﻿﻿TTGTAAATCTTATTGTCCGCGAGTAGTGAAATATATATTAAAC﻿﻿﻿﻿﻿TCGCGGACAATAAGATTTACATTACGGTAACGCGG |
|  | shRNA 7305 bot  AATTCCGCGTTACCGTA﻿﻿﻿﻿﻿﻿ATGTAAATCTTATTGTCCGCGAGTTTAATATATATTTCACTAC﻿﻿TCGCGGACAATAAGATTTACAAGACGGATACGCGTATGTCAGTTCATCCAAACATAAAC |
|  | shRNA 7306 top  CTAGGTTTATGTTTGGATGAACTGACATACGCGTATCCGTC﻿﻿﻿TTAAATTCTACAATATAACACCGTAGTGAAATATATATTAAAC﻿﻿﻿﻿﻿GGTGTTATATTGTAGAATTTATTACGGTAACGCGG |
|  | shRNA 7306 bot  AATTCCGCGTTACCGTA﻿﻿﻿﻿﻿﻿ATAAATTCTACAATATAACACCGTTTAATATATATTTCACTAC﻿﻿GGTGTTATATTGTAGAATTTAAGACGGATACGCGTATGTCAGTTCATCCAAACATAAAC |
|  | shRNA 7307 top  CTAGGTTTATGTTTGGATGAACTGACATACGCGTATCCGTC﻿﻿﻿﻿TTGATAATCTCAAAGAGGGTGTGTAGTGAAATATATATTAAAC﻿﻿﻿﻿﻿ACACCCTCTTTGAGATTATCATTACGGTAACGCGG |
|  | shRNA 7307 bot  AATTCCGCGTTACCGTA﻿﻿﻿﻿﻿﻿ATGATAATCTCAAAGAGGGTGTGTTTAATATATATTTCACTAC﻿ACACCCTCTTTGAGATTATCAAGACGGATACGCGTATGTCAGTTCATCCAAACATAAAC |
| BFLF2 (p6644) | shRNA 7308 top  CTAGGTTTATGTTTGGATGAACTGACATACGCGTATCCGTC﻿﻿﻿﻿﻿TTATTTTCCAAAATGAGCTGGGGTAGTGAAATATATATTAAAC﻿﻿﻿﻿﻿﻿CCCAGCTCATTTTGGAAAATATTACGGTAACGCGG |
|  | shRNA 7308 bot  AATTCCGCGTTACCGTA﻿﻿﻿﻿﻿﻿﻿ATATTTTCCAAAATGAGCTGGGGTTTAATATATATTTCACTAC﻿﻿﻿CCCAGCTCATTTTGGAAAATAAGACGGATACGCGTATGTCAGTTCATCCAAACATAAAC |
|  | shRNA 7309 top  CTAGGTTTATGTTTGGATGAACTGACATACGCGTATCCGTC﻿﻿﻿﻿TTTATTTTCCAAAATGAGCTGGGTAGTGAAATATATATTAAAC﻿﻿﻿﻿﻿﻿CCAGCTCATTTTGGAAAATAATTACGGTAACGCGG |
|  | shRNA 7309 bot  AATTCCGCGTTACCGTA﻿﻿﻿﻿﻿﻿﻿ATTATTTTCCAAAATGAGCTGGGTTTAATATATATTTCACTAC﻿﻿﻿CCAGCTCATTTTGGAAAATAAAGACGGATACGCGTATGTCAGTTCATCCAAACATAAAC |
|  | shRNA 7310 top  CTAGGTTTATGTTTGGATGAACTGACATACGCGTATCCGTC﻿﻿﻿﻿﻿TTTGATAGGACTGTACCAGGTCGTAGTGAAATATATATTAAAC﻿﻿﻿﻿﻿﻿GACCTGGTACAGTCCTATCAATTACGGTAACGCGG |
|  | shRNA 7310 bot  AATTCCGCGTTACCGTA﻿﻿﻿﻿﻿﻿﻿ATTGATAGGACTGTACCAGGTCGTTTAATATATATTTCACTAC﻿GACCTGGTACAGTCCTATCAAAGACGGATACGCGTATGTCAGTTCATCCAAACATAAAC |
| BMRF1 (p5106) | shRNA 7311 top (same as 6963 for)  CTAGGTTTATGTTTGGATGAACTGACATACGCGTATCCGTC﻿﻿﻿﻿﻿﻿TAGGATTTAATGAATGTCACCAGTAGTGAAATATATATTAAAC﻿﻿﻿﻿﻿﻿﻿TGGTGACATTCATTAAATCCTTTACGGTAACGCGG |
|  | shRNA 7311 bot (same as 6963 rev)  AATTCCGCGTTACCGTA﻿﻿﻿﻿﻿﻿﻿﻿AAGGATTTAATGAATGTCACCAGTTTAATATATATTTCACTAC﻿﻿﻿﻿TGGTGACATTCATTAAATCCTAGACGGATACGCGTATGTCAGTTCATCCAAACATAAAC |
|  | shRNA 7312 top  CTAGGTTTATGTTTGGATGAACTGACATACGCGTATCCGTC﻿﻿﻿﻿﻿TAAAATAACACTAAGATCCAACGTAGTGAAATATATATTAAAC﻿﻿﻿﻿﻿﻿﻿GTTGGATCTTAGTGTTATTTTTTACGGTAACGCGG |
|  | shRNA 7312 bot  AATTCCGCGTTACCGTA﻿﻿﻿﻿﻿﻿﻿﻿AAAAATAACACTAAGATCCAACGTTTAATATATATTTCACTAC﻿GTTGGATCTTAGTGTTATTTTAGACGGATACGCGTATGTCAGTTCATCCAAACATAAAC |
|  | shRNA 7313 top  CTAGGTTTATGTTTGGATGAACTGACATACGCGTATCCGTC﻿﻿﻿﻿﻿﻿TTATCATCATATTCCATAGTGAGTAGTGAAATATATATTAAAC﻿﻿﻿﻿﻿﻿﻿TCACTATGGAATATGATGATATTACGGTAACGCGG |
|  | shRNA 7313 bot  AATTCCGCGTTACCGTA﻿﻿﻿﻿﻿﻿﻿﻿ATATCATCATATTCCATAGTGAGTTTAATATATATTTCACTAC﻿﻿TCACTATGGAATATGATGATAAGACGGATACGCGTATGTCAGTTCATCCAAACATAAAC |
| BHLF1 (p7080) | shRNA 7314 top  CTAGGTTTATGTTTGGATGAACTGACATACGCGTATCCGTC﻿﻿﻿﻿﻿﻿﻿TTAAACAGTTTATTGATAGGTGGTAGTGAAATATATATTAAAC﻿﻿﻿﻿﻿﻿﻿﻿CACCTATCAATAAACTGTTTATTACGGTAACGCGG |
|  | shRNA 7314 bot  AATTCCGCGTTACCGTA﻿﻿﻿﻿﻿﻿﻿﻿﻿ATAAACAGTTTATTGATAGGTGGTTTAATATATATTTCACTAC﻿﻿﻿﻿﻿CACCTATCAATAAACTGTTTAAGACGGATACGCGTATGTCAGTTCATCCAAACATAAAC |
|  | shRNA 7315 top  CTAGGTTTATGTTTGGATGAACTGACATACGCGTATCCGTC﻿﻿﻿﻿﻿﻿TTGACATGTAGGTGAGTAGTGTGTAGTGAAATATATATTAAAC﻿﻿﻿﻿﻿﻿﻿﻿ACACTACTCACCTACATGTCATTACGGTAACGCGG |
|  | shRNA 7315 bot  AATTCCGCGTTACCGTA﻿﻿﻿﻿﻿﻿﻿﻿﻿ATGACATGTAGGTGAGTAGTGTGTTTAATATATATTTCACTAC﻿﻿ACACTACTCACCTACATGTCAAGACGGATACGCGTATGTCAGTTCATCCAAACATAAAC |
|  | shRNA 7316 top  CTAGGTTTATGTTTGGATGAACTGACATACGCGTATCCGTC﻿﻿﻿﻿﻿﻿﻿TTACTTTTAGAGTGTAGTGTACGTAGTGAAATATATATTAAAC﻿﻿﻿﻿﻿﻿﻿﻿GTACACTACACTCTAAAAGTATTACGGTAACGCGG |
|  | shRNA 7316 bot  AATTCCGCGTTACCGTA﻿﻿﻿﻿﻿﻿﻿﻿﻿ATACTTTTAGAGTGTAGTGTACGTTTAATATATATTTCACTAC﻿GTACACTACACTCTAAAAGTAAGACGGATACGCGTATGTCAGTTCATCCAAACATAAAC |
| BXLF1 (p6571) | shRNA 7320 top  CTAGGTTTATGTTTGGATGAACTGACATACGCGTATCCGTC﻿﻿﻿﻿﻿﻿﻿﻿TTAAGTACACTAAAGATGCTGTGTAGTGAAATATATATTAAAC﻿﻿﻿﻿﻿﻿﻿﻿﻿ACAGCATCTTTAGTGTACTTATTACGGTAACGCGG |
|  | shRNA 7320 bot  AATTCCGCGTTACCGTA﻿﻿﻿﻿﻿﻿﻿﻿﻿﻿ATAAGTACACTAAAGATGCTGTGTTTAATATATATTTCACTAC﻿﻿﻿﻿﻿﻿ACAGCATCTTTAGTGTACTTAAGACGGATACGCGTATGTCAGTTCATCCAAACATAAAC |
|  | shRNA 7321 top  CTAGGTTTATGTTTGGATGAACTGACATACGCGTATCCGTC﻿﻿﻿﻿﻿﻿﻿TTGTAAATTAACTTGTAGCGGTGTAGTGAAATATATATTAAAC﻿﻿﻿﻿﻿﻿﻿﻿﻿ACCGCTACAAGTTAATTTACATTACGGTAACGCGG |
|  | shRNA 7321 bot  AATTCCGCGTTACCGTA﻿﻿﻿﻿﻿﻿﻿﻿﻿﻿ATGTAAATTAACTTGTAGCGGTGTTTAATATATATTTCACTAC﻿﻿﻿ACCGCTACAAGTTAATTTACAAGACGGATACGCGTATGTCAGTTCATCCAAACATAAAC |
|  | shRNA 7322 top  CTAGGTTTATGTTTGGATGAACTGACATACGCGTATCCGTC﻿﻿﻿﻿﻿﻿﻿﻿TAACTTACTAAACTCGCGCCCAGTAGTGAAATATATATTAAAC﻿﻿﻿﻿﻿﻿﻿﻿﻿TGGGCGCGAGTTTAGTAAGTTTTACGGTAACGCGG |
|  | shRNA 7322 bot  AATTCCGCGTTACCGTA﻿﻿﻿﻿﻿﻿﻿﻿﻿﻿AAACTTACTAAACTCGCGCCCAGTTTAATATATATTTCACTAC﻿﻿TGGGCGCGAGTTTAGTAAGTTAGACGGATACGCGTATGTCAGTTCATCCAAACATAAAC |
| BVRF2 (p6847) | shRNA 7264 top  CTAGGTTTATGTTTGGATGAACTGACATACGCGTATCCGTC﻿﻿﻿TTTAAATACGACTCGGCTGGGAGTAGTGAAATATATATTAAAC﻿﻿﻿TCCCAGCCGAGTCGTATTTAATTACGGTAACGCGG |
|  | shRNA 7264 bot  AATTCCGCGTTACCGTA﻿﻿﻿﻿ATTAAATACGACTCGGCTGGGAGTTTAATATATATTTCACTAC﻿﻿﻿TCCCAGCCGAGTCGTATTTAAAGACGGATACGCGTATGTCAGTTCATCCAAACATAAAC |
|  | shRNA 7265top  CTAGGTTTATGTTTGGATGAACTGACATACGCGTATCCGTC﻿﻿﻿﻿TAAGTGTTAACTTTTACCTGTGGTAGTGAAATATATATTAAAC﻿﻿﻿﻿CACAGGTAAAAGTTAACACTTTTACGGTAACGCGG |
|  | shRNA 7265 bot  AATTCCGCGTTACCGTA﻿﻿﻿﻿﻿AAAGTGTTAACTTTTACCTGTGGTTTAATATATATTTCACTAC﻿﻿﻿﻿CACAGGTAAAAGTTAACACTTAGACGGATACGCGTATGTCAGTTCATCCAAACATAAAC |
|  | shRNA 7266 top  CTAGGTTTATGTTTGGATGAACTGACATACGCGTATCCGTC﻿﻿﻿TTTGATGAGGCTGAAATCCGTAGTAGTGAAATATATATTAAAC﻿﻿﻿TACGGATTTCAGCCTCATCAATTACGGTAACGCGG |
|  | shRNA 7266 bot  AATTCCGCGTTACCGTA﻿﻿﻿ATTGATGAGGCTGAAATCCGTAGTTTAATATATATTTCACTAC﻿﻿﻿TACGGATTTCAGCCTCATCAAAGACGGATACGCGTATGTCAGTTCATCCAAACATAAAC |
| BALF2 (p6927) | shRNA 7323 top  CTAGGTTTATGTTTGGATGAACTGACATACGCGTATCCGTC﻿﻿﻿﻿﻿﻿﻿﻿﻿TTCTCAATCTCATATGTGGTCGGTAGTGAAATATATATTAAAC﻿﻿﻿﻿﻿﻿﻿﻿﻿﻿CGACCACATATGAGATTGAGATTACGGTAACGCGG |
|  | shRNA 7323 bot  AATTCCGCGTTACCGTA﻿﻿﻿﻿﻿﻿﻿﻿﻿﻿﻿ATCTCAATCTCATATGTGGTCGGTTTAATATATATTTCACTAC﻿﻿﻿﻿﻿﻿﻿CGACCACATATGAGATTGAGAAGACGGATACGCGTATGTCAGTTCATCCAAACATAAAC |
|  | shRNA 7324 top  CTAGGTTTATGTTTGGATGAACTGACATACGCGTATCCGTC﻿﻿﻿﻿﻿﻿﻿﻿TTCTTGATCTTGATGTTCCTGGGTAGTGAAATATATATTAAAC﻿﻿﻿﻿﻿﻿﻿﻿﻿﻿CCAGGAACATCAAGATCAAGATTACGGTAACGCGG |
|  | shRNA 7324 bot  AATTCCGCGTTACCGTA﻿﻿﻿﻿﻿﻿﻿﻿﻿﻿﻿ATCTTGATCTTGATGTTCCTGGGTTTAATATATATTTCACTAC﻿﻿﻿﻿CCAGGAACATCAAGATCAAGAAGACGGATACGCGTATGTCAGTTCATCCAAACATAAAC |
|  | shRNA 7325 top  CTAGGTTTATGTTTGGATGAACTGACATACGCGTATCCGTC﻿﻿﻿﻿﻿﻿﻿﻿﻿TTCATAAACTGGACCACTTCGGGTAGTGAAATATATATTAAAC﻿﻿﻿﻿﻿﻿﻿﻿﻿﻿CCGAAGTGGTCCAGTTTATGATTACGGTAACGCGG |
|  | shRNA 7325 bot  AATTCCGCGTTACCGTA﻿﻿﻿﻿﻿﻿﻿﻿﻿﻿﻿ATCATAAACTGGACCACTTCGGGTTTAATATATATTTCACTAC﻿﻿﻿CCGAAGTGGTCCAGTTTATGAAGACGGATACGCGTATGTCAGTTCATCCAAACATAAAC |
| BPLF1 (p6567) | shRNA 6975 top  CTAGGTTTATGTTTGGATGAACTGACATACGCGTATCCGTC﻿﻿TTAATAAACAATTACAGATACAGTAGTGAAATATATATTAAAC﻿﻿﻿﻿TGTATCTGTAATTGTTTATTATTACGGTAACGCGG |
|  | shRNA 6975 bot  AATTCCGCGTTACCGTA﻿﻿﻿﻿﻿ATAATAAACAATTACAGATACAGTTTAATATATATTTCACTAC﻿﻿﻿﻿TGTATCTGTAATTGTTTATTAAGACGGATACGCGTATGTCAGTTCATCCAAACATAAAC |
|  | shRNA 6976 top  CTAGGTTTATGTTTGGATGAACTGACATACGCGTATCCGTC﻿﻿TTTATTAATAAACAATTACAGAGTAGTGAAATATATATTAAAC﻿﻿﻿﻿﻿TCTGTAATTGTTTATTAATAATTACGGTAACGCGG |
|  | shRNA 6976 bot  AATTCCGCGTTACCGTA﻿﻿﻿﻿﻿ATTATTAATAAACAATTACAGAGTTTAATATATATTTCACTAC﻿﻿﻿﻿TCTGTAATTGTTTATTAATAAAGACGGATACGCGTATGTCAGTTCATCCAAACATAAAC |
|  | shRNA 6977 top  CTAGGTTTATGTTTGGATGAACTGACATACGCGTATCCGTC﻿﻿TTCAATAGACAGAAATTGGGTGGTAGTGAAATATATATTAAAC﻿﻿﻿﻿﻿CACCCAATTTCTGTCTATTGATTACGGTAACGCGG |
|  | shRNA 6977 bot  AATTCCGCGTTACCGTA﻿﻿﻿﻿﻿ATCAATAGACAGAAATTGGGTGGTTTAATATATATTTCACTAC﻿﻿﻿﻿CACCCAATTTCTGTCTATTGAAGACGGATACGCGTATGTCAGTTCATCCAAACATAAAC |
| BBRF1 (p6593) | shRNA 6978 for  CTAGGTTTATGTTTGGATGAACTGACATACGCGTATCCGTC﻿﻿TAAAGAATCTTGATAAAACAGGGTAGTGAAATATATATTAAAC﻿﻿﻿﻿CCTGTTTTATCAAGATTCTTTTTACGGTAACGCGG |
|  | shRNA 6978 rev  AATTCCGCGTTACCGTA﻿﻿﻿﻿﻿AAAAGAATCTTGATAAAACAGGGTTTAATATATATTTCACTAC﻿﻿﻿﻿CCTGTTTTATCAAGATTCTTTAGACGGATACGCGTATGTCAGTTCATCCAAACATAAAC |
|  | shRNA 6979 for  CTAGGTTTATGTTTGGATGAACTGACATACGCGTATCCGTC﻿﻿TTGGCATTCTCAATCATCGAGAGTAGTGAAATATATATTAAAC﻿﻿﻿﻿TCTCGATGATTGAGAATGCCATTACGGTAACGCGG |
|  | shRNA 6979 rev  AATTCCGCGTTACCGTA﻿﻿﻿﻿﻿ATGGCATTCTCAATCATCGAGAGTTTAATATATATTTCACTAC﻿﻿﻿﻿TCTCGATGATTGAGAATGCCAAGACGGATACGCGTATGTCAGTTCATCCAAACATAAAC |
|  | shRNA 6980 for  CTAGGTTTATGTTTGGATGAACTGACATACGCGTATCCGTC﻿TTCATGTTGAACATGACCTCAGGTAGTGAAATATATATTAAAC﻿﻿﻿﻿CTGAGGTCATGTTCAACATGATTACGGTAACGCGG |
|  | shRNA 6980 rev  AATTCCGCGTTACCGTA﻿﻿﻿﻿﻿ATCATGTTGAACATGACCTCAGGTTTAATATATATTTCACTAC﻿﻿﻿﻿CTGAGGTCATGTTCAACATGAAGACGGATACGCGTATGTCAGTTCATCCAAACATAAAC |
| BGLF4 (p6561) | shRNA 6969 top (same as 6966 top)  CTAGGTTTATGTTTGGATGAACTGACATACGCGTATCCGTCTTGATGAAGATGTTGACTGGGAGTAGTGAAATATATATTAAAC﻿﻿﻿TCCCAGTCAACATCTTCATCATTACGGTAACGCGG |
|  | shRNA 6969 bot (same as 6966 bot)  AATTCCGCGTTACCGTA﻿﻿﻿ATGATGAAGATGTTGACTGGGAGTTTAATATATATTTCACTAC﻿﻿TCCCAGTCAACATCTTCATCAAGACGGATACGCGTATGTCAGTTCATCCAAACATAAAC |
|  | shRNA 6970 top  CTAGGTTTATGTTTGGATGAACTGACATACGCGTATCCGTCTAAATCTGATAAATGACCTCTTGTAGTGAAATATATATTAAAC﻿﻿﻿AAGAGGTCATTTATCAGATTTTTACGGTAACGCGG |
|  | shRNA 6970 bot  AATTCCGCGTTACCGTA﻿﻿﻿AAAATCTGATAAATGACCTCTTGTTTAATATATATTTCACTAC﻿﻿AAGAGGTCATTTATCAGATTTAGACGGATACGCGTATGTCAGTTCATCCAAACATAAAC |
|  | shRNA 6971 top  CTAGGTTTATGTTTGGATGAACTGACATACGCGTATCCGTC﻿TGAAGTAATCAATGACAGTCACGTAGTGAAATATATATTAAAC﻿﻿﻿﻿GTGACTGTCATTGATTACTTCTTACGGTAACGCGG |
|  | shRNA 6971 bot  AATTCCGCGTTACCGTA﻿﻿﻿AGAAGTAATCAATGACAGTCACGTTTAATATATATTTCACTAC﻿GTGACTGTCATTGATTACTTCAGACGGATACGCGTATGTCAGTTCATCCAAACATAAAC |
| BFRF1 (p6645) | shRNA 6992 top  CTAGGTTTATGTTTGGATGAACTGACATACGCGTATCCGTC﻿﻿﻿TTTATTAATAAAGTGCATACACGTAGTGAAATATATATTAAAC﻿﻿﻿﻿﻿GTGTATGCACTTTATTAATAATTACGGTAACGCGG |
|  | shRNA 6992 bot  AATTCCGCGTTACCGTA﻿﻿﻿﻿﻿﻿ATTATTAATAAAGTGCATACACGTTTAATATATATTTCACTAC﻿﻿﻿﻿﻿GTGTATGCACTTTATTAATAAAGACGGATACGCGTATGTCAGTTCATCCAAACATAAAC |
|  | shRNA 6993 top  CTAGGTTTATGTTTGGATGAACTGACATACGCGTATCCGTC﻿﻿﻿TTGGATATCACAAACACGGGCGGTAGTGAAATATATATTAAAC﻿﻿﻿﻿﻿﻿CGCCCGTGTTTGTGATATCCATTACGGTAACGCGG |
|  | shRNA 6993 bot  AATTCCGCGTTACCGTA﻿﻿﻿﻿﻿﻿ATGGATATCACAAACACGGGCGGTTTAATATATATTTCACTAC﻿﻿﻿﻿﻿CGCCCGTGTTTGTGATATCCAAGACGGATACGCGTATGTCAGTTCATCCAAACATAAAC |
|  | shRNA 6994 top  CTAGGTTTATGTTTGGATGAACTGACATACGCGTATCCGTC﻿﻿﻿TTGAAATTTAGGAAGCAGGGGAGTAGTGAAATATATATTAAAC﻿﻿﻿﻿﻿﻿TCCCCTGCTTCCTAAATTTCATTACGGTAACGCGG |
|  | shRNA 6994 bot  AATTCCGCGTTACCGTA﻿﻿﻿﻿﻿﻿ATGAAATTTAGGAAGCAGGGGAGTTTAATATATATTTCACTAC﻿﻿﻿﻿﻿TCCCCTGCTTCCTAAATTTCAAGACGGATACGCGTATGTCAGTTCATCCAAACATAAAC |
| BSLF1 (p6843) | shRNA 7001 top  CTAGGTTTATGTTTGGATGAACTGACATACGCGTATCCGTC﻿﻿﻿﻿TGATTTGAAAAAATAGACTGGGGTAGTGAAATATATATTAAAC﻿﻿﻿﻿﻿﻿CCCAGTCTATTTTTTCAAATCTTACGGTAACGCGG |
|  | shRNA 7001 bot  AATTCCGCGTTACCGTA﻿﻿﻿﻿﻿﻿﻿AGATTTGAAAAAATAGACTGGGGTTTAATATATATTTCACTAC﻿﻿﻿﻿﻿﻿CCCAGTCTATTTTTTCAAATCAGACGGATACGCGTATGTCAGTTCATCCAAACATAAAC |
|  | shRNA 7002 top  CTAGGTTTATGTTTGGATGAACTGACATACGCGTATCCGTC﻿﻿﻿﻿TTCTGTGAATAGTACACTGGGGGTAGTGAAATATATATTAAAC﻿﻿﻿﻿﻿﻿﻿CCCCAGTGTACTATTCACAGATTACGGTAACGCGG |
|  | shRNA 7002 bot  AATTCCGCGTTACCGTA﻿﻿﻿﻿﻿﻿﻿ATCTGTGAATAGTACACTGGGGGTTTAATATATATTTCACTAC﻿﻿﻿﻿﻿﻿CCCCAGTGTACTATTCACAGAAGACGGATACGCGTATGTCAGTTCATCCAAACATAAAC |
|  | shRNA 7003 top  CTAGGTTTATGTTTGGATGAACTGACATACGCGTATCCGTC﻿﻿﻿﻿TGTCATTACAAAGTAGTGCCTGGTAGTGAAATATATATTAAAC﻿﻿﻿﻿﻿﻿﻿CAGGCACTACTTTGTAATGACTTACGGTAACGCGG |
|  | shRNA 7003 bot  AATTCCGCGTTACCGTA﻿﻿﻿﻿﻿﻿﻿AGTCATTACAAAGTAGTGCCTGGTTTAATATATATTTCACTAC﻿﻿﻿﻿﻿﻿CAGGCACTACTTTGTAATGACAGACGGATACGCGTATGTCAGTTCATCCAAACATAAAC |
| BTRF1 (p6845) | shRNA 7004 top  CTAGGTTTATGTTTGGATGAACTGACATACGCGTATCCGTC﻿﻿﻿﻿TGACATTTCTCATAATGGTGCCGTAGTGAAATATATATTAAAC﻿﻿﻿﻿﻿﻿GGCACCATTATGAGAAATGTCTTACGGTAACGCGG |
|  | shRNA 7004 bot  AATTCCGCGTTACCGTA﻿﻿﻿﻿﻿﻿﻿AGACATTTCTCATAATGGTGCCGTTTAATATATATTTCACTAC﻿﻿﻿﻿﻿﻿GGCACCATTATGAGAAATGTCAGACGGATACGCGTATGTCAGTTCATCCAAACATAAAC |
|  | shRNA 7005 top  CTAGGTTTATGTTTGGATGAACTGACATACGCGTATCCGTC﻿﻿﻿﻿TTTCCTAGAAACTGTGCCATGGGTAGTGAAATATATATTAAAC﻿﻿﻿﻿﻿﻿﻿CCATGGCACAGTTTCTAGGAATTACGGTAACGCGG |
|  | shRNA 7005 bot  AATTCCGCGTTACCGTA﻿﻿﻿﻿﻿﻿﻿ATTCCTAGAAACTGTGCCATGGGTTTAATATATATTTCACTAC﻿﻿﻿﻿﻿﻿CCATGGCACAGTTTCTAGGAAAGACGGATACGCGTATGTCAGTTCATCCAAACATAAAC |
|  | shRNA 7006 top  CTAGGTTTATGTTTGGATGAACTGACATACGCGTATCCGTC﻿﻿﻿﻿TGTGGAAGACAATCTGTCCCGAGTAGTGAAATATATATTAAAC﻿﻿﻿﻿﻿﻿﻿TCGGGACAGATTGTCTTCCACTTACGGTAACGCGG |
|  | shRNA 7006 bot  AATTCCGCGTTACCGTA﻿﻿﻿﻿﻿﻿﻿AGTGGAAGACAATCTGTCCCGAGTTTAATATATATTTCACTAC﻿﻿﻿﻿﻿﻿TCGGGACAGATTGTCTTCCACAGACGGATACGCGTATGTCAGTTCATCCAAACATAAAC |
| BMRF2 (p6539) | shRNA 6963 for  CTAGGTTTATGTTTGGATGAACTGACATACGCGTATCCGTC﻿TAGGATTTAATGAATGTCACCAGTAGTGAAATATATATTAAAC﻿﻿﻿TGGTGACATTCATTAAATCCTTTACGGTAACGCGG |
|  | shRNA 6963 rev  AATTCCGCGTTACCGTA﻿﻿﻿﻿AAGGATTTAATGAATGTCACCAGTTTAATATATATTTCACTAC﻿﻿﻿TGGTGACATTCATTAAATCCTAGACGGATACGCGTATGTCAGTTCATCCAAACATAAAC |
|  | shRNA 6964 for  CTAGGTTTATGTTTGGATGAACTGACATACGCGTATCCGTC﻿TTCACAAACTTCTTAAGCTTGTGTAGTGAAATATATATTAAAC﻿﻿﻿ACAAGCTTAAGAAGTTTGTGATTACGGTAACGCGG |
|  | shRNA 6964 rev  AATTCCGCGTTACCGTA﻿﻿﻿﻿ATCACAAACTTCTTAAGCTTGTGTTTAATATATATTTCACTAC﻿﻿﻿ACAAGCTTAAGAAGTTTGTGAAGACGGATACGCGTATGTCAGTTCATCCAAACATAAAC |
|  | shRNA 6965 for  CTAGGTTTATGTTTGGATGAACTGACATACGCGTATCCGTC﻿TCACAAACTTCTTAAGCTTGTAGTAGTGAAATATATATTAAAC﻿﻿﻿TACAAGCTTAAGAAGTTTGTGTTACGGTAACGCGG |
|  | shRNA 6965 rev  AATTCCGCGTTACCGTA﻿﻿﻿﻿ACACAAACTTCTTAAGCTTGTAGTTTAATATATATTTCACTAC﻿﻿﻿TACAAGCTTAAGAAGTTTGTGAGACGGATACGCGTATGTCAGTTCATCCAAACATAAAC |
| BBRF3 (p6534) | shRNA 6960 for  CTAGGTTTATGTTTGGATGAACTGACATACGCGTATCCGTCTAAAGTAAAAGCTGTTGCCCAGGTAGTGAAATATATATTAAAC﻿﻿CTGGGCAACAGCTTTTACTTTTTACGGTAACGCGG |
|  | shRNA 6960 rev  AATTCCGCGTTACCGTA﻿﻿﻿AAAAGTAAAAGCTGTTGCCCAGGTTTAATATATATTTCACTAC﻿﻿CTGGGCAACAGCTTTTACTTTAGACGGATACGCGTATGTCAGTTCATCCAAACATAAAC |
|  | shRNA 6961 for  CTAGGTTTATGTTTGGATGAACTGACATACGCGTATCCGTCTAAATAATTTGCAAAGGGCGTGGTAGTGAAATATATATTAAAC﻿﻿﻿CACGCCCTTTGCAAATTATTTTTACGGTAACGCGG |
|  | shRNA 6961 rev  AATTCCGCGTTACCGTA﻿﻿﻿AAAATAATTTGCAAAGGGCGTGGTTTAATATATATTTCACTAC﻿﻿CACGCCCTTTGCAAATTATTTAGACGGATACGCGTATGTCAGTTCATCCAAACATAAAC |
|  | shRNA 6962 for  CTAGGTTTATGTTTGGATGAACTGACATACGCGTATCCGTCTTTTAAATAATTTGCAAAGGGCGTAGTGAAATATATATTAAAC﻿﻿﻿GCCCTTTGCAAATTATTTAAATTACGGTAACGCGG |
|  | shRNA 6962 rev  AATTCCGCGTTACCGTA﻿﻿﻿ATTTAAATAATTTGCAAAGGGCGTTTAATATATATTTCACTAC﻿﻿GCCCTTTGCAAATTATTTAAAAGACGGATACGCGTATGTCAGTTCATCCAAACATAAAC |
| BNRF1 (p6564) | shRNA 6972 top  CTAGGTTTATGTTTGGATGAACTGACATACGCGTATCCGTC﻿TTCAGTGTATGCATAGTCTGGAGTAGTGAAATATATATTAAAC﻿﻿﻿TCCAGACTATGCATACACTGATTACGGTAACGCGG |
|  | shRNA 6972 bot  AATTCCGCGTTACCGTA﻿﻿﻿﻿ATCAGTGTATGCATAGTCTGGAGTTTAATATATATTTCACTAC﻿﻿﻿TCCAGACTATGCATACACTGAAGACGGATACGCGTATGTCAGTTCATCCAAACATAAAC |
|  | shRNA 6973 top  CTAGGTTTATGTTTGGATGAACTGACATACGCGTATCCGTC﻿TTTGTAAATACAGCACACAGGTGTAGTGAAATATATATTAAAC﻿﻿﻿﻿ACCTGTGTGCTGTATTTACAATTACGGTAACGCGG |
|  | shRNA 6973 bot  AATTCCGCGTTACCGTA﻿﻿﻿﻿ATTGTAAATACAGCACACAGGTGTTTAATATATATTTCACTAC﻿﻿﻿ACCTGTGTGCTGTATTTACAAAGACGGATACGCGTATGTCAGTTCATCCAAACATAAAC |
|  | shRNA 6974 top  CTAGGTTTATGTTTGGATGAACTGACATACGCGTATCCGTC﻿TGCTATTGCATTAACGAAGGGAGTAGTGAAATATATATTAAAC﻿﻿﻿﻿TCCCTTCGTTAATGCAATAGCTTACGGTAACGCGG |
|  | shRNA 6974 bot  AATTCCGCGTTACCGTA﻿﻿﻿﻿AGCTATTGCATTAACGAAGGGAGTTTAATATATATTTCACTAC﻿﻿﻿TCCCTTCGTTAATGCAATAGCAGACGGATACGCGTATGTCAGTTCATCCAAACATAAAC |
| BBLF1 (p6557) | shRNA 6966 for  CTAGGTTTATGTTTGGATGAACTGACATACGCGTATCCGTC﻿﻿TTGATGAAGATGTTGACTGGGAGTAGTGAAATATATATTAAAC﻿﻿﻿﻿TCCCAGTCAACATCTTCATCATTACGGTAACGCGG |
|  | shRNA 6966 rev  AATTCCGCGTTACCGTA﻿﻿﻿﻿﻿ATGATGAAGATGTTGACTGGGAGTTTAATATATATTTCACTAC﻿﻿﻿﻿TCCCAGTCAACATCTTCATCAAGACGGATACGCGTATGTCAGTTCATCCAAACATAAAC |
|  | shRNA 6967 for  CTAGGTTTATGTTTGGATGAACTGACATACGCGTATCCGTC﻿﻿TTCATCATTTTCAGAGTCCTCAGTAGTGAAATATATATTAAAC﻿﻿﻿﻿TGAGGACTCTGAAAATGATGATTACGGTAACGCGG |
|  | shRNA 6967 rev  AATTCCGCGTTACCGTA﻿﻿﻿﻿﻿ATCATCATTTTCAGAGTCCTCAGTTTAATATATATTTCACTAC﻿﻿﻿﻿TGAGGACTCTGAAAATGATGAAGACGGATACGCGTATGTCAGTTCATCCAAACATAAAC |
|  | shRNA 6968 for  CTAGGTTTATGTTTGGATGAACTGACATACGCGTATCCGTC﻿﻿TGTCAAAAGTATTGTCTGCGTAGTAGTGAAATATATATTAAAC﻿﻿﻿﻿TACGCAGACAATACTTTTGACTTACGGTAACGCGG |
|  | shRNA 6968 rev  AATTCCGCGTTACCGTA﻿﻿﻿﻿﻿AGTCAAAAGTATTGTCTGCGTAGTTTAATATATATTTCACTAC﻿﻿﻿﻿TACGCAGACAATACTTTTGACAGACGGATACGCGTATGTCAGTTCATCCAAACATAAAC |
| BRLF1 (p6841) | shRNA 6998 top  CTAGGTTTATGTTTGGATGAACTGACATACGCGTATCCGTC﻿﻿TTTACTATAACTACATTCAGGGGTAGTGAAATATATATTAAAC﻿﻿﻿﻿CCCTGAATGTAGTTATAGTAATTACGGTAACGCGG |
|  | shRNA 6998 bot  AATTCCGCGTTACCGTA﻿﻿﻿﻿﻿ATTACTATAACTACATTCAGGGGTTTAATATATATTTCACTAC﻿﻿﻿﻿CCCTGAATGTAGTTATAGTAAAGACGGATACGCGTATGTCAGTTCATCCAAACATAAAC |
|  | shRNA 6999 top  CTAGGTTTATGTTTGGATGAACTGACATACGCGTATCCGTC﻿﻿﻿TCATCATTTAGAAATGTATCCAGTAGTGAAATATATATTAAAC﻿﻿﻿﻿﻿﻿TGGATACATTTCTAAATGATGTTACGGTAACGCGG |
|  | shRNA 6999 bot  AATTCCGCGTTACCGTA﻿﻿﻿﻿﻿﻿ACATCATTTAGAAATGTATCCAGTTTAATATATATTTCACTAC﻿TGGATACATTTCTAAATGATGAGACGGATACGCGTATGTCAGTTCATCCAAACATAAAC |
|  | shRNA 7000 top  CTAGGTTTATGTTTGGATGAACTGACATACGCGTATCCGTC﻿﻿﻿TACTATAACTACATTCAGGGATGTAGTGAAATATATATTAAAC﻿﻿﻿﻿﻿﻿ATCCCTGAATGTAGTTATAGTTTACGGTAACGCGG |
|  | shRNA 7000 bot  AATTCCGCGTTACCGTA﻿﻿﻿﻿﻿﻿AACTATAACTACATTCAGGGATGTTTAATATATATTTCACTAC﻿﻿ATCCCTGAATGTAGTTATAGTAGACGGATACGCGTATGTCAGTTCATCCAAACATAAAC |
| BALF1 (p6632) | shRNA 6986 top  CTAGGTTTATGTTTGGATGAACTGACATACGCGTATCCGTC﻿TTCATTTACAAAGATTTCAGGAGTAGTGAAATATATATTAAAC﻿﻿﻿TCCTGAAATCTTTGTAAATGATTACGGTAACGCGG |
|  | shRNA 6986 bot  AATTCCGCGTTACCGTA﻿﻿﻿ATCATTTACAAAGATTTCAGGAGTTTAATATATATTTCACTAC﻿﻿TCCTGAAATCTTTGTAAATGAAGACGGATACGCGTATGTCAGTTCATCCAAACATAAAC |
|  | shRNA 6987 top  CTAGGTTTATGTTTGGATGAACTGACATACGCGTATCCGTCTATTCATTTACAAAGATTTCAGGTAGTGAAATATATATTAAAC﻿﻿﻿﻿CTGAAATCTTTGTAAATGAATTTACGGTAACGCGG |
|  | shRNA 6987 bot  AATTCCGCGTTACCGTA﻿﻿﻿AATTCATTTACAAAGATTTCAGGTTTAATATATATTTCACTAC﻿﻿CTGAAATCTTTGTAAATGAATAGACGGATACGCGTATGTCAGTTCATCCAAACATAAAC |
|  | shRNA 6988 top  CTAGGTTTATGTTTGGATGAACTGACATACGCGTATCCGTC﻿TTTACAAAGATTTCAGGAAGTCGTAGTGAAATATATATTAAAC﻿﻿﻿﻿GACTTCCTGAAATCTTTGTAATTACGGTAACGCGG |
|  | shRNA 6988 bot  AATTCCGCGTTACCGTA﻿﻿﻿﻿ATTACAAAGATTTCAGGAAGTCGTTTAATATATATTTCACTAC﻿GACTTCCTGAAATCTTTGTAAAGACGGATACGCGTATGTCAGTTCATCCAAACATAAAC |
| BBRF2 (p6639) | shRNA 6989 top (same as 6978 top)  CTAGGTTTATGTTTGGATGAACTGACATACGCGTATCCGTC﻿TAAAGAATCTTGATAAAACAGGGTAGTGAAATATATATTAAAC﻿﻿﻿CCTGTTTTATCAAGATTCTTTTTACGGTAACGCGG |
|  | shRNA 6989 bot (same as 6978 bot)  AATTCCGCGTTACCGTA﻿﻿﻿﻿AAAAGAATCTTGATAAAACAGGGTTTAATATATATTTCACTAC﻿﻿﻿CCTGTTTTATCAAGATTCTTTAGACGGATACGCGTATGTCAGTTCATCCAAACATAAAC |
|  | shRNA 6990 top (same as 6979 top)  CTAGGTTTATGTTTGGATGAACTGACATACGCGTATCCGTC﻿﻿TTGGCATTCTCAATCATCGAGAGTAGTGAAATATATATTAAAC﻿﻿﻿﻿﻿TCTCGATGATTGAGAATGCCATTACGGTAACGCGG |
|  | shRNA 6990 bot (same as 6979 bot)  AATTCCGCGTTACCGTA﻿﻿﻿﻿﻿ATGGCATTCTCAATCATCGAGAGTTTAATATATATTTCACTAC﻿﻿﻿﻿TCTCGATGATTGAGAATGCCAAGACGGATACGCGTATGTCAGTTCATCCAAACATAAAC |
|  | shRNA 6991 top  CTAGGTTTATGTTTGGATGAACTGACATACGCGTATCCGTC﻿﻿TTGGTCAATAAAGAATCTTGATGTAGTGAAATATATATTAAAC﻿﻿﻿﻿﻿ATCAAGATTCTTTATTGACCATTACGGTAACGCGG |
|  | shRNA 6991 bot  AATTCCGCGTTACCGTA﻿﻿﻿﻿﻿ATGGTCAATAAAGAATCTTGATGTTTAATATATATTTCACTAC﻿ATCAAGATTCTTTATTGACCAAGACGGATACGCGTATGTCAGTTCATCCAAACATAAAC |
| BMLF1 (p6838) | shRNA 6995 top  CTAGGTTTATGTTTGGATGAACTGACATACGCGTATCCGTC﻿﻿TTGTAATTCTTGATGTAGTGGCGTAGTGAAATATATATTAAAC﻿﻿﻿﻿GCCACTACATCAAGAATTACATTACGGTAACGCGG |
|  | shRNA 6995 bot  AATTCCGCGTTACCGTA﻿﻿﻿﻿ATGTAATTCTTGATGTAGTGGCGTTTAATATATATTTCACTAC﻿﻿﻿GCCACTACATCAAGAATTACAAGACGGATACGCGTATGTCAGTTCATCCAAACATAAAC |
|  | shRNA 6996 top  CTAGGTTTATGTTTGGATGAACTGACATACGCGTATCCGTC﻿TTTATTGATTTAATCCAGGAACGTAGTGAAATATATATTAAAC﻿﻿﻿﻿﻿GTTCCTGGATTAAATCAATAATTACGGTAACGCGG |
|  | shRNA 6996 bot  AATTCCGCGTTACCGTA﻿﻿﻿﻿ATTATTGATTTAATCCAGGAACGTTTAATATATATTTCACTAC﻿﻿﻿GTTCCTGGATTAAATCAATAAAGACGGATACGCGTATGTCAGTTCATCCAAACATAAAC |
|  | shRNA 6997 top  CTAGGTTTATGTTTGGATGAACTGACATACGCGTATCCGTC﻿﻿TTCACAAAGTTGTAGTCTCGCGGTAGTGAAATATATATTAAAC﻿﻿﻿﻿﻿CGCGAGACTACAACTTTGTGATTACGGTAACGCGG |
|  | shRNA 6997 bot  AATTCCGCGTTACCGTA﻿﻿﻿﻿﻿ATCACAAAGTTGTAGTCTCGCGGTTTAATATATATTTCACTAC﻿﻿CGCGAGACTACAACTTTGTGAAGACGGATACGCGTATGTCAGTTCATCCAAACATAAAC |
|  | Antisense/target: red-colored (top, for) and brown-colored (bot, rev)  Sense/passenger: green-colored (top, for) and blue-colored (bot, rev) |
