# Supplementary material for: Assessing the fitness of Epstein-Barr virus following its reactivation
Source: J Virol. 2025 May 30;99(7):e00626-25. doi: 10.1128/jvi.00626-25 (PMC12282151; doi:10.1128/jvi.00626-25)
Supplement: Table S3 — Synthetic pairs of oligonucleotides used for the construction of luciferase reporter plasmids. [file jvi.00626-25-s0005.docx]

**Table S3. Synthetic pairs of oligonucleotides used for the construction of luciferase reporter plasmids**

| Target gene (plasmid #) | Oligonucleotide |
| --- | --- |
| BALF4 (p7204) | 7204 for (sh6515-2, BALF4)  TCGAGATGGTGTTTAAAGACAACATTAAATTTGCAGACCTTCATCTCACTAAAAATT﻿GAAGCCATTACATATTTTATAAGC |
|  | 7204 rev (sh6515-2, BALF4)  GGCC﻿GC﻿TTATAAAATATGTAATGGCTTCAATTTTTAGTGAGATGAAGGTCTGCAAATTTAATGTTGTCTTTAAACACCATC |
| BKRF4 (p7329) | 7329 top (sh6562, BKRF4)  TCGAG﻿﻿﻿﻿﻿﻿﻿CCCAATACATACAATAAACACAAATT﻿﻿﻿AAGTCCCAATACATACAATAAAAATT﻿﻿﻿TCAAGTCCCAATACATACAATAGC |
|  | 7329 bot (sh6562, BKRF4)  GGCC﻿GC﻿﻿﻿TATTGTATGTATTGGGACTTGAAATTTTTATTGTATGTATTGGGACTTAATT﻿TGTGTTTATTGTATGTATTGGGC |
| BVLF1 (p7330) | 7330 top (sh6846, BVLF1)  TCGAG﻿﻿﻿﻿﻿﻿﻿CCCCGTGCGATGAGTTTATTTAAATT﻿﻿﻿TCCTCCTGAAGATGCTAAAGAAAATT﻿﻿﻿TGACCCTAAGTGTTAACTTTTAGC |
|  | 7330 bot (sh6846, BVLF1)  GGCC﻿GC﻿﻿﻿TAAAAGTTAACACTTAGGGTCAAATTTTCTTTAGCATCTTCAGGAGGAAATTTAAATAAACTCATCGCACGGGGC |
| BNLF2a (p7331) | 7331 top (sh6839, BNLF2a)  TCGAG﻿﻿﻿﻿﻿﻿﻿GACTTGTGATGCAATAAATAAAAATT﻿﻿﻿﻿TGACTTGTGATGCAATAAATAAAATT﻿﻿﻿ATGTGACTTGTGATGCAATAAAGC |
|  | 7331 bot (sh6839, BNLF2a)  GGCC﻿GC﻿﻿﻿TTTATTGCATCACAAGTCACATAATTTTATTTATTGCATCACAAGTCAAATTTTTATTTATTGCATCACAAGTCC |
| BXRF1 (p7332) | 7332 top (sh6848, BXRF1)  TCGAG﻿﻿﻿﻿﻿﻿﻿TCGCGGACAATAAGATTTACAAAATT﻿﻿﻿﻿GGTGTTATATTGTAGAATTTAAAATT﻿﻿﻿ACACCCTCTTTGAGATTATCAAGC |
|  | 7332 bot (sh6848, BXRF1)  GGCC﻿GC﻿﻿﻿TTGATAATCTCAAAGAGGGTGTAATTTTAAATTCTACAATATAACACCAATTTTGTAAATCTTATTGTCCGCGAC |
| BFLF2 (p7333) | 7333 top (sh6644, BFLF2)  TCGAG﻿﻿﻿﻿﻿﻿﻿CCCAGCTCATTTTGGAAAATAAAATT﻿﻿﻿﻿CCAGCTCATTTTGGAAAATAAAAATT﻿﻿﻿GACCTGGTACAGTCCTATCAAAGC |
|  | 7333 bot (sh6644, BFLF2)  GGCC﻿GC﻿﻿﻿TTTGATAGGACTGTACCAGGTCAATTTTTATTTTCCAAAATGAGCTGGAATTTTATTTTCCAAAATGAGCTGGGC |
| BMRF1 (p7334) | 7334 top (sh5106, BMRF1)  TCGAG﻿﻿﻿﻿﻿﻿﻿TGGTGACATTCATTAAATCCTAAATT﻿﻿﻿﻿GTTGGATCTTAGTGTTATTTTAAATT﻿﻿﻿﻿﻿TCACTATGGAATATGATGATAAGC |
|  | 7334 bot (sh5106, BMRF1)  GGCC﻿GC﻿﻿﻿TTATCATCATATTCCATAGTGAAATTTAAAATAACACTAAGATCCAACAATTTAGGATTTAATGAATGTCACCAC |
| BHLF1 (p7335) | 7335 top (sh7080, BHLF1)  TCGAG﻿﻿﻿﻿﻿﻿﻿CACCTATCAATAAACTGTTTAAAATT﻿﻿﻿﻿﻿﻿ACACTACTCACCTACATGTCAAAATT﻿﻿﻿﻿﻿GTACACTACACTCTAAAAGTAAGC |
|  | 7335 bot (sh7080, BHLF1)  GGCC﻿GC﻿﻿﻿TTACTTTTAGAGTGTAGTGTACAATTTTGACATGTAGGTGAGTAGTGTAATTTTAAACAGTTTATTGATAGGTGC |
| BXLF1 (p7337) | 7337 top (sh6571, BXLF1)  TCGAG﻿﻿﻿﻿﻿﻿﻿ACAGCATCTTTAGTGTACTTAAAATT﻿﻿﻿﻿﻿﻿ACCGCTACAAGTTAATTTACAAAATT﻿﻿TGGGCGCGAGTTTAGTAAGTTAGC |
|  | 7337 bot (sh6571, BXLF1)  GGCC﻿GC﻿﻿﻿TAACTTACTAAACTCGCGCCCAAATTTTGTAAATTAACTTGTAGCGGTAATTTTAAGTACACTAAAGATGCTGTC |
| BVRF2 (p7267) | 7267 for (sh6847, BVRF2)  TCGAG﻿﻿﻿﻿TCCCAGCCGAGTCGTATTTAAAAATT﻿﻿﻿CACAGGTAAAAGTTAACACTTAAATT﻿﻿﻿﻿﻿﻿﻿﻿﻿﻿﻿TACGGATTTCAGCCTCATCAAAGC |
|  | 7267 rev (sh6847, BVRF2)  GGCC﻿GC﻿﻿﻿TTTGATGAGGCTGAAATCCGTAAATTTAAGTGTTAACTTTTACCTGTGAATTTTTAAATACGACTCGGCTGGGAC |
| BALF2 (p7338) | 7338 top (sh6927, BALF2)  TCGAG﻿﻿﻿﻿﻿﻿﻿CGACCACATATGAGATTGAGAAAATT﻿﻿﻿﻿﻿﻿CCAGGAACATCAAGATCAAGAAAATT﻿﻿CCGAAGTGGTCCAGTTTATGAAGC |
|  | 7338 bot (sh6927, BALF2)  GGCC﻿GC﻿﻿﻿TTCATAAACTGGACCACTTCGGAATTTTCTTGATCTTGATGTTCCTGGAATTTTCTCAATCTCATATGTGGTCGC |
| BPLF1 (p7211) | 7211 for (sh6567-2, BPLF1)  TCGAGTGTATCTGTAATTGTTTATTAAAATTTCTGTAATTGTTTATTAATAAAAATT﻿CACCCAATTTCTGTCTATTGAAGC |
|  | 7211 rev (sh6567-2, BPLF1)  GGCC﻿GC﻿TTCAATAGACAGAAATTGGGTGAATTTTTATTAATAAACAATTACAGAAATTTTAATAAACAATTACAGATACAC |
| BBRF1 (p7212) | 7212 for (sh6593-4, BRRF1)  TCGAG﻿﻿CCTGTTTTATCAAGATTCTTTAAATT﻿﻿TCTCGATGATTGAGAATGCCAAAATT﻿CTGAGGTCATGTTCAACATGAAGC |
|  | 7212 rev (sh6593-4, BRRF1)  GGCC﻿GC﻿TTCATGTTGAACATGACCTCAGAATTTTGGCATTCTCAATCATCGAGAAATTTAAAGAATCTTGATAAAACAGGC |
| BGLF4 (p7209) | 7209 for (sh6561-2, BGLF4)  TCGAGTCCCAGTCAACATCTTCATCAAAATTAAGAGGTCATTTATCAGATTTAAATT﻿﻿﻿﻿﻿﻿﻿﻿GTGACTGTCATTGATTACTTCAGC |
|  | 7209 rev (sh6561-2, BGLF4)  GGCC﻿GC﻿﻿﻿﻿﻿﻿﻿﻿TGAAGTAATCAATGACAGTCACAATTTAAATCTGATAAATGACCTCTTAATTTTGATGAAGATGTTGACTGGGAC |
| BFRF1 (p7215) | 7215 for (sh6645-2, BFRF1)  TCGAG﻿﻿﻿﻿﻿GTGTATGCACTTTATTAATAAAAATT﻿﻿CGCCCGTGTTTGTGATATCCAAAATT﻿﻿﻿﻿﻿﻿TCCCCTGCTTCCTAAATTTCAAGC |
|  | 7215 rev (sh6645-2, BFRF1)  GGCC﻿GC﻿TTGAAATTTAGGAAGCAGGGGAAATTTTGGATATCACAAACACGGGCGAATTTTTATTAATAAAGTGCATACACC |
| BSLF1 (p7218) | 7218 for (sh6843-2, BSLF1)  TCGAG﻿﻿﻿CCCAGTCTATTTTTTCAAATCAAATT﻿﻿CCCCAGTGTACTATTCACAGAAAATT﻿﻿﻿﻿﻿﻿﻿﻿﻿﻿CAGGCACTACTTTGTAATGACAGC |
|  | 7218 rev (sh6843-2, BSLF1)  GGCC﻿GC﻿﻿﻿TGTCATTACAAAGTAGTGCCTGAATTTTCTGTGAATAGTACACTGGGGAATTTGATTTGAAAAAATAGACTGGGC |
| BTRF1 (p7219) | 7219 for (sh6845-2, BTRF1)  TCGAG﻿﻿﻿GGCACCATTATGAGAAATGTCAAATT﻿﻿CCATGGCACAGTTTCTAGGAAAAATT﻿﻿﻿﻿﻿﻿﻿﻿﻿﻿TCGGGACAGATTGTCTTCCACAGC |
|  | 7219 rev (sh6845-2, BTRF1)  GGCC﻿GC﻿﻿﻿TGTGGAAGACAATCTGTCCCGAAATTTTTCCTAGAAACTGTGCCATGGAATTTGACATTTCTCATAATGGTGCCC |
| BMRF2 (p7207) | 7207 for (sh6539-2, BMRF2)  TCGAGTGGTGACATTCATTAAATCCTAAATTACAAGCTTAAGAAGTTTGTGAAAATTTACAAGCTTAAGAAGTTTGTGAGC |
|  | 7207 rev (sh6539-2, BMRF2)  GGCC﻿GC﻿﻿﻿﻿TCACAAACTTCTTAAGCTTGTAAATTTTCACAAACTTCTTAAGCTTGTAATTTAGGATTTAATGAATGTCACCAC |
| BBRF3 (p7206) | 7206 for (sh6534-4, BRRF3)  TCGAGCTGGGCAACAGCTTTTACTTTAAATTCACGCCCTTTGCAAATTATTTAAATTGCCCTTTGCAAATTATTTAAAAGC |
|  | 7206 rev (sh6534-4, BRRF3)  GGCC﻿GC﻿﻿﻿﻿TTTTAAATAATTTGCAAAGGGCAATTTAAATAATTTGCAAAGGGCGTGAATTTAAAGTAAAAGCTGTTGCCCAGC |
| BNRF1 (p7210) | 7210 for (sh6564-2, BNRF1)  TCGAGTCCAGACTATGCATACACTGAAAATTACCTGTGTGCTGTATTTACAAAAATT﻿TCCCTTCGTTAATGCAATAGCAGC |
|  | 7210 rev (sh6564-2, BNRF1)  GGCC﻿GC﻿TGCTATTGCATTAACGAAGGGAAATTTTTGTAAATACAGCACACAGGTAATTTTCAGTGTATGCATAGTCTGGAC |
| BBLF1 (p7208) | 7208 for (sh6557-2, BBLF1)  TCGAGTCCCAGTCAACATCTTCATCAAAATTTGAGGACTCTGAAAATGATGAAAATT﻿TACGCAGACAATACTTTTGACAGC |
|  | 7208 rev (sh6557-2, BBLF1)  GGCC﻿GC﻿﻿﻿﻿﻿﻿﻿﻿TGTCAAAAGTATTGTCTGCGTAAATTTTCATCATTTTCAGAGTCCTCAAATTTTGATGAAGATGTTGACTGGGAC |
| BRLF1 (p7217) | 7217 for (sh6841-2, BRLF1)  TCGAG﻿﻿﻿CCCTGAATGTAGTTATAGTAAAAATT﻿﻿TGGATACATTTCTAAATGATGAAATTATCCCTGAATGTAGTTATAGTAGC |
|  | 7217 rev (sh6841-2, BRLF1)  GGCC﻿GC﻿﻿﻿TACTATAACTACATTCAGGGATAATTTCATCATTTAGAAATGTATCCAAATTTTTACTATAACTACATTCAGGGC |
| BALF1 (p7213) | 7213 for (sh6632-2, BALF1)  TCGAG﻿﻿﻿TCCTGAAATCTTTGTAAATGAAAATT﻿﻿CTGAAATCTTTGTAAATGAATAAATTGACTTCCTGAAATCTTTGTAAAGC |
|  | 7213 rev (sh6632-2, BALF1)  GGCC﻿GC﻿TTTACAAAGATTTCAGGAAGTCAATTTATTCATTTACAAAGATTTCAGAATTTTCATTTACAAAGATTTCAGGAC |
| BBRF2 (p7214) | 7214 for (sh6639-2, BBRF2)  TCGAG﻿﻿﻿CCTGTTTTATCAAGATTCTTTAAATT﻿﻿TCTCGATGATTGAGAATGCCAAAATT﻿﻿﻿ATCAAGATTCTTTATTGACCAAGC |
|  | 7214 rev (sh6639-2, BBRF2)  GGCC﻿GC﻿TTGGTCAATAAAGAATCTTGATAATTTTGGCATTCTCAATCATCGAGAAATTTAAAGAATCTTGATAAAACAGGC |
| BMLF1 (p7216) | 7216 for (sh6838-2, BMLF1)  TCGAG﻿﻿﻿GCCACTACATCAAGAATTACAAAATT﻿﻿GTTCCTGGATTAAATCAATAAAAATT﻿﻿﻿﻿﻿﻿CGCGAGACTACAACTTTGTGAAGC |
|  | 7216 rev (sh6838-2, BMLF1)  GGCC﻿GC﻿TTCACAAAGTTGTAGTCTCGCGAATTTTTATTGATTTAATCCAGGAACAATTTTGTAATTCTTGATGTAGTGGCC |
|  | Sense/target: three individual red-colored sequences  Antisense: three individual brown-colored sequences |
